# Amide synthesis *via* nickel-catalysed reductive aminocarbonylation of aryl halides with nitroarenes†
†Electronic supplementary information (ESI) available: Experimental and spectra data. See DOI: 10.1039/c7sc03950f


**DOI:** 10.1039/c7sc03950f

**Published:** 2017-11-06

**Authors:** Chi Wai Cheung, Marten Leendert Ploeger, Xile Hu

**Affiliations:** a Laboratory of Inorganic Synthesis and Catalysis , Institute of Chemical Sciences and Engineering , Ecole Polytechnique Fédérale de Lausanne (EPFL) , ISIC-LSCI , BCH 3305 , Lausanne 1015 , Switzerland . Email: xile.hu@epfl.ch ; http://lsci.epfl.ch

## Abstract

Aminocarbonylation of aryl halides is one of the most useful methods in amide synthesis, but nitroarenes have not been used as a nitrogen source in this method even though they are more economical and accessible than anilines. Reported here is the development of nickel catalysis for the first three-component reactions of aryl halides, Co_2_(CO)_8_, and nitroarenes under reductive conditions to produce aryl amides. A wide range of (hetero)aryl iodides and bromides as well as nitro(hetero)arenes are suitable reaction partners, and high functional group compatibility has been achieved. The method might be used for the streamlined synthesis of aryl amides.

## Abstract 2

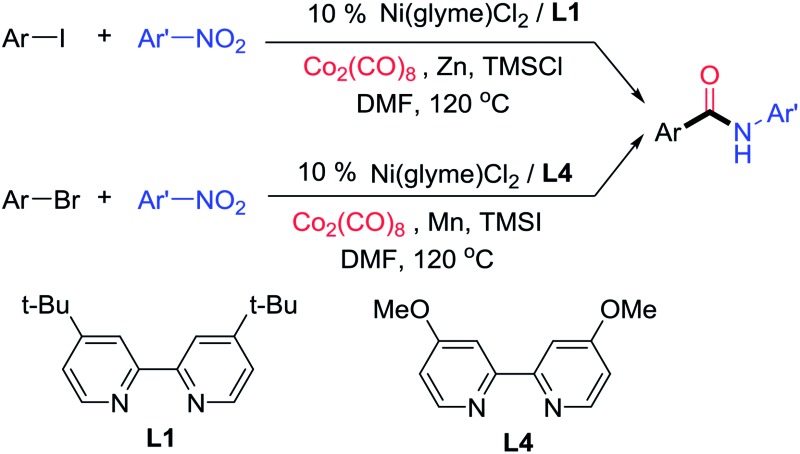
A nickel-catalysed reductive aminocarbonylation of (hetero)aryl halides employing readily available nitro(hetero)arenes as the nitrogen source has been developed.

## 

Amides are an essential structural motif in numerous natural and synthetic bioactive compounds as well as organic materials.^1^,^2^ One of the most commonly used methods to synthesize aryl amides is palladium-catalyzed aminocarbonylation of aryl halides^3^,^4^ (Fig. 1a). Typically an alkyl amine or aniline is used as the nitrogen source. Because nitroarenes are generally less expensive than the corresponding anilines and most anilines are prepared by reduction of the corresponding nitroarenes, the direct use of nitroarenes will save reagent cost and eliminate at least one process step. Moreover, the nitro group exhibits orthogonal reactivity to the amine group, so functional groups that are typically incompatible with nucleophilic amination reactions, such as ketones, esters, alkyl halides, and alcohols, might be tolerated using nitroarenes as the starting reagents. While a number of studies have emerged to exploit the advantages of nitroarenes as a nitrogen source,^5^ there has been no previous report on using nitroarenes in the aminocarbonylation of aryl halides. Only a few examples of aminocarbonylation of other carbon nucleophiles are known. For example, Beller and co-workers reported a Pd-catalyzed aminocarbonylation of olefins with nitroarenes using dihydrogen as reductant (Fig. 1b).^6^ The nitroarenes appeared to be reduced to anilines *in situ* in those reactions. Driver and co-workers reported a Pd-catalyzed, directing-group-assisted aminocarbonylation of aryl C–H bonds with nitroarenes and molybdenum hexacarbonyl (Mo(CO)_6_) (Fig. 1c).^7^ Interestingly, nitroarenes were proposed to be first reduced to nitrosoarenes, which directly took part in the aminocarbonylation without being reduced to anilines. Nevertheless, due to the requirement of a strong directing group, the carbon nucleophiles were limited to pyridine, pyrimidine or indazole-substituted 2-aryl groups. The scope of nitroarenes was modest as well, as *ortho*-substitution was not tolerated and no examples of nitro(hetero)arenes were reported. Here we report the first aminocarbonylation of aryl halides with nitroarenes using abundant and commercially available nickel catalysts (Fig. 1d). It should be noted that nickel catalysis has hardly been used for aminocarbonylation in general. To our knowledge, only one example of a Ni-catalyzed aminocarbonylation of aryl iodides with amines has been previously reported.^8^ Our method has broad scope and high functional group compatibility, allowing the rapid synthesis of a diverse class of aryl amides.

**Fig. 1:**
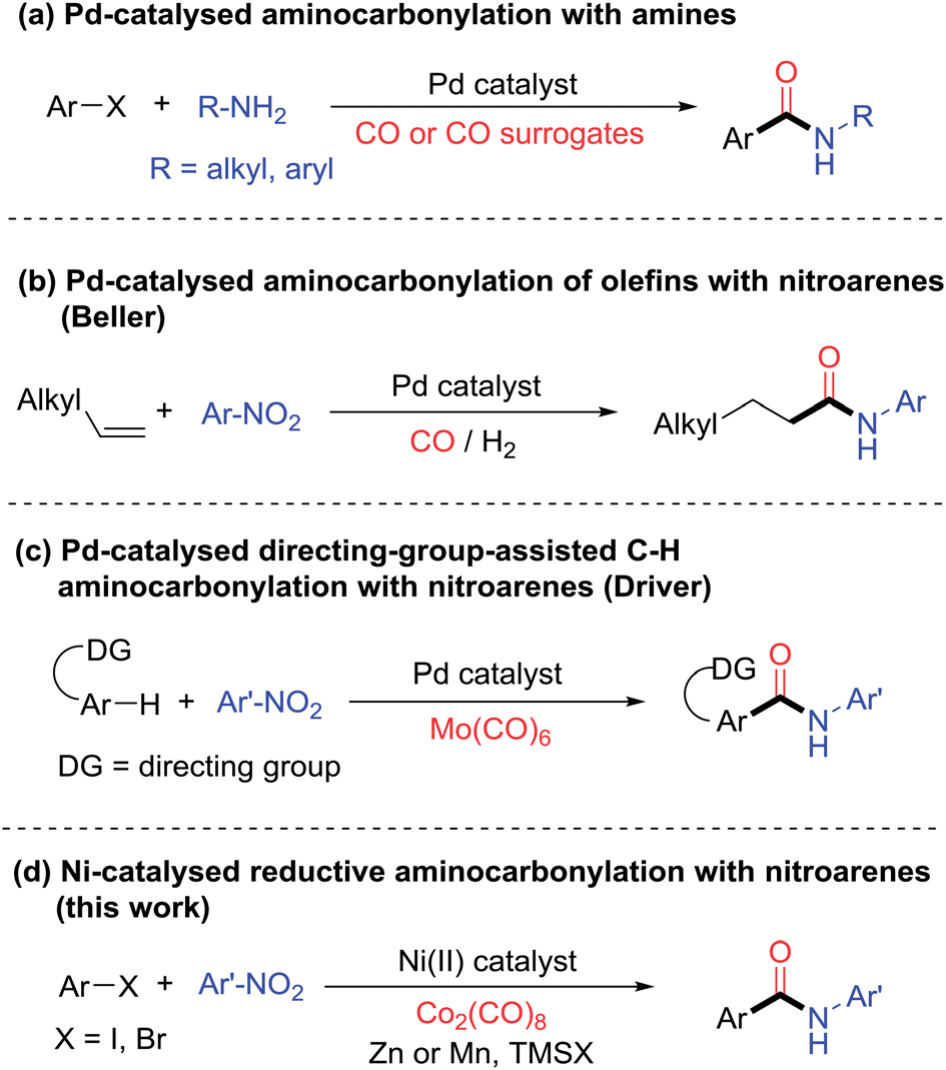
Various methods of aminocarbonylation.

We recently reported that, under reductive conditions, nitroarenes could be transformed into nitrosoarenes5*d* and diazoarenes,5e,f which served as the nitrogen sources in the Fe-catalyzed amination of alkyl halides,5*d* and Ni-catalyzed amidation of esters5*e* and transamidation,5*f* respectively. We hypothesized that a Ni-catalyzed aminocarbonylation of aryl halides with nitroarenes might operate following the mechanistic pathway displayed in Fig. 2.^9^–^11^ Initially, a Ni(ii) precatalyst is reduced by an inexpensive reductant (Zn or Mn) to a Ni(0) species, which then activates an aryl halide *via* oxidative addition to give a Ni(ii)-aryl intermediate. The insertion of CO into the Ni(ii)-aryl species gives a Ni(ii)-acyl complex. Meanwhile, in the presence of a halotrimethylsilane additive (TMSX), a nitroarene is reduced by the reductant to form a nitrosoarene or a diazoarene, or other reduced species such as *N*-phenylhydroxylamine or aniline,5d–f which could then react with the Ni-acyl species to produce an amido anion which, upon acidic workup, furnished the desired amide. If aniline is involved, then the C–N bond forming step is analogous to that in a standard C–N coupling and a Ni(0) species is regenerated directly. If a less reduced nitrogen species is involved, then electron transfer from the metal reductant is necessary to regenerate the Ni(0) species, and the mechanism can be rather complicated. Nevertheless, previous work on Ni-catalyzed reductive amidation5*e* and transamidation5*f* suggests the feasibility of such a transformation.

**Fig. 2:**
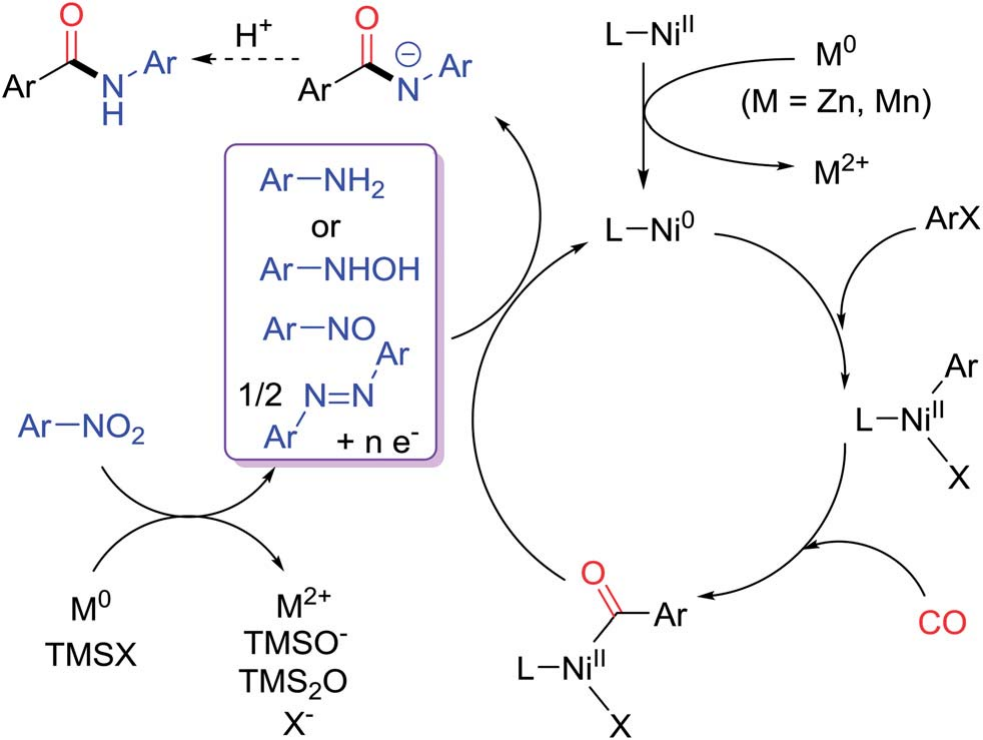
Mechanistic design of a Ni-catalyzed reductive aminocarbonylation of aryl halides with nitroarenes.

We commenced the study by examining the reaction of iodobenzene (**1a**) with 1-*tert*-butyl-4-nitrobenzene (**2a**) (Table 1). Because CO gas is inconvenient to handle in the setting of a synthetic laboratory, we decided to employ a metal polycarbonyl reagent as the CO surrogate. After screening the reaction parameters, we found that the optimized conditions involved the use of dimethylformamide (DMF) as solvent, Ni(glyme)Cl_2_ (10 mol%) as catalyst, 4,4′-di-*tert*-butyl-2,2′-dipyridyl (dtbpy, **L1**) as ligand, Zn powder (2 equiv.) as reductant, chlorotrimethylsilane (TMSCl, 10 mol%) as Zn-activating reagent, and dicobalt octacarbonyl (Co_2_(CO)_8_) as CO surrogate (0.8 equiv.) (Table 1, entry 1).^12^ The optimal loading of **2a** was 1.5 equiv., and the reaction was completed after 16 h at 120 °C. After an acidic workup, the desired amide product, *N*-4-*tert*-butylphenyl benzamide (**3**), was obtained quantitatively. The use of other bidentate nitrogen-based ligands, Mn reductant, and other polar aprotic solvents led to diminishment in the yields (Table 1, entries 2–6). Particularly, the use of other CO surrogates (Fe(CO)_6_ and Mn(CO)_6_, Table 1, entries 7 and 8), CO (entry 9), and other transition metal catalysts (iron, cobalt, copper and manganese, Table 1, entries 10–13) resulted in a significant drop in the yields. Without Ni(glyme)Cl_2_, only a trace of **2a** was formed, suggesting that Ni is the real catalyst and Co in Co_2_(CO)_8_ is not (Table 1, entry 14). **L1** was also essential for the reaction to significantly enhance the yield (Table 1, entry 15). Because some reduction of nitrobenzene to aniline occurred as a side reaction, a slight excess (1.5 equiv.) of nitrobenzene was needed.

**Table 1:** Optimization of the catalytic reductive aminocarbonylation of aryl iodide with nitroarene

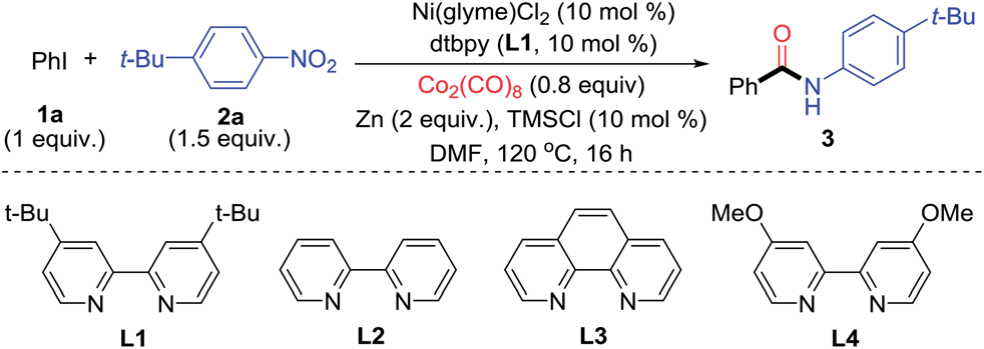		
Entry	Variations from ‘standard conditions’	Yield^*a*^
1	None	100
2	**L2** instead of **L1**	92
3	**L3** instead of **L1**	91
4	**L4** instead of **L1**	87
5	Mn (2 equiv.) instead of Zn	58
6	NMP instead of DMF	85
7	Fe(CO)_5_ (2 equiv.) instead of Co_2_(CO)_8_	26
8	Mo(CO)_6_ (2 equiv.) instead of Co_2_(CO)_8_	31
9	CO (1.4–2.4 bar) instead of Co_2_(CO)_8_	6–16
10	FeBr_2_ (10 mol%) instead of Ni(glyme)Cl_2_	10
11	CoCl_2_ (10 mol%) instead of Ni(glyme)Cl_2_	<5
12	CuBr_2_ (10 mol%) instead of Ni(glyme)Cl_2_	<5
13	MnCl_2_ (10 mol%) instead of Ni(glyme)Cl_2_	<5
14	No Ni(glyme)Cl_2_	<5
15	No **L1**	55

^*a*^Corrected GC yield using *n*-dodecane as an internal standard.

The optimization conditions in Table 1 could be applied for the aminocarbonylation of various aryl iodides (Fig. 3). Electron-neutral (**3a**), -rich (**3h** and **3n–3p**), and -deficient aryl iodides (**3r–3v**), as well as six- (pyridyl, **3dd**) and five-membered (pyrazolyl, **3ee**) (hetero)aryl iodides, were all suitable coupling partners. Likewise, electron-rich (**3a**, **3f**, **3j**, **3n**, **3p**, **3r**, **3t**, **3aa** and **3bb**), neutral- (**3ff**), and -deficient nitroarenes (**3b**, **3h** and **3w**) also reacted smoothly. In particular, a wide range of nitro(hetero)arenes could be used, including pyridine (**3e** and **3k**), pyrrole (**3f**), N–H free indole (**3g**), benzothiazole (**3l**), coumarin (**3m**), carbazole (**3p**), pyrazole (**3q**), oxazole (**3x**), and benzoxazole (**3cc**). The sterically bulky aryl iodide (**3q**) and nitroarenes (**3i** and **3j**) also reacted equally well. *ortho*-Substitution on the nitroarene was tolerated (**3i** and **3j**), which is a significant improvement in scope compared to the Pd-catalyzed aminocarbonylation method developed by Driver.^7^ A broad range of functional groups were compatible on both the aryl iodide and nitroarene substrates, such as thio (**3a** and **3t**), fluoro (**3b**, **3e** and **3s**), chloro (**3c**, **3r** and **3gg**), bromo (**3d**), ether (**3c** and **3h**), trifluoromethoxy (**3t**), trifluoromethyl (**3u** and **3y**), nitrile (**3h** and **3v**), olefin (**3n**), tosyl (**3w**) and amino (**3bb**) groups, and protected functional groups such as aldehyde (**3y**), ketone (**3z**), and alcohol (**3aa**) groups. When the optimal conditions in Table 1 were applied for the aminocarbonylation of bromobenzene (**1b**) with **2a**, the yield of *N-t-tert*-butylphenyl benzamide was only 5% (Table 2, entry 1). Further optimization showed that by replacing Zn with Mn (5 equiv.), TMSCl with iodotrimethylsilane (TMSI, 1.5 equiv.), and **L1** with 4,4′-dimethoxy-2,2′-dipyridyl (**L4**), and by enhancing the loading of Co_2_(CO)_8_ to 1.2 equiv., the desired amide product **3** could be obtained in 87% yield (Table 2, entry 2). The use of other ligands, reductant, additives, and lower loadings of TMSI and Co_2_(CO)_8_ resulted in significant diminishment in the yields (Table 2, entries 3–9). Again, both the Ni catalyst and **L4** were essential (Table 2, entries 10 and 11). The use of Mn instead of Zn led to a significant enhancement of the yield (Table 2, entries 2 and 5). We hypothesize that a higher reducing power from Mn might be necessary to reduce the relevant Ni(ii) species to Ni(0), and a more electron-rich ligand **L4** might be essential to promote the oxidative addition of an aryl-bromide bond.

**Table 2:** Optimization of the catalytic reductive aminocarbonylation of aryl bromide with nitroarene

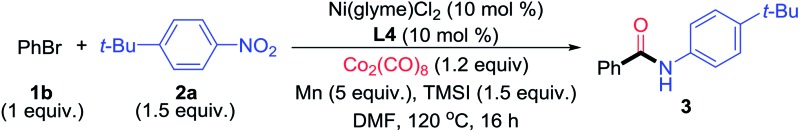		
Entry	Variations from ‘standard conditions’	Yield^*a*^
1	Table 1, entry 1	5
2	None	87
3	**L1** instead of **L4**	65
4	**L3** instead of **L4**	51
5	Zn (5 equiv.) instead of Mn	8
6	TMSBr (1.5 equiv.) instead of TMSI	27
7	TMSCl (1.5 equiv.) instead of TMSI	31
8	TMSI (1 equiv.) instead of (1.5 equiv.)	74
9	Co_2_(CO)_8_ (1 equiv.) instead of (1.2 equiv.)	65
10	No Ni(glyme)Cl_2_	36
11	No **L4**	69

^*a*^Corrected GC yield using *n*-dodecane as an internal standard.

**Fig. 3:**
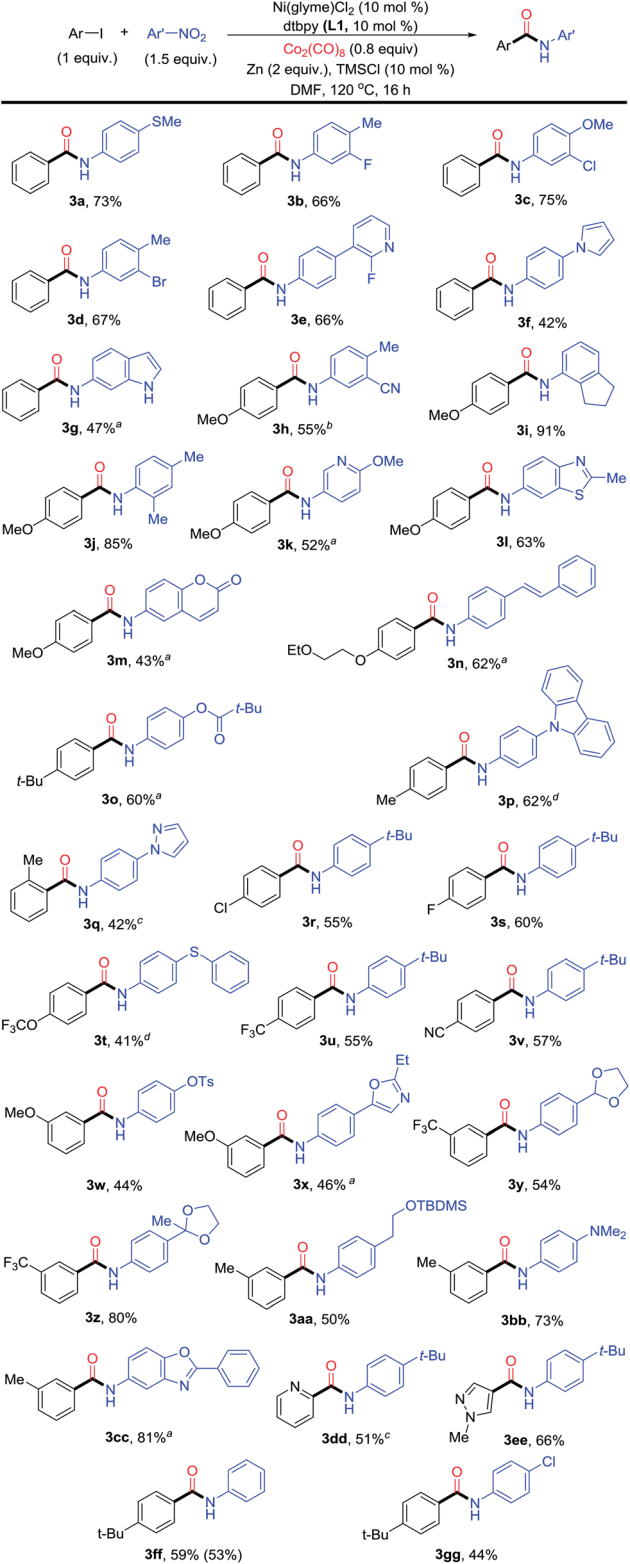
Scope of the Ni-catalyzed reductive aminocarbonylation of aryl iodides with nitroarenes. ^*a*^Ni(glyme)Cl_2_ (15 mol%), **L1** (15 mol%), Co_2_(CO)_8_ (1 equiv.) and DMF (1.4 mL). ^*b*^Co_2_(CO)_8_ (1 equiv.). ^*c*^Ni(glyme)Cl_2_ (15 mol%), **L1** (15 mol%) and Co_2_(CO)_8_ (1 equiv.). ^*d*^DMF (1.4 mL).

The conditions in Table 2 were then applied for the aminocarbonylation of various aryl bromides (Fig. 4). Both electron-rich (**4a**) and -deficient (**4b–4e**, **4g** and **4h**) aryl bromides coupled efficiently. Various (hetero)aryl bromides were also suitable coupling partners, including quinoline (**4j**), indole (**4k**), indazole (**4l**), benzofuran (**4m**), carbazole (**4n**), and benzothiophene (**4o**). Naphthyl (**4i**) could be adopted as substrate as well. Additionally, electron-rich (**4b–4f**, **4h**, **4j** and **4m**) and -deficient nitroarenes (**4i**), nitro(hetero)arenes (quinoline (**4a**) and 1,3-benzodioxole (**4f**)), polycyclic nitroarene (**4h**), and sterically bulky nitroarene (**4p**) also reacted equally well. A wide range of functional groups were compatible on both the aryl bromide and nitroarene substrates, such as ethers (**4a**, **4c** and **4m**), fluoro (**4b** and **4p**), amine (**4b** and **4f**), ketone (**4c** and **4g**), amide (**4d**) and trifluoromethyl (**4e**, **4h** and **4i**). Aminocarbonylation also worked for a vinyl bromide (**4p**). Alkyl halides, on the other hand, could not be used as substrates.

**Fig. 4:**
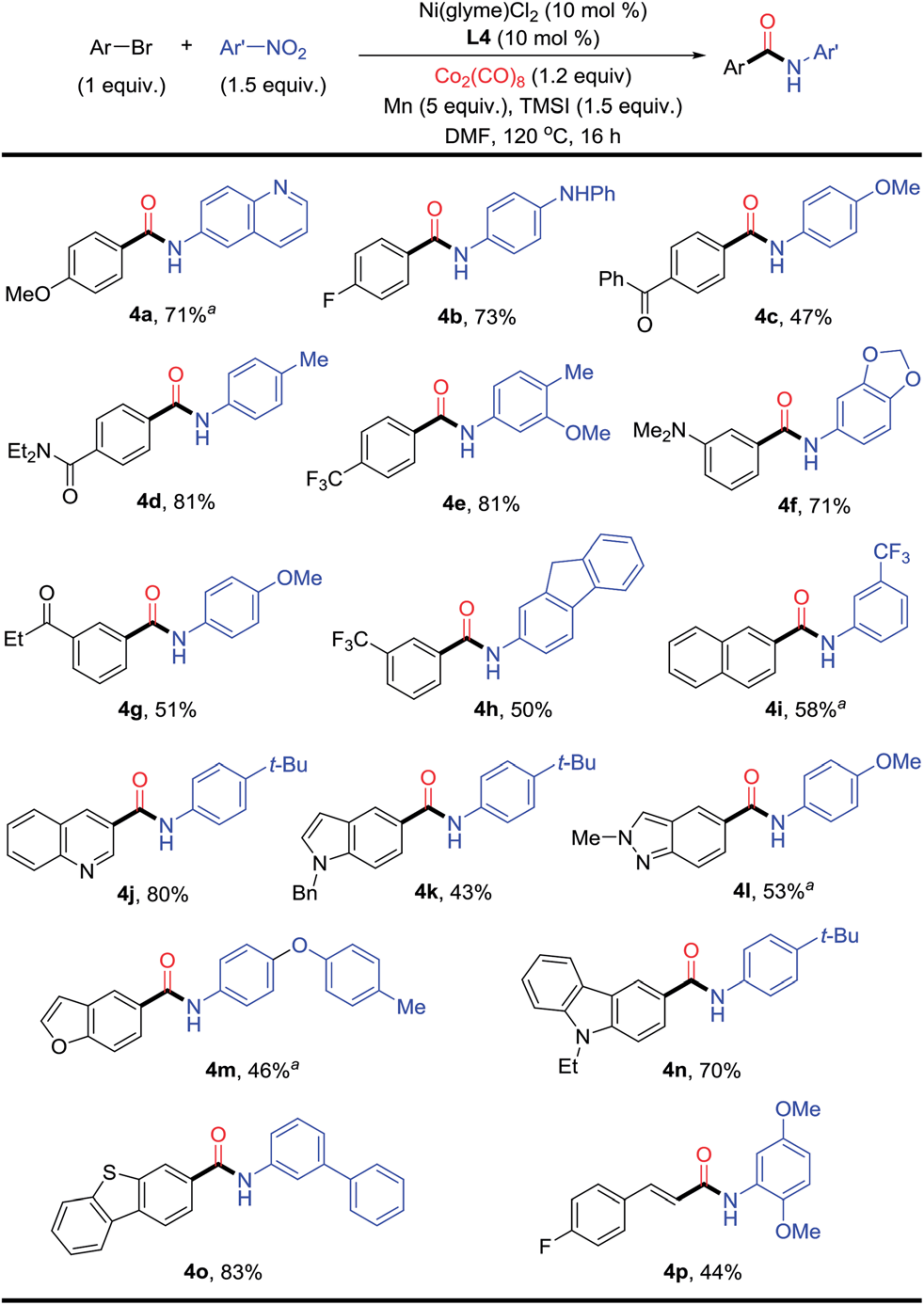
Scope of the Ni-catalyzed reductive aminocarbonylation of aryl bromides with nitroarenes. ^*a*^Ni(glyme)Cl_2_ (15 mol%) and **L4** (15 mol%).

To demonstrate an application, the current aminocarbonylation method was applied for the synthesis of a bioactive molecule, **5**, which is a potent allosteric modulator of metabotropic glutamate receptor 4 (mGluR4) for the potential treatment of Parkinson’s disease (Fig. 5).^13^ Indeed, our method allowed the synthesis of **5** in 63% yield.

**Fig. 5:**
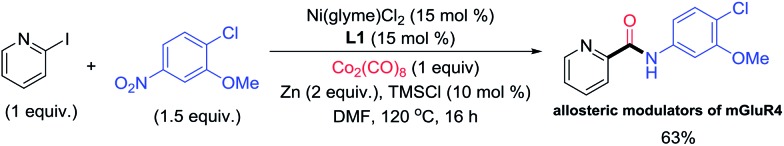
Application of reductive aminocarbonylation in the synthesis of the bioactive molecule **5**.

To probe the nature of the nitrogen-containing intermediate in the reductive aminocarbonylation, reactions with viable intermediates from the reduction of nitrobenzene were studied. Nitrobenzene could be reduced to nitrosobenzene, *N*-phenyl hydroxylamine, azobenzene, and anilines under reductive conditions5d–f (Fig. 2). In the test reaction using nitrobenzene, the desired aryl amide was formed in 55% yield (Fig. 6a). While nitrosobenzene and azobenzene only reacted to give a low yield of product (<20%) (Fig. 6b(i) and (ii)), *N*-phenyl hydroxylamine and aniline reacted to give the products in 56% and 48% yields, respectively (Fig. 6b(iii) and (iv)), which were comparable to those of the parent reaction (Fig. 6a). It is noted that the aminocarbonylation of *tert*-butylaniline has yields 30–50% lower than reactions using its corresponding nitroarene compound (Table S1,† entries 28–29). The difference in yields might be due to different effective concentrations of the reagents (anilines, Zn and Co_2_(CO)_8_) in the two protocols. Nitrobenzene might be reduced by CO to form phenyl isocyanate.^7^,^14^ However, phenyl isocyanate reacted to give only a trace of product (Fig. 6b(v)). Azobenzene and phenyl isocyanate are also incompatible with conditions simulating the (partial) consumption of dicobalt octacarbonyl (Table S3†). Thus, based on the reactivity studies of the possible intermediates derived from nitrobenzene, both *N*-phenyl hydroxylamine and aniline are possible intermediates in the reductive aminocarbonylation reaction. The direct reaction of nitrobenzene cannot be ruled out either. A more detailed mechanism of this aminocarbonylation reaction is subject to further study.

**Fig. 6:**
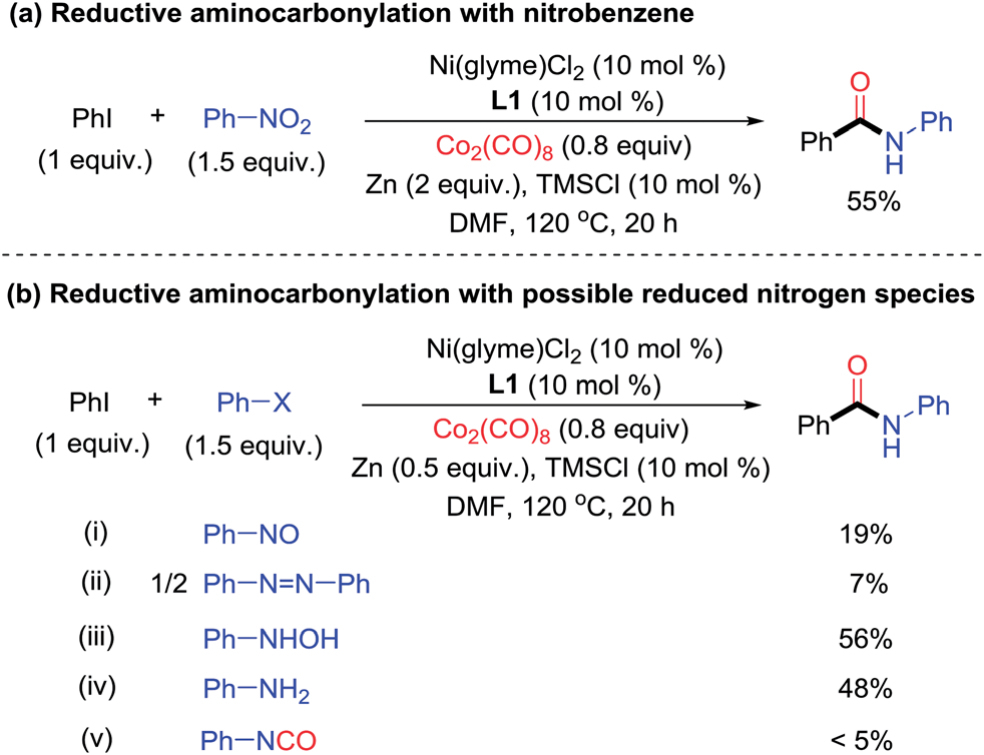
Studies of the nitrogen-containing intermediate in the Ni-catalyzed reductive aminocarbonylation of aryl halide with nitrobenzene.

## Conclusions

In conclusion, a new Ni-catalyzed methodology has been developed to enable the first aminocarbonylation of (hetero)aryl iodides and bromides with nitro(hetero)arenes. Its broad scope and high group compatibility have been demonstrated. The direct use of nitroarenes in the place of anilines provides potential advantages in the cost and step economy. Its application in the streamlined synthesis of aryl amides can be anticipated.

## Conflicts of interest

There are no conflicts to declare.

## Supplementary Material

Supplementary information
