# Supplementary material for: Amide synthesis via nickel-catalysed reductive aminocarbonylation of aryl halides with nitroarenes
Source: Chem Sci. 2017 Nov 6;9(3):655–9. doi: 10.1039/c7sc03950f (PMC5869604; doi:10.1039/c7sc03950f)

Electronic Supplementary Material (ESI) for Chemical Science.
This journal is © The Royal Society of Chemistry 2017

# Supporting Information

## Amide Synthesis via Nickel-Catalysed Reductive Aminocarbonylation of Aryl Halides with Nitroarenes

Chi Wai Cheung, Marten Leendert Ploeger, and Xile Hu*

Laboratory of Inorganic Synthesis and Catalysis
Institute of Chemical Sciences and Engineering
Ecole Polytechnique Fédérale de Lausanne (EPFL)
ISIC-LSCI, BCH 3305, Lausanne 1015 (Switzerland)

E-mail: xile.hu@epfl.ch


| Content | Page no. |
|---|---|
| General Considerations | S2 |
| Supplementary Data | S5 |
| Experimental Section | S11 |
| References | S32 |
| NMR Spectra | S33 |

# General Considerations

## (A) General Analytical Information.

Nuclear Magnetic Resonance spectra were recorded on a Bruker Avance 400 MHz instruments at ambient temperature. All $^1$H NMR spectra were measured in part per million (ppm) relative to the signal of tetramethylsilane (TMS) added into the deuterated chloroform ($CDCl_3$, 0.00 ppm), or the signal of residual dichloromethane in deuterated dichloromethane ($CD_2Cl_2$, 5.32 ppm).[1,2] Data for $^1$H NMR were reported as follows: chemical shift, multiplicity (s = singlet, d = doublet, t = triplet, q = quartet, qu = quintet, sex = sextet, m = multiplet, ovrlp = overlap, br = broad), coupling constants, and integration. All $^{13}$C NMR spectra were reported in ppm relative to $CDCl_3$ (77.16 ppm), $CD_2Cl_2$ (53.84 ppm), or DMSO-$d_6$ (39.52 ppm)[1,2] and were obtained with complete $^1$H decoupling. All gas chromatography (GC) analyses were performed on a Perkin-Elmer Clarus 400 GC system with a FID detector. All gas chromatography-mass spectrometry (GC-MS) analyses were performed on an Agilent Technologies 7890A GC system equipped with a 5975C MS detector. High-resolution mass spectra (HRMS) by electrospray ionization (ESI) method were performed at the EPFL ISIC Mass Spectroscopy Service with a Micro Mass QTOF Ultima spectrometer.

## (B) General Reagent Information.

Unless otherwise noted, all chemicals were used as received without further purifications. Dimethylformamide (DMF) was purified and dehydrated using a two-column solid-state purification system (Innovative Technology, NJ, USA) and transferred to the glove box without exposure to air. 4,4'-Di-*tert*-butyl-2,2'-dipyridyl (dtbpy, **L1**), zinc powder (Zn, >98% purity), manganese powder (Mn, 99.99% purity), and chlorotrimethylsilane (TMSCl, ≥98% purity) were purchased from Aldrich Chemical Co.. 4,4'-Dimethoxy-2,2'-dipyridyl (**L4**), iodotrimethylsilane (TMSI, ≥95% purity), and dicobalt octacarbonyl ($Co_2(CO)_8$, stabilized with 1-5% hexane) were purchased from TCI Chemicals. Nickel(II) chloride ethylene glycol dimethyl ether complex ($Ni(glyme)Cl_2$) was purchased from ABCR GmbH& Co. KG. The following known starting materials (aryl halides and nitroarenes) were prepared according to the literature procedures:[3-14]

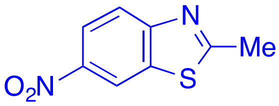

2-methyl-6-nitrobenzo[*d*]thiazole[3]

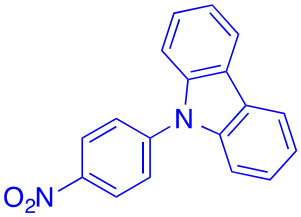

9-(4-nitrophenyl)-9*H*-carbazole[4]

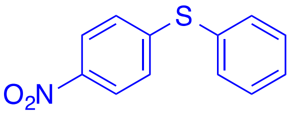

(4-nitrophenyl)(phenyl)sulfane[5]

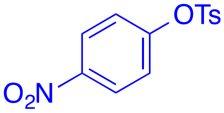

4-nitrophenyl 4-methylbenzenesulfonate[6]

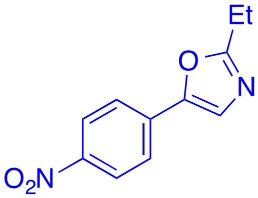

2-ethyl-5-(4-nitrophenyl)oxazole[7]

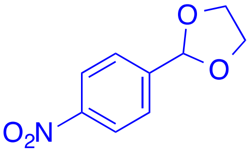

2-(4-nitrophenyl)-1,3-dioxolane[8]

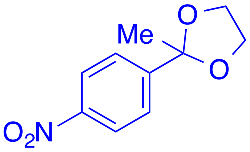

2-methyl-2-(4-nitrophenyl)-1,3-dioxolane[9]

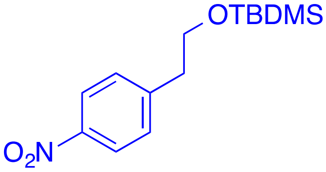

*tert*-butyldimethyl-(4-nitrophenethoxy)silane[10]

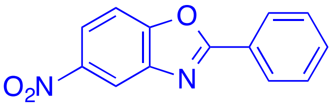

5-nitro-2-phenylbenzo[*d*]oxazole[11]

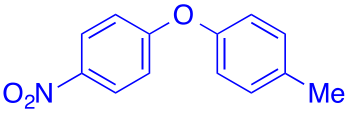

1-methyl-4-(4-nitrophenoxy)benzene[12]

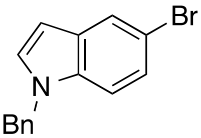

1-benzyl-5-bromo-1*H*-indole[13]

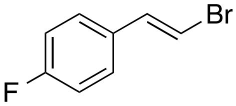

(*E*)-1-(2-bromovinyl)-4-fluorobenzene[14]

## (C) General Manipulation Considerations.

All manipulations for the nickel-catalyzed reductive aminocarbonylation with nitroarenes were set up in a 30 mL Teflon-screw capped test tubes under an inert nitrogen ($N_2$) atmosphere using glove-box techniques. The test tubes were then sealed with airtight electrical tapes and the reaction mixtures were stirred in a preheated oil-bath. Flash column chromatography was performed using silica gel (Silicycle, ultra pure grade). Preparative thin-layer chromatography (preparative TLC) was performed using preparative TLC plate (Merck Millipore, TLC Silica gel 60 $F_{254}$, 20 x 20 cm, catalogue number: 1.05715.0001) in a developing tank. Notably, the TLC plates used for the purification of amide products were washed with hexanes/triethylamine solution (volume ratio ~20:1) prior to use in order to minimize the product loss. The eluents for column chromatography and preparative TLC were presented as ratios of solvent volumes. Yields reported in the publication are of isolated materials unless otherwise noted. All new amide products were characterized by $^{1}$H and $^{13}$C NMR spectroscopies and high-resolution mass spectrometry (HRMS). All known amide products were characterized by $^{1}$H and $^{13}$C NMR spectroscopies and the spectra were compared with the reported data if provided.

# Supplementary Data

## (A) Optimization of Reaction Conditions for Nickel-Catalyzed Reductive Aminocarbonylation of Aryl Iodide with Nitroarene

Iodobenzene (**1a**) and 1-*tert*-butyl-4-nitrobenzene ($PhNO_2$, **2a**) were used as the model substrates of aryl iodide and nitroarene, respectively. After the screening of reaction parameters, we found that the reductive aminocarbonylation of **1a** with **2a** proceeded smoothly at 120 $^{o}$C in 16 h to yield the desired amide product, *N*-*tert*-butylphenyl benzamide (**3**), quantitatively in the presence of Zn reductant (2 equiv.), TMSCl additive (10 mol %), $Co_2(CO)_8$ (0.8 equiv.), DMF solvent (0.5 M of **1a**), and Ni(glyme)$Cl_2$ (10 mol %) / di-*tert*-butyl-2,2'-dipyridyl (**L1**) (10 mol %) as the catalyst system (Table S1, entry 1). The use of other ligands (Table S1, entries 2-7), nickel catalysts (Table S1, entries 8-10), reductant (Mn, Table S1, entry 12), solvents (Table S1, entries 14-16), and CO surrogates (Table S1, entries 17 and 18) led to diminishment in yields. The variation of the loadings of Ni(glyme)$Cl_2$ (Table S1, entry 11), Zn (Table S1, entry 13), $Co_2(CO)_8$ (Table S1, entry 19), and **2a** (Table S1, entry 20), as well as the change in DMF volume (Table S1, entry 16) or reaction temperature (Table S1, entry 21), also resulted in drop of yields. The use of other transition metal catalysts (Fe, Co, Cu. Mn) only resulted in very low yields of **3** (Table S1, entries 22-25). Control experiments demonstrated that Ni(glyme)$Cl_2$ catalyst and ligand **L1** were essential to promote the reductive aminocarbonylation (Table S1, entries 26 and 27). The reaction with aniline gave lower yield of product (63%, 77%, Table S1, entries 28 and 29).

**Table S1.** Optimizations of Nickel-Catalyzed Reductive Aminocarbonylation of Iodobenzene with 1-*tert*-Butyl-4-nitrobenzene

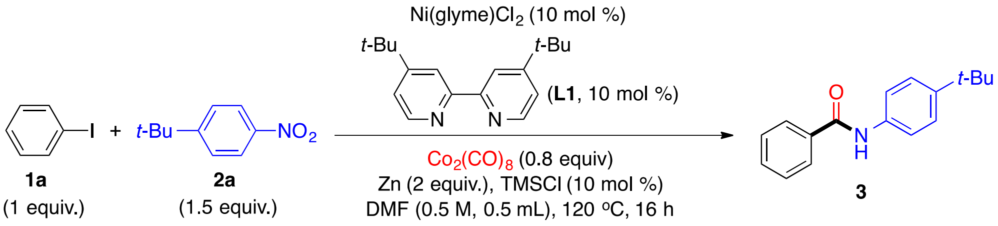


**1a** (1 equiv.) 0.25 mmol

**2a** (1.5 equiv.)

**3**

| Entry | Variations from 'standard conditions' | Yield of amide[a] |
|---|---|---|
| 1 | --- | 100 |
| 2 | 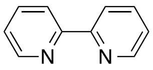 instead of **L1** | 92 |
| 3 | 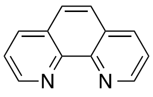 instead of **L1** | 91 |
| 4 | 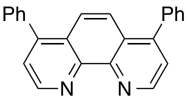 instead of **L1** | 91 |
| 5 | 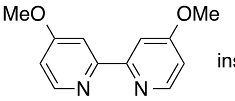 instead of **L1** | 87 |
| 6 | 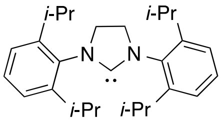 instead of **L1** | 44 |
| 7 | $Ni(PCy_3)_2Cl_2$ (10 mol %) instead of $Ni(glyme)Cl_2$ (10 mol %), **L1** (10 mol %) | 4 |
| 8 | $Ni(diglyme)Br_2$ (10 mol %) instead of $Ni(glyme)Cl_2$ | 99 |
| 9 | $NiCl_2$ (10 mol %) instead of $Ni(glyme)Cl_2$ | 92 |
| 10 | $NiI_2 \cdot xH_2O$ (10 mol %) instead of $Ni(glyme)Cl_2$ | 68 |
| 11 | $Ni(glyme)Cl_2$ (5 mol %), **L1** (5 mol %) instead of $Ni(glyme)Cl_2$ (10 mol %), **L1** (10 mol %) | 90 |
| 12 | Mn (2 equiv) instead of Zn (2 equiv) | 58 |
| 13 | Zn (1 equiv) instead of (2 equiv) | 71 |
| 14 | DMA instead of DMF | 94 |
| 15 | NMP instead of DMF | 85 |
| 16 | DMF (1 mL) instead of (0.5 mL) | 94 |
| 17 | $Fe(CO)_5$ (2 equiv) instead of $Co_2(CO)_8$ | 26 |
| 18 | $Mo(CO)_6$ (2 equiv) instead of $Co_2(CO)_8$ | 31 |
| 19 | $Co_2(CO)_8$ (0.6 equiv) instead of (0.8 equiv) | 79 |
| 20 | 1-*tert*-butyl-4-nitrobenzene (1.2 equiv) instead of (1.5 equiv) | 94 |
| 21 | 100 ºC instead of 120 ºC | 84 |
| 22 | $FeBr_2$ (10 mol %) instead of $Ni(glyme)Cl_2$ | 10 |
| 23 | $CoCl_2$ (10 mol %) instead of $Ni(glyme)Cl_2$ | < 5 |
| 24 | $CuBr_2$ (10 mol %) instead of $Ni(glyme)Cl_2$ | < 5 |
| 25 | $MnCl_2$ (10 mol %) instead of $Ni(glyme)Cl_2$ | < 5 |
| | **Control experiments:** | |
| 26 | No $Ni(glyme)Cl_2$ | < 5 |
| 27 | No **L1** | 55 |
| 28 | 1-*tert*-butylaniline (1.5 equiv) instead of 1-*tert*-butyl-4-nitrobenzene | 63 |
| 29 | 1-*tert*-butylaniline (1.5 equiv) instead of 1-*tert*-butyl-4-nitrobenzene; Zn (0.5 equiv) instead of (2 equiv) | 77 |

[a] Corrected GC yield using *n*-docecane as an internal standard.

## (B) Optimization of Reaction Conditions for Nickel-Catalyzed Reductive Aminocarbonylation of Aryl Bromide with Nitroarene

Bromobenzene (**1b**) and 1-*tert*-butyl-4-nitrobenzene ($PhNO_2$, **2a**) were used as the model substrates of aryl bromide and nitroarene, respectively. Initially, the optimal conditions for reductive aminocarbonylation of aryl iodide (Table S1, entry 1) were adopted in the reductive aminocarbonylation of aryl bromide. However, only a trace of desired product **3** was formed (Table S2, entry 1). After the screening of reaction parameters, we found that the reductive aminocarbonylation of **1b** with **2a** proceeded smoothly at 120 $^oC$ in 16 h to yield the desired amide product, *N*-*tert*-butylphenyl benzamide (**3**) in 87% yield in the presence of Mn reductant (5 equiv.), TMSI additive (1.5 equiv.), $Co_2(CO)_8$ (1.2 equiv.), DMF solvent (0.25 M of **1b**), and Ni(glyme)$Cl_2$ (10 mol %) / di-methoxy -2,2'-dipyridyl (**L4**) (10 mol %) as the catalyst system (Table S2, entry 2). The use of other ligands (Table S2, entries 3 and 4), nickel catalyst (Table S2, entry 5), reductant (Zn, Table S2, entry 7), additives (Table S2, entries 10-11), and solvents (Table S2, entry 14) led to diminishment in yields. The variation of the loadings of Ni(glyme)$Cl_2$ (Table S2, entry 6), Mn (Table S2, entries 8 and 9), TMSI (Entry S2, entries 12 and 13), and $Co_2(CO)_8$ (Table S2, entries 17 and 18), as well as the change in DMF volume (Table S1, entries 15 and 16), also resulted in drop of yields. Control experiments demonstrated that Ni(glyme)$Cl_2$ catalyst and ligand **L4** were essential to promote the reductive transmidation (Table S2, entries 19 and 20). The reaction with aniline only gave lower yield of product (15%, 39%, Table S2, entries 21 and 22).

**Table S2.** Optimizations of Nickel-Catalyzed Reductive Aminocarbonylation of Bromobenzene with 1-*tert*-Butyl-4-nitrobenzene

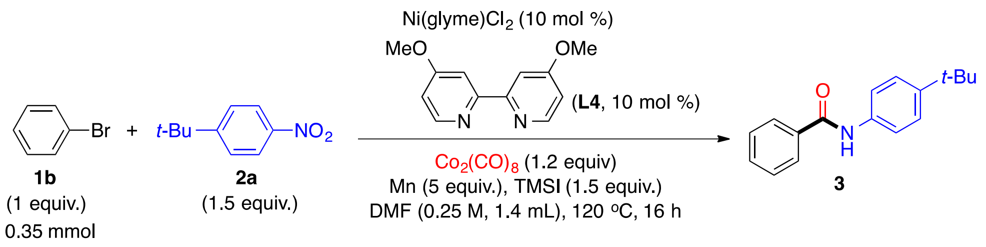


| Entry | Variations from 'standard conditions' | Yield of amide[a] |
|---|---|---|
| 1 | **Condition A** | 5 |
| 2 | --- | 87 |
| 3 | 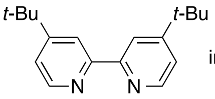  instead of **L4** | 65 |
| 4 | 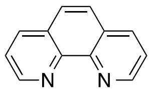  instead of **L4** | 51 |
| 5 | Ni(diglyme)$Br_2$ (10 mol %) instead of Ni(glyme)$Cl_2$ | 82 |
| 6 | Ni(glyme)$Cl_2$ (15 mol %), **L4** (15 mol %) instead of Ni(glyme)$Cl_2$ (10 mol %), **L4** (10 mol %) | 73 |
| 7 | Zn (5 equiv) instead of Mn (5 equiv) | 8 |
| 8 | Mn (4 equiv) instead of (5 equiv) | 76 |
| 9 | Mn (6 equiv) instead of (5 equiv) | 67 |
| 10 | TMSBr (1.5 equiv) instead of TMSI (1.5 equiv) | 27 |
| 11 | TMSCl (1.5 equiv) instead of TMSI (1.5 equiv) | 31 |
| 12 | TMSI (1 equiv) instead of TMSI (1.5 equiv) | 74 |
| 13 | TMSI (2 equiv) instead of TMSI (1.5 equiv) | 72 |
| 14 | DMA instead of DMF | 70 |
| 15 | DMF (1 mL) instead of (1.4 mL) | 71 |
| 16 | DMF (2 mL) instead of (1.4 mL) | 71 |
| 17 | $Co_2(CO)_8$ (1 equiv) instead of (1.2 equiv) | 65 |
| 18 | $Co_2(CO)_8$ (1.5 equiv) instead of (1.2 equiv) | 78 |
| **Control experiments:** | | |
| 19 | No Ni(glyme)$Cl_2$ | 36 |
| 20 | No **L4** | 69 |
| 21 | 1-*tert*-butylaniline (1.5 equiv) instead of 1-*tert*-butyl-4-nitrobenzene | 15 |
| 22 | 1-*tert*-butylaniline (1.5 equiv) instead of 1-*tert*-butyl-4-nitrobenzene; Mn (2 equiv) instead of (5 equiv); TMSI (10 mol%) instead of (5 equiv) | 39 |

[a] Corrected GC yield using *n*-docecane as an internal standard.

## (C) Replacing cobalt carbonyl with gaseous carbon monoxide

An oven-dried 5 mL schlenk flask equipped with a Teflon-coated magnetic stir bar was sequentially charged with zinc powder (Zn, 2 equiv., 0.70 mmo1, 46 mg), nickel(II) chloride ethylene glycol dimethyl ether complex (Ni(glyme)$Cl_2$, 10 mol %, 7.7 mg), di-*tert*-butyl-2,2'-dipyridyl (**L1**, 10 mol %, 9.4 mg) 4-(*tert*-butyl)-1-iodobenzene (1 equiv., 0.35 mmol, 91.0 mg), nitrobenzene (1.5 equiv., 0.525 mmol, 64.6 mg) naphthalene internal standard (0.3 equiv., 0.105 mmol, 13.5 mg) dimethylformamide solvent (DMF, 0.70 mL, 0.5 M with respect to amide), and chlorotrimethylsilane (TMSCl, 10 mol %, ~5 $\mu$L). The mixture was then freeze-pump-thawed through three cycles and back filled with the appropriate pressure of carbon monoxide. The mixture was then stirred for 16h in a preheated 120ºC oil bath. The reaction was then quenched with saturated ammonium chloride solution and neutralized with saturated sodium bicarbonate solution. The mixture was extracted with ethyl acetate and the yield estimated with GC.

| ***Pressure CO*** | ***GC yield*** |
|---|---|
| *1.4 bar* | 16% |
| *2.4 bar* | 6% |

**Table S3.** Reactivity of potential nitro-reduction intermediates with varying amounts of cobalt octacarbonyl.

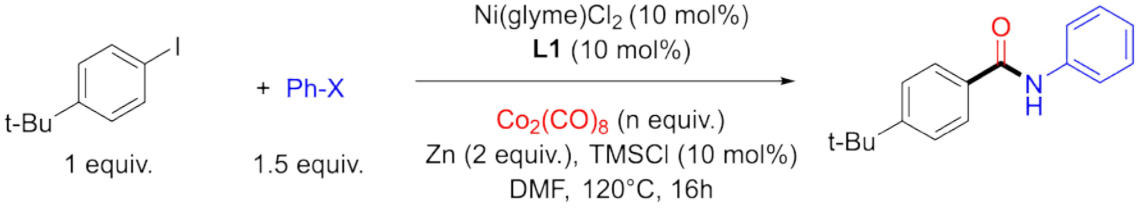


| | Ph-X | equiv. $Co_2(CO)_8$ | GC yield |
|---|---|---|---|
| 1/2 | Ph-N=N-Ph | 0.8 | 7% |
| 1/2 | Ph-N=N-Ph | 0.2 | 7% |
| | Ph-$NH_2$ | 0.8 | 32% |
| | Ph-$NH_2$ | 0.2 | 21% |
| | Ph-NCO | 0 | 5% |

## Experimental Section

**Synthesis of 2-Fluoro-3-(4-nitrophenyl)pyridine (S1).**

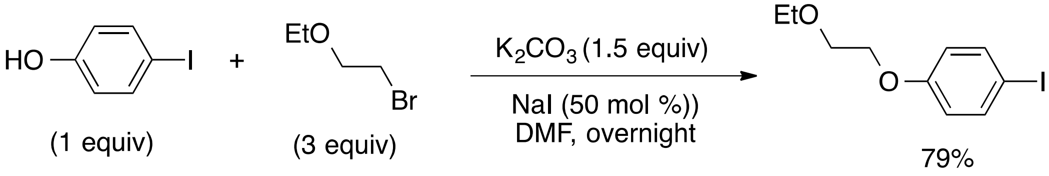


An oven-dried 500 mL round-bottom flask equipped with a Teflon-coated magnetic stir bar was charged with 1-iodophenol (1 equiv, 10 mmol, 2.2 g), 1-bromo-2-ethoxyethane (3 equiv, 30 mmol, 4.6 g), $K_2CO_3$ (1.5 equiv, 15 mmol, 2.1 g), NaI (50 mol %, 5 mmol, 750 mg), and DMF solvent (150 mL). The reaction mixture was then stirred at 120 $^{o}$C in a preheated oil bath overnight. After the reaction, the reaction mixture was washed with water (~300 mL x 2) and ethyl acetate (EtOAc, ~200 mL). The organic fraction was then washed with NaOH solution (~1 M, ~150 mL) followed by saturated NaCl solution (~200 mL). The organic fraction was further dried with $MgSO_4$ powder and concentrated *in vacuo* with the aid of a rotary evaporator. The residue was purified by flash chromatography with silica gel (without prior washing with $Et_3N$/hexanes) using a mixture of hexanes/EtOAc (8:1) as an eluent to afford the title compound (2.3 g, 79%). **$^{1}$H NMR** (400 MHz, $CDCl_3$): δ 7.52 (d, $J$ = 8.8 Hz, 2 H), 6.69 (d, $J$ = 8.5 Hz, 2 H), 4.06 (t, $J$ = 4.9 Hz, 2 H), 3.75 (t, $J$ = 4.9 Hz, 2 H), 3.58 (q, $J$ = 7.0 Hz, 2 H), 1.23 (t, $J$ = 7.0 Hz, 3 H). **$^{13}$C NMR** (100 MHz, $CDCl_3$): δ 158.8, 138.2, 117.1, 83.0, 68.8, 67.6, 66.9, 15.2. **HRMS** (ESI): Calcd for $C_{10}H_{13}IO_2$ [M]: 291.9955; Found: 291.9959.

**Synthesis of 2-Fluoro-3-(4-nitrophenyl)pyridine (S2).**

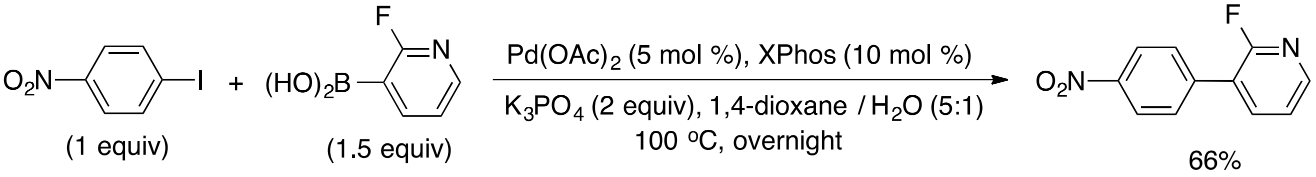


In a nitrogen-filled glovebox, an oven-dried 30 mL re-sealable screw-cap test tube equipped with a Teflon-coated magnetic stir bar was charged with 1-iodo-4-nitrobenzene (1 equiv, 4 mmol, 996 mg), (2-fluoropyridin-3-yl)boronic acid (1.5 equiv, 6 mmol, 846 mg), $Pd(OAc)_2$ (5 mol %, 45 mg), XPhos (10 mol%, 191 mg), $K_3PO_4$ (2 equiv, 8 mmol, 1.70 g), 1,4-dioxane solvent (10 mL), and deionized water (2 mL). The reaction mixture was then stirred at 100 $^{o}$C in a preheated oil bath overnight. After the reaction, the reaction mixture was washed with water (~100 mL) and ethyl acetate (EtOAc, ~50 mL). The aqueous fraction was further washed with EtOAc (2 x 20 mL). The combined organic fraction was dried with $MgSO_4$ powder and then concentrated *in vacuo* with the aid of a rotary evaporator. The residue was purified by flash chromatography with silica gel (without prior washing with $Et_3N$/hexanes) using a mixture of hexanes/EtOAc (5:1) as an eluent to afford the title compound (572 mg, 66%). **$^{1}$H NMR** (400 MHz, $CD_2Cl_2$): δ 8.34 (d, $J$ = 8.9 Hz, 2 H), 8.32-8.30 (m, 1 H), 7.96-7.91 (m, 1 H), 7.76 (dd, $J_{HH}$ = 8.8 Hz, $J_{HF}$ = 1.5 Hz, 2 H),7.38-7.35 (m, 1 H). **$^{13}$C NMR** (100 MHz, $CD_2Cl_2$): δ 160.3 (d, $J_{CF}$ = 239.6 Hz), 148.2 (d, $J_{CF}$ = 14.9 Hz), 147.9, 140.9 (d, $J_{CF}$ = 3.7 Hz), 140.8

(d, $J_{CF}$ = 5.2 Hz), 129.9 (d, $J_{CF}$ = 3.3 Hz), 124.1, 122.3 (d, $J_{CF}$ = 4.5 Hz), 121.9 (d, $J_{CF}$ = 27.9 Hz). **HRMS** (ESI): Calcd for $C_{11}H_8FN_2O_2$ [M+H]: 219.0564; Found: 219.0560.

**Amide Synthesis via Nickel-Catalyzed Reductive Aminocarbonylation of Aryl Iodide with Nitroarene (General Procedure A).** An oven-dried 30 mL re-sealable screw-cap test tube equipped with a Teflon-coated magnetic stir bar was sequentially charged with zinc powder (Zn, 2 equiv., 0.70 mmo1, 46 mg), aryl iodide (1 equiv., 0.35 mmol), nitroarene (1.5 equiv., 0.525 mmol), di-*tert*-butyl-2,2'-dipyridyl (**L1**, 10 mol %, 9.4 mg), nickel(II) chloride ethylene glycol dimethyl ether complex (Ni(glyme)$Cl_2$, 10 mol %, 7.7 mg), dicobalt octacarbonyl ($Co_2(CO)_8$, 0.8 equiv, 0.28 mmol, 101 mg), dimethylformaide solvent (DMF, 0.70 mL, 0.5 M with respect to amide), and chlorotrimethylsilane (TMSCl, 10 mol %, ~5 $\mu$L). The resulting mixture was stirred at a preheated oil bath for 16 h. After the reaction, the reaction mixture was cooled down to room temperature, and it was acidified with saturated $NH_4Cl$ solution (~5 mL) and then neutralized with saturated $NaHCO_3$ solution (~10 mL). The crude product in the aqueous fraction was extracted with ethyl acetate (EtOAc, ~20 mL). The aqueous fraction was further washed with EtOAc (3 x ~10 mL). The combined organic fractions were concentrated *in vacuo* with the aid of a rotary evaporator. The residue was purified by preparative thin-layer chromatography (TLC) using a solvent mixture (hexanes, EtOAc, $CH_2Cl_2$) as an eluent to afford the purified amide product.

**Amide Synthesis via Nickel-Catalyzed Reductive Aminocarbonylation of Aryl Bromide with Nitroarene (General Procedure B).** An oven-dried 30 mL re-sealable screw-cap test tube equipped with a Teflon-coated magnetic stir bar was sequentially charged with manganese powder (Mn, 5 equiv., 1.75 mmo1, 96 mg), aryl bromide (1 equiv., 0.35 mmol), nitroarene (1.5 equiv., 0.525 mmol), di-methoxy-2,2'-dipyridyl (**L4**, 10 mol %, 7.6 mg), nickel(II) chloride ethylene glycol dimethyl ether complex (Ni(glyme)$Cl_2$, 10 mol %, 7.7 mg), dicobalt octacarbonyl ($Co_2(CO)_8$, 1.2 equiv, 0.42 mmol, 151 mg), dimethylformaide solvent (DMF, 1.4 mL, 0.25 M with respect to amide), and iodotrimethylsilane (TMSI, 1.5 equiv., 0.525 mmol, 75 $\mu$L). The resulting mixture was stirred at a preheated oil bath for 16 h. After the reaction, the reaction mixture was cooled down to room temperature, and it was acidified with saturated $NH_4Cl$ solution (~5 mL) and then neutralized with saturated $NaHCO_3$ solution (~10 mL). The crude product in the aqueous fraction was extracted with ethyl acetate (EtOAc, ~20 mL). The aqueous fraction was further washed with EtOAc (3 x ~10 mL). The combined organic fractions were concentrated *in vacuo* with the aid of a rotary evaporator. The residue was purified by preparative thin-layer chromatography (TLC) using a solvent mixture (hexanes, EtOAc, $CH_2Cl_2$) as an eluent to afford the purified amide product.

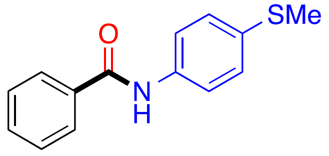

***N*-(4-(Methylthio)phenyl)benzamide (3a).**[15] Following the general procedure A, the title compound was prepared using iodobenzene (39 uL) and 1-nitro-4-thiomethylbenzene (89 mg). The crude product was purified by preparative TLC using hexanes/EtOAc/$CH_2Cl_2$ (75:20:5) as an eluent to afford the title compound as a deep brown amorphous solid (62 mg, 73%). **$^1$H NMR** (400 MHz, $CD_2Cl_2$): δ 7.90 (s, 1 H), 7.86 (d, $J$ = 7.2 Hz, 2 H), 7.61-7.55 (ovrlp, 3 H), 7.50 (t, $J$ = 7.2 Hz, 2 H), 7.28 (d, $J$ = 8.2 Hz, 2 H), 2.49 (s, 3 H). **$^{13}$C NMR** (100 MHz, $CD_2Cl_2$): δ 165.8, 136.2, 135.4, 134.4, 132.2, 129.2, 128.2, 127.4, 121.2, 16.8.

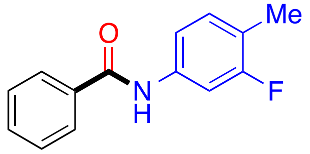


***N*-(3-Fluoro-4-methylphenyl)benzamide (3b).** Following the general procedure A, the title compound was prepared using iodobenzene (39 uL) and 2-fluoro-4-nitrotoluene (82 mg). The crude product was purified by preparative TLC using hexanes/EtOAc/$CH_2Cl_2$ (85:10:5) as an eluent to afford the title compound as a pale brown amorphous solid (53 mg, 66%). **$^1$H NMR** (400 MHz, $CD_2Cl_2$): δ 7.98 (s, 1 H), 7.85 (d, $J$ = 7.4 Hz, 2 H), 7.59-7.55 (ovrlp, 2 H), 7.49 (t, $J$ = 7.2 Hz, 2 H), 7.20-7.14 (ovrlp, 2 H), 2.25 (s, 3 H). **$^{13}$C NMR** (100 MHz, $CD_2Cl_2$): δ 165.9, 161.6 (d, $^1J_{CF}$ = 241.4 Hz), 137.8 (d, $^3J_{CF}$ = 10.7 Hz), 135.3, 132.3, 131.8 (d, $^3J_{CF}$ = 6.4 Hz), 129.2, 127.4, 121.1 (d, $^2J_{CF}$ = 17.4 Hz), 115.8 (d, $^4J_{CF}$ = 3.4 Hz), 107.8 (d, $^2J_{CF}$ = 27.3 Hz), 14.2 (d, $^3J_{CF}$ = 3.3 Hz). **HRMS** (ESI): Calcd for $C_{14}H_{13}FNO$ [M+H]: 230.0981; Found: 230.0984.

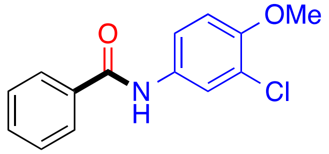


***N*-(3-Chloro-4-methoxyphenyl)benzamide (3c).** Following the general procedure A, the title compound was prepared using iodobenzene (39 uL) and 2-chloro-4-nitroanisole (98 mg). The crude product was purified by preparative TLC using hexanes/EtOAc/$CH_2Cl_2$ (75:20:5) as an eluent to afford the title compound as a brown amorphous solid (69 mg, 75%). **$^1$H NMR** (400 MHz, $CD_2Cl_2$): δ 8.00 (s, 1 H), 7.84 (d, $J$ = 7.0 Hz, 2 H), 7.73 (s, 1 H), 7.55 (d, $J$ = 6.9 Hz, 1 H), 7.50-7.44 (ovrlp, 3 H), 6.92 (d, $J$ = 8.8 Hz, 1 H), 3.88 (s, 3 H). **$^{13}$C NMR** (100 MHz, $CD_2Cl_2$): δ 166.0, 152.5, 135.1, 132.2, 132.1, 129.1, 127.4, 123.2, 122.7, 120.6, 112.7, 56.7. **HRMS** (ESI): Calcd for $C_{14}H_{13}ClNO_2$ [M+H]: 262.0645; Found: 262.0641.

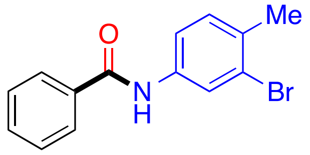

***N*-(3-Bromo-4-methylphenyl)benzamide (3d).** Following the general procedure A, the title compound was prepared using iodobenzene (39 uL) and 2-bromo-4-nitrotoluene (113 mg). The crude product was purified by preparative TLC using hexanes/EtOAc/$CH_2Cl_2$ (85:10:5) as an eluent to afford the title compound as a deep brown amorphous solid (68 mg, 67%). **$^1$H NMR** (400 MHz, $CD_2Cl_2$): δ 8.15 (s, 1 H), 7.93 (d, $J$ = 1.5 Hz, 1 H), 7.84 (d, $J$ = 7.5 Hz, 2 H), 7.54 (d, $J$ = 1.8 Hz, 1 H), 7.47-7.44 (ovrlp, 3 H), 7.20 (d, $J$ = 8.2 Hz, 1 H), 2.37 (s, 3 H). **$^{13}$C NMR** (100 MHz, $CD_2Cl_2$): δ 166.1, 137.4, 135.1, 134.3, 132.3, 131.1, 129.1, 127.4, 125.0, 124.4, 119.8, 22.4. **HRMS** (ESI): Calcd for $C_{14}H_{13}BrNO$ [M+H]: 290.0189; Found: 290.0181.

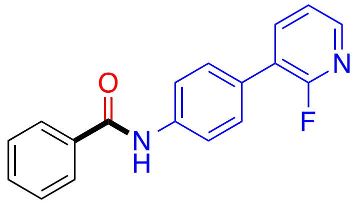


***N*-(4-(2-Fluoropyridin-3-yl)phenyl)benzamide (3e).** Following the general procedure A, the title compound was prepared using iodobenzene (39 uL) and 2-fluoro-3-(4-nitrophenyl)pyridine (114 mg). The crude product was purified by preparative TLC using hexanes/EtOAc/$CH_2Cl_2$ (65:30:5) as an eluent to afford the title compound as a brown amorphous solid (68 mg, 66%). **$^1$H NMR** (400 MHz, $CD_2Cl_2$): δ 8.17 (s, 1 H), 8.01 (s, 1 H), 7.94-7.87 (ovrlp, 3 H), 7.79 (d, $J$ = 7.6 Hz, 2 H), 7.62-7.58 (ovrlp, 3 H), 7.53 (t, $J$ = 6.4 Hz, 2 H), 7.31 (t, $J$ = 6.5 Hz, 1 H). **$^{13}$C NMR** (100 MHz, DMSO-$d_6$): δ 165.7, 159.6 (d, $J_{CF}$ = 235.6 Hz), 146.0 (d, $J_{CF}$ = 14.8 Hz), 141.0 (d, $J_{CF}$ = 4.2 Hz), 139.5, 134.8, 131.7, 129.0 (d, $J_{CF}$ = 3.0 Hz), 128.4, 127.7, 122.60 (d, $J_{CF}$ = 27.8 Hz), 122.65 (d, $J_{CF}$ = 4.1 Hz), 120.4. **HRMS** (ESI): Calcd for $C_{18}H_{14}FN_2O$ [M+H]: 293.1090; Found: 293.1104.

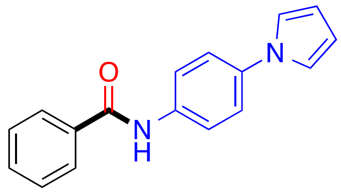


***N*-(4-(1*H*-Pyrrol-1-yl)phenyl)benzamide (3f).** Following the general procedure A, the title compound was prepared using iodobenzene (39 uL) and 1-(4-nitrophenyl)-1*H*-pyrrole (99 mg). The crude product was purified by preparative TLC using hexanes/EtOAc/$CH_2Cl_2$ (85:10:5) as an eluent to afford the title compound as a reddish-brown amorphous solid (39 mg, 42%). **$^1$H NMR** (400 MHz, DMSO-$d_6$): δ 10.34 (s, 1 H), 7.98 (d, $J$ = 6.7 Hz, 2 H), 7.88 (d, $J$ = 8.0 Hz, 2 H), 7.62-7.51 (ovrlp, 5 H), 7.34 (s, 2 H), 6.26 (s, 2 H). **$^{13}$C NMR** (100 MHz, DMSO-$d_6$): δ 165.4, 136.5, 135.7, 134.8, 131.6, 128.4, 127.6, 121.3, 119.5, 118.8, 110.2. **HRMS** (ESI): Calcd for $C_{17}H_{15}N_2O$ [M+H]: 263.1184; Found: 263.1190.

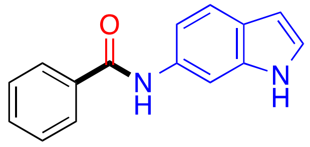

***N*-(1*H*-Indol-6-yl)benzamide (3g).** Following the general procedure A, the title compound was prepared using iodobenzene (39 uL) and 6-nitro-1*H*-indole (85 mg), Ni(glyme)$Cl_2$ (15 mol %, 11.6 mg), **L1** (15 mol %, 14.1 mg), $Co_2(CO)_8$ (1 equiv, 120 mg), and DMF (1.4 mL). The crude product was purified by preparative TLC using hexanes/EtOAc/$CH_2Cl_2$ (85:10:5) as an eluent to afford the title compound as a brown amorphous solid (39 mg, 47%). **$^1$H NMR** (400 MHz, DMSO-$d_6$): δ 11.06 (s, 1 H), 10.19 (s, 1 H), 8.09 (s, 1 H), 7.98 (d, $J$ = 7.0 Hz, 2 H), 7.60-7.51 (ovrlp, 3 H), 7.48 (d, $J$ = 8.6 Hz, 1 H), 7.31-7.28 (ovrlp, 2 H), 6.38 (s, 1 H). **$^{13}$C NMR** (400 MHz, DMSO-$d_6$): δ 165.2, 135.8, 135.4, 133.2, 131.2, 128.3, 127.6, 125.1, 124.2, 119.6, 113.4, 103.3, 100.9. **HRMS** (ESI): Calcd for $C_{15}H_{13}N_2O$ [M+H]: 237.1028; Found: 237.1029.

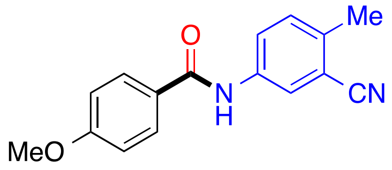

***N*-(3-Cyano-4-methylphenyl)-4-methoxybenzamide (3h).** Following the general procedure A, the title compound was prepared using 4-iodoanisole (82 mg), 2-cyano-4-nitrotoluene (85 mg), and $Co_2(CO)_8$ (1 equiv, 120 mg). The crude product was purified by preparative TLC using hexanes/EtOAc/$CH_2Cl_2$ (65:30:5) as an eluent to afford the title compound as brown amorphous solid (51 mg, 55%). **$^1$H NMR** (400 MHz, $CDCl_3$): δ 8.03 (s, 1 H), 7.92 (d, $J$ = 1.6 Hz, 1 H), 7.83 (d, $J$ = 8.7 Hz, 2 H), 7.73 (dd, $J$ = 8.3 Hz, $J$ = 1.6 Hz, 1 H), 7.27 (d, $J$ = 7.0 Hz, 1 H), 6.95 (d, $J$ = 8.7 Hz, 2 H), 3.86 (s, 3 H), 2.50 (s, 3 H). **$^{13}$C NMR** (100 MHz, $CDCl_3$): δ 165.5, 162.9, 137.7, 136.6, 131.0, 129.2, 126.4, 124.8, 123.8, 118.0, 114.2, 113.2, 55.6, 20.0. **HRMS** (ESI): Calcd for $C_{16}H_{15}N_2O_2$ [M+H]: 267.1133; Found: 267.1142.

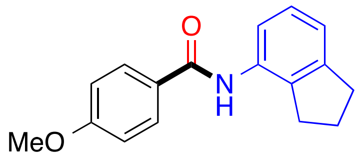

***N*-(2,3-Dihydro-1*H*-inden-4-yl)-4-methoxybenzamide (3i).**[16] Following the general procedure A, the title compound was prepared using 4-iodoanisole (82 mg) and 4-nitro-2,3-dihydro-1*H*-indene (86 mg). The crude product was purified by preparative TLC using hexanes/EtOAc/$CH_2Cl_2$ (85:10:5) as an eluent to afford the title compound as a pale brown amorphous solid (85 mg, 91%). **$^1$H NMR** (400 MHz, $CDCl_3$): δ 7.85-7.81 (ovrlp, 3 H), 7.64 (s, 1 H), 7.18 (t, $J$ = 7.6 Hz, 1 H), 7.04 (d, $J$ = 7.2 Hz, 1 H), 6.96 (d, $J$ = 8.4 Hz, 2 H), 3.85 (s, 3 H), 2.96 (t, $J$ = 7.2 Hz, 2 H), 2.87 (t, $J$ = 7.2 Hz, 2 H), 2.12 (qu, $J$ = Hz, 2 H). **$^{13}$C NMR** (100 MHz, $CDCl_3$): δ 165.1, 162.5, 145.4, 134.4, 134.3, 129.0, 127.4, 127.3, 120.9, 119.0, 114.1, 55.6, 33.3, 30.2, 24.9.

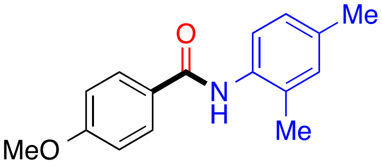

***N*-(2,4-Dimethylphenyl)-4-methoxybenzamide (3j).** Following the general procedure A, the title compound was prepared using 4-iodoanisole (82 mg) and 2,4-dimethyl-nitrobenzene (79 mg). The crude product was purified by preparative TLC using hexanes/EtOAc/$CH_2Cl_2$ (85:10:5) as an eluent to afford the title compound as an off-white amorphous solid (76 mg, 85%). **$^1$H NMR** (400 MHz, $CD_2Cl_2$): δ 7.84 (d, *J* = 8.8 Hz, 2 H), 7.66-7.60 (ovrlp, 2 H), 7.06 (s, 1 H), 7.04 (d, *J* = 8.2 Hz, 1 H), 6.98 (d, *J* = 8.8 Hz, 2 H), 3.87 (s, 3 H), 2.32 (s, 3 H), 2.27 (s, 3 H). **$^{13}$C NMR** (100 MHz, $CD_2Cl_2$): δ 165.4, 162.8, 135.5, 133.9, 131.5, 130.8, 129.3, 127.6, 127.5, 124.1, 114.2, 55.9, 21.0, 18.0. **HRMS** (ESI): Calcd for $C_{16}H_{18}NO_2$ [M+H]: 256.1338; Found: 256.1353.

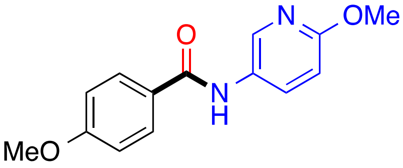

**4-Methoxy-*N*-(6-methoxypyridin-3-yl)benzamide (3k).** Following the general procedure A, the title compound was prepared using 4-iodoanisole (82 mg), 2-methoxy-5-nitropyridine (1.5 equiv., 81 mg), Ni(glyme)$Cl_2$ (15 mol %, 11.6 mg), **L1** (15 mol %, 14.1 mg), $Co_2(CO)_8$ (1 equiv, 120 mg), and DMF (1.4 mL). The crude product was purified by preparative TLC using hexanes/EtOAc/$CH_2Cl_2$ (65:30:5) as an eluent to afford the title compound as a brown amorphous solid (47 mg, 52%). **$^1$H NMR** (400 MHz, $CDCl_3$): δ 8.25 (s, 1 H), 8.11 (s, 1 H), 7.97 (d, *J* = 7.8 Hz, 1 H), 7.83 (d, *J* = 7.8 Hz, 2 H), 6.91 (d, *J* = 7.7 Hz, 2 H), 6.71 (d, *J* = 8.4 Hz, 1 H), 3.91 (s, 3 H), 3.84 (s, 3 H). **$^{13}$C NMR** (100 MHz, $CDCl_3$): δ 165.8, 162.7, 161.2, 139.2, 133.0, 129.2, 128.9, 126.6, 114.1, 110.7, 55.6, 53.7. **HRMS** (ESI): Calcd for $C_{14}H_{15}N_2O_3$ [M+H]: 259.1083; Found: 259.1087.

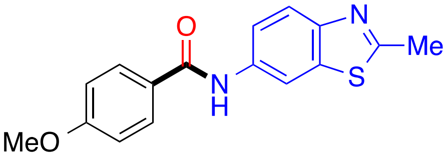

**4-Methoxy-*N*-(2-methylbenzo[*d*]thiazol-6-yl)benzamide (3l).** Following the general procedure A, the title compound was prepared using 4-iodoanisole (82 mg) and 2-methyl-6-nitrobenzo[*d*]thiazole (102 mg). The crude product was purified by preparative TLC using hexanes/EtOAc/$CH_2Cl_2$ (85:10:5) as an eluent to afford the title compound as a reddish-brown amorphous solid (66 mg, 63%). **$^1$H NMR** (400 MHz, DMSO-$d_6$): δ 10.28 (s, 1 H), 8.53 (d, *J* = 1.1 Hz, 1 H), 7.98 (d, *J* = 8.7 Hz, 2 H), 7.86 (d, *J* = 8.7 Hz, 1 H), 7.74 (dd, *J* = 8.7 Hz, *J* = 1.5 Hz, 1 H), 7.08 (d, *J* = 8.6 Hz, 2 H), 3.85 (s, 3 H), 2.77 (s, 3 H). **$^{13}$C NMR** (100 MHz, DMSO-$d_6$): δ 165.5, 165.0, 162.0, 149.2, 136.4, 135.6, 129.6, 126.8, 121.6,

119.6, 113.6, 112.6, 55.4, 19.7. **HRMS** (ESI): Calcd for $C_{16}H_{15}N_2O_2S$ [M+H]: 299.0854; Found: 299.0851.

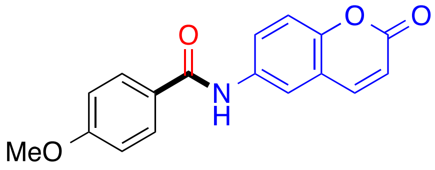


**4-Methoxy-*N*-(2-oxo-2*H*-chromen-6-yl)benzamide (3m).** Following the general procedure A, the title compound was prepared using 4-iodoanisole (82 mg), 6-nitro-2*H*-chromen-2-one (1.5 equiv., 100 mg), Ni(glyme)$Cl_2$ (15 mol %, 11.6 mg), **L1** (15 mol %, 14.1 mg), $Co_2(CO)_8$ (1 equiv, 120 mg), and DMF (1.4 mL). The crude product was purified by preparative TLC using hexanes/EtOAc/$CH_2Cl_2$ (75:20:5) as an eluent to afford the title compound as a pale brown amorphous solid (45 mg, 43%). **$^1$H NMR** (400 MHz, DMSO-$d_6$): δ 10.30 (s, 1 H), 8.21 (s, 1 H), 8.09-7.87 (ovrlp, 4 H), 7.40 (d, $J$ = 5.9 Hz, 1 H), 7.07 (s, 2 H), 6.49 (d, $J$ = 7.7 Hz, 1 H), 3.84 (s, 3 H). **$^{13}$C NMR** (100 MHz, DMSO-$d_6$): δ 164.9, 162.0, 160.0, 149.5, 144.4, 135.7, 129.6, 126.5, 124.5, 119.0, 118.6, 116.4, 113.6, 55.4. **HRMS** (ESI): Calcd for $C_{17}H_{14}NO_4$ [M+H]: 296.0923; Found: 296.0922.

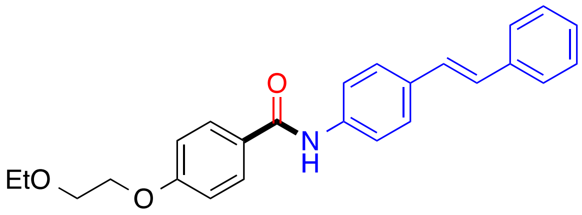


**(*E*)-4-(2-ethoxyethoxy)-*N*-(4-styrylphenyl)benzamide (3n).** Following the general procedure A, the title compound was prepared using 1-(2-ethoxyethoxy)-4-iodobenzene (102 mg), (*E*)-1-nitro-4-styrylbenzene (118 mg), Ni(glyme)$Cl_2$ (15 mol %, 11.6 mg), **L1** (15 mol %, 14.1 mg), $Co_2(CO)_8$ (1 equiv, 120 mg), and DMF (1.4 mL). The crude product was purified by preparative TLC using hexanes/EtOAc/$CH_2Cl_2$ (75:20:5) as an eluent to afford the title compound as a brown amorphous solid (84 mg, 62%). **$^1$H NMR** (400 MHz, DMSO-$d_6$): δ 10.17 (s, 1 H), 7.97 (d, $J$ = 8.5 Hz, 2 H), 7.82 (d, $J$ = 8.3 Hz, 2 H), 7.62-7.54 (ovrlp, 4 H), 7.37 (t, $J$ = 7.4 Hz, 2 H), 7.28-7.18 (ovrlp, 3 H), 7.08 (d, $J$ = 8.5 Hz, 2 H), 4.17 (t, $J$ = 4.2 Hz, 2 H), 3.72 (t, $J$ = 4.0 Hz, 2 H), 3.51 (q, $J$ = 6.9 Hz, 2 H), 1.13 (t, $J$ = 7.0 Hz, 3 H). **$^{13}$C NMR** (100 MHz, DMSO-$d_6$): δ 164.8, 161.2, 138.9, 137.2, 132.1, 129.6, 128.6, 128.0, 127.3, 127.0, 126.9, 126.7, 126.2, 120.3, 114.0, 68.2, 67.4, 65.7, 15.1. **HRMS** (ESI): Calcd for $C_{25}H_{26}NO_3$ [M+H]: 388.1913; Found: 388.1911.

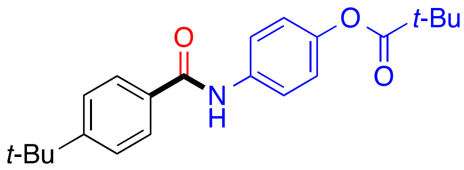

**4-(4-(*tert*-Butyl)benzamido)phenyl pivalate (3o).** Following the general procedure A, the title compound was prepared using 1-*tert*-butyl-4-iodobenzene (91 mg), 4-nitrophenyl pivalate (117 mg), Ni(glyme)$Cl_2$ (15 mol %, 11.6 mg), **L1** (15 mol %, 14.1 mg), $Co_2(CO)_8$ (1 equiv, 120 mg), and DMF (1.4 mL). The crude product was purified by preparative TLC using hexanes/EtOAc/$CH_2Cl_2$ (85:10:5) as an eluent to afford the title compound as a white amorphous solid (74 mg, 60%). **$^1$H NMR** (400 MHz, $CDCl_3$): δ 8.07 (s, 1 H), 7.79 (d, *J* = 8.4 Hz, 2 H), 7.61 (d, *J* = 8.8 Hz, 2 H), 7.45 (d, *J* = 8.4 Hz, 2 H), 6.99 (d, *J* = 8.8 Hz, 2 H), 1.35 (s, 9 H), 1.34 (s, 9 H). **$^{13}$C NMR** (100 MHz, $CDCl_3$): δ 177.5, 165.9, 155.4, 147.4, 135.8, 132.0, 127.1, 125.7, 121.9, 121.3, 39.2, 35.1, 31.3, 27.3. **HRMS** (ESI): Calcd for $C_{22}H_{28}NO_3$ [M+H]: 354.2069; Found: 354.2079.

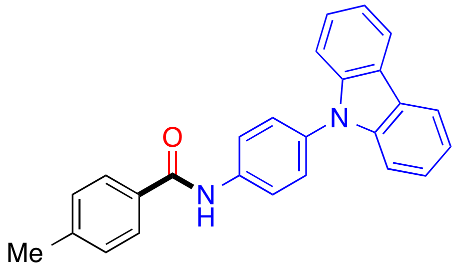


***N*-(4-(9*H*-Carbazol-9-yl)phenyl)-4-methylbenzamide (3p).**[16] Following the general procedure A, the title compound was prepared using 4-iodotoluene (76 mg), 9-(4-nitrophenyl)-9*H*-carbazole (151 mg), and DMF (1.4 mL). The crude product was purified by preparative TLC using hexanes/EtOAc/$CH_2Cl_2$ (85:10:5) as an eluent to afford the title compound as a brown amorphous solid (82 mg, 62%). **$^1$H NMR** (400 MHz, $CDCl_3$): δ 8.14 (d, *J* = 7.8 Hz, 2 H), 7.98 (s, 1 H), 7.88 (d, *J* = 8.8 Hz, 2 H), 7.83 (d, *J* = 8.2 Hz, 2 H), 7.55 (d, *J* = 8.7 Hz, 2 H), 7.43-7.38 (ovrlp, 4 H), 7.32 (d, *J* = 8.2 Hz, 2 H), 7.30-7.26 (m, 2 H), 2.45 (s, 3 H). **$^{13}$C NMR** (100 MHz, $CDCl_3$): δ 166.1, 142.8, 141.1, 137.4, 133.8, 131.9, 129.6, 127.9, 127.3, 126.1, 123.4, 121.7, 120.4, 120.0, 109.8, 21.6.

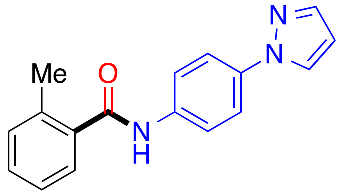


***N*-(4-(1*H*-Pyrazol-1-yl)phenyl)-2-methylbenzamide (3q).** Following the general procedure A, the title compound was prepared using 2-iodotoluene (76 mg), 1-(4-nitrophenyl)-1*H*-pyrazole (99 mg), Ni(glyme)$Cl_2$ (15 mol %, 11.6 mg), **L1** (15 mol %, 14.1 mg), and $Co_2(CO)_8$ (1 equiv, 120 mg). The crude product was purified by preparative TLC using hexanes/EtOAc/$CH_2Cl_2$ (75:20:5) as an eluent to afford the title compound as a brown amorphous solid (41 mg, 42%). **$^1$H NMR** (400 MHz, $CDCl_3$): δ 7.89 (s, 1 H), 7.77 (s, 1 H), 7.73-7.60 (ovrlp, 5 H), 7.47 (d, *J* = 6.6 Hz, 1 H), 7.36 (t, *J* = 6.7 Hz, 1 H), 7.30-7.21 (ovrlp, 2 H), 6.46 (s, 1 H), 2.50 (s, 3 H). **$^{13}$C NMR** (100 MHz, $CDCl_3$): δ 168.2, 141.1, 136.8, 136.64, 136.57, 136.2, 131.4, 130.5, 126.9, 126.8, 126.0, 120.9, 120.0, 107.7, 20.0. **HRMS** (ESI): Calcd for $C_{17}H_{16}N_3O$ [M+H]: 278.1293; Found: 278.1293.

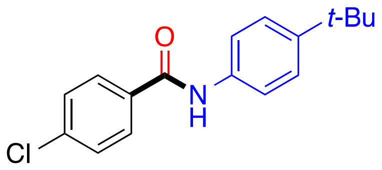

***N*-(4-(*tert*-Butyl)phenyl)-4-chlorobenzamide (3r).** Following the general procedure A, the title compound was prepared using 1-chloro-4-iodobenzene (84 mg) and 1-(*tert*-butyl)-4-nitrobenzene (95 mg). The crude product was purified by preparative TLC using hexanes/EtOAc/$CH_2Cl_2$ (85:10:5) as an eluent to afford the title compound as a white amorphous solid (55 mg, 55%). $^{1}$**H NMR** (400 MHz, $CDCl_3$): δ 7.88 (s, 1 H), 7.78 (d, $J$ = 8.4 Hz, 2 H), 7.53 (d, $J$ = 8.5 Hz, 2 H), 7.41 (d, $J$ = 8.4 Hz, 2 H), 7.37 (d, $J$ = 8.6 Hz, 2 H), 1.32 (s, 9 H). $^{13}$**C NMR** (100 MHz, $CDCl_3$): δ 164.8, 148.0, 138.1, 135.2, 133.6, 129.1, 128.6, 126.1, 120.3, 34.6, 31.5. **HRMS** (ESI): Calcd for $C_{17}H_{19}ClNO$ [M+H]: 288.1155; Found: 288.1154.

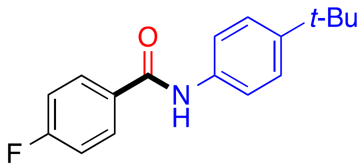

***N*-(4-(*tert*-Butyl)phenyl)-4-fluorobenzamide (3s).[16]** Following the general procedure A, the title compound was prepared using 1-fluoro-4-iodobenzene (78 mg) and 1-(*tert*-butyl)-4-nitrobenzene (94 mg). The crude product was purified by preparative TLC using hexanes/EtOAc/$CH_2Cl_2$ (85:10:5) as an eluent to afford the title compound as a brown amorphous solid (56 mg, 60%). $^{1}$**H NMR** (400 MHz, $CDCl_3$): δ 7.95 (s, 1 H), 7.85 (dd, $^{3}J_{HH}$ = 8.1 Hz, $^{4}J_{CF}$ = 5.6 Hz, 2 H), 7.53 (d, $J$ = 8.4 Hz, 2 H), 7.36 (d, $J$ = 8.4 Hz, 2 H), 7.10 (dd, $^{3}J_{HH}$ = 8.1 Hz, $^{3}J_{CF}$ = 8.1 Hz, 2 H), 1.32 (s, 9 H). $^{13}$**C NMR** (100 MHz, $CDCl_3$): δ 164.93 (d, $^{1}J_{CF}$ = 250.9 Hz), 164.91, 147.9, 135.3, 131.3 (d, $^{4}J_{CF}$ = 3.1 Hz), 129.6 (d, $^{3}J_{CF}$ = 8.9 Hz), 126.0, 120.4, 115.9 (d, $^{2}J_{CF}$ = 21.8 Hz), 34.6, 31.5.

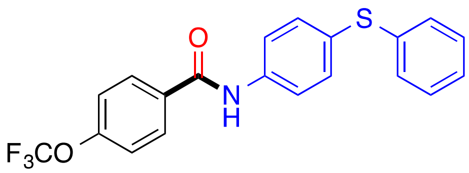

***N*-(4-(Phenylthio)phenyl)-4-(trifluoromethoxy)benzamide (3t).** Following the general procedure A, the title compound was prepared using 1-iodo-4-(trifluoromethoxy)benzene (101 mg) and (4-nitrophenyl)(phenyl)sulfane (121 mg) and DMF (1.4 mL). The crude product was purified by preparative TLC using hexanes/EtOAc/$CH_2Cl_2$ (85:10:5) as an eluent to afford the title compound as a brown amorphous solid (56 mg, 41%). $^{1}$**H NMR** (400 MHz, $CDCl_3$): δ 7.98 (s, 1 H), 7.88 (d, $J$ = 8.2 Hz, 2 H), 7.58 (d, $J$ = 8.0 Hz, 2 H), 7.37 (d, $J$ = 8.0 Hz, 2 H), 7.33-7.19 (ovrlp, 7 H). $^{13}$**C NMR** (100 MHz, $CD_2Cl_2$): δ 164.7, 152.1, 137.9, 137.0, 133.8, 133.2, 131.2, 130.6, 129.6, 129.5, 127.3, 121.5, 121.3, 120.8 (1, $^{1}J_{CF}$ = 256.7 Hz). **HRMS** (ESI): Calcd for $C_{20}H_{14}F_3NO_2S$ [M+H]: 390.0775; Found: 390.0787.

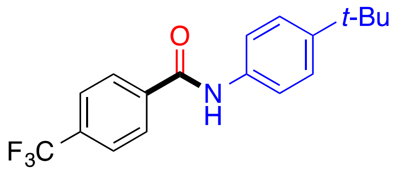


***N*-(4-(*tert*-Butyl)phenyl)-4-(trifluoromethyl)benzamide (3u).**[16] Following the general procedure A, the title compound was prepared using 4-iodobenzotrifluoride (95 mg) and 1-(*tert*-butyl)-4-nitrobenzene (94 mg). The crude product was purified by preparative TLC using hexanes/EtOAc/$CH_2Cl_2$ (85:10:5) as an eluent to afford the title compound as a brown amorphous solid (62 mg, 55%). **$^1$H NMR** (400 MHz, $CDCl_3$): δ 8.69 (s, 1 H), 7.85 (d, $J$ = 7.9 Hz, 2 H), 7.55-7.52 (ovrlp, 4 H), 7.31 (d, $J$ = 8.4 Hz, 2 H), 1.30 (s, 9 H). **$^{13}$C NMR** (100 MHz, $CDCl_3$): δ 165.2, 148.2, 138.3, 135.1, 133.3 (q, $^2J_{CF}$ = 32.5 Hz), 127.8, 125.9, 125.6 (q, $^3J_{CF}$ = 3.7 Hz), 123.7 (q, $^1J_{CF}$ = 270.9 Hz), 120.8, 34.5, 31.4.

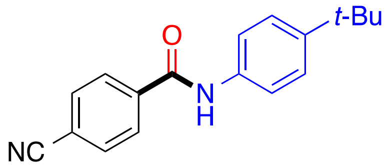


***N*-(4-(*tert*-Butyl)phenyl)-4-cyanobenzamide (3v).** Following the general procedure A, the title compound was prepared using 4-iodobenzonitrile (80 mg) and 1-(*tert*-butyl)-4-nitrobenzene (95 mg). The crude product was purified by preparative TLC using hexanes/EtOAc/$CH_2Cl_2$ (75:20:5) as an eluent to afford the title compound as a pale brown amorphous solid (56 mg, 57%). **$^1$H NMR** (400 MHz, $CDCl_3$): δ 7.98-7.94 (ovrlp, 3 H), 7.75 (d, $J$ = 7.9 Hz, 2 H), 7.54 (d, $J$ = 7.7 Hz, 2 H), 7.39 (d, $J$ = 8.2 Hz, 2 H), 1.32 (s, 9 H). **$^{13}$C NMR** (100 MHz, $CDCl_3$)**:** δ 164.0, 148.5, 139.1, 134.8, 132.7, 127.9, 126.2, 120.4, 118.1, 115.4, 34.6, 31.5. **HRMS** (ESI): Calcd for $C_{18}H_{19}N_2O$ [M+H]: 279.1497; Found: 279.1499.

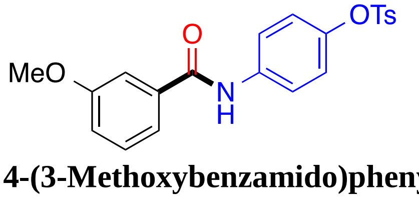


**4-(3-Methoxybenzamido)phenyl 4-methylbenzenesulfonate (3w).** Following the general procedure A, the title compound was prepared using 3-iodoanisole (82 mg) and 4-nitrophenyl 4-methylbenzenesulfonate (154 mg). The crude product was purified by preparative TLC using hexanes/EtOAc/$CH_2Cl_2$ (85:10:5) as an eluent to afford the title compound as a brown amorphous n solid (61 mg, 44%). **$^1$H NMR** (400 MHz, $CDCl_3$): δ 7.82 (s, 1 H), 7.71 (d, $J$ = 8.2 Hz, 2 H), 7.57 (d, $J$ = 9.0 Hz, 2 H), 7.42-7.36 (ovrlp, 3 H), 7.31 (d, $J$ = 8.1 Hz, 2 H), 7.09 (d, $J$ = 8.6 Hz, 1 H), 6.97 (d, $J$ = 8.9 Hz, 2 H), 3.87 (s, 3 H), 2.45 (s, 3 H). **$^{13}$C NMR** (400 MHz, $CDCl_3$): δ 165.6, 160.2, 145.9, 145.6,

136.9, 136.2, 132.3, 130.0, 129.9, 128.7, 123.2, 121.1, 118.7, 118.3, 112.7, 55.7, 21.9. **HRMS** (ESI): Calcd for $C_{21}H_{20}NO_5S$ [M+H]: 398.1062; Found: 398.1055.

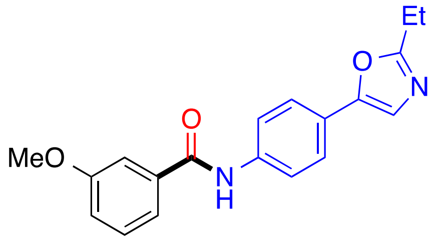


***N*-(4-(2-Ethyloxazol-5-yl)phenyl)-3-methoxybenzamide (3x).** Following the general procedure A, the title compound was prepared using 3-iodoanisole (82 mg) and 2-ethyl-5-(4-nitrophenyl)oxazole (115 mg), $Ni(glyme)Cl_2$ (15 mol %, 11.6 mg), **L1** (15 mol %, 14.1 mg), $Co_2(CO)_8$ (1 equiv, 120 mg), and DMF (1.4 mL). The crude product was purified by preparative TLC using hexanes/EtOAc/$CH_2Cl_2$ (75:20:5) as an eluent to afford the title compound as a pale brown amorphous solid (51 mg, 46%). **$^1$H NMR** (400 MHz, DMSO-$d_6$): δ 10.35 (s, 1 H), 7.89 (d, $J$ = 8.5 Hz, 2 H), 7.66 (d, $J$ = 8.5 Hz, 2 H), 7.61-7.39 (ovrlp, 4 H), 7.17 (dd, $J$ = 7.8 Hz, $J$ = 1.4 Hz, 1 H), 3.84 (s, 3 H), 2.81 (q, $J$ = 7.5 Hz, 2 H), 1.29 (t, $J$ = 7.5 Hz, 3 H). **$^{13}$C NMR** (100 MHz, DMSO-$d_6$): δ 165.3, 164.4, 159.2, 150.0, 139.0, 136.2, 129.6, 124.1, 123.1, 121.5, 120.6, 119.9, 117.4, 112.9, 55.3, 21.0, 11.0. **HRMS** (ESI): Calcd for $C_{19}H_{19}N_2O_3$ [M+H]: 323.1396; Found: 323.1395.

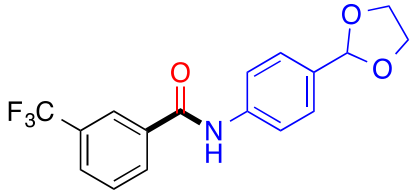


***N*-(4-(1,3-Dioxolan-2-yl)phenyl)-3-(trifluoromethyl)benzamide (3y).** Following the general procedure A, the title compound was prepared using 3-iodobenzotrifluoride (95 mg) and 2-(4-nitrophenyl)-1,3-dioxolane (1.5 equiv., 103 mg). The crude product was purified by preparative TLC using hexanes/EtOAc/$CH_2Cl_2$ (85:10:5) as an eluent to afford the title compound as a brown amorphous solid (63 mg, 54%). **$^1$H NMR** (400 MHz, DMSO-$d_6$): δ 10.54 (s, 1 H), 8.37-8.25 (ovrlp, 2 H), 7.96 (d, $J$ = 1.7 Hz, 1 H), 7.86-7.74 (ovrlp, 3 H), 7.44 (d, $J$ = 7.8 Hz, 2 H), 5.70 (s, 1 H), 4.11-4.04 (m, 2 H), 4.02-3.89 (m, 2 H). **$^{13}$C NMR** (100 MHz, DMSO-$d_6$): δ 164.0, 139.5, 135.6, 133.6, 131.8, 129.7, 129.1 (q, $^2J_{CF}$ = 31.9 Hz), 128.1 (q, $^3J_{CF}$ = 2.6 Hz), 127.0, 124.2 (q, $^3J_{CF}$ = 3.6 Hz), 123.9 (q, $^1J_{CF}$ = 271.1 Hz), 120.1, 102.6, 64.7. **HRMS** (ESI): Calcd for $C_{17}H_{15}F_3NO_3$ [M+H]: 388.1104; Found: 338.0999.

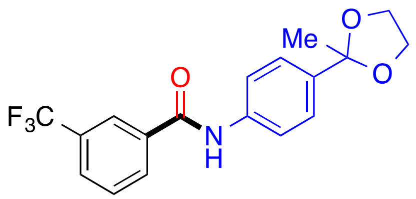


***N*-(4-(2-Methyl-1,3-dioxolan-2-yl)phenyl)-3-(trifluoromethyl)benzamide (3z).** Following the general procedure A, the title compound was prepared using 3-iodobenzotrifluoride (95 mg) and 2-methyl-2-(4-nitrophenyl)-1,3-dioxolane (110 mg). The crude product was purified by preparative TLC using hexanes/EtOAc/$CH_2Cl_2$ (85:10:5) as an eluent to afford the title compound as a pale-brown amorphous solid (98 mg, 80%). **$^1$H NMR** (400 MHz, DMSO-$d_6$): δ 10.49 (s, 1 H), 8.35-8.21 (ovrlp, 2 H), 7.96 (d, *J* = 4.8 Hz, 1 H), 7.83-7.69 (ovrlp, 3 H), 7.42 (d, *J* = 5.7 Hz, 2 H), 4.05-3.91 (m, 2 H), 3.77-3.65 (m, 2 H), 1.56 (s, 3 H). **$^{13}$C NMR** (100 MHz, DMSO-$d_6$): δ 164.0, 138.8, 138.3, 135.7, 131.8, 129.7, 129.2 (q, $^2J_{CF}$ = 32.1 Hz), 128.1 (q, $^3J_{CF}$ = 2.4 Hz), 125.4, 124.2 (q, $^3J_{CF}$ = 3.2 Hz), 124.0 (q, $^1J_{CF}$ = 271.3 Hz), 120.2, 108.0, 64.0, 27.2. **HRMS** (ESI): Calcd for $C_{18}H_{17}F_3NO_3$ [M+H]: 352.1161; Found: 352.1154.

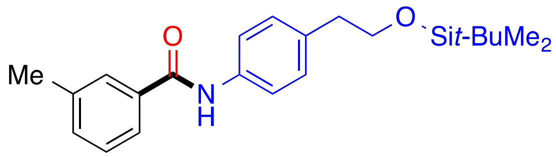


***N*-(4-(2-((*tert*-Butyldimethylsilyl)oxy)ethyl)phenyl)-3-methylbenzamide (3aa).** Following the general procedure A, the title compound was prepared using 3-iodotoluene (76 mg) and *tert*-butyldimethyl(4-nitrophenethoxy)silane (139 mg). The crude product was purified by preparative TLC using hexanes/EtOAc/$CH_2Cl_2$ (90:5:5) as an eluent to afford the title compound as a viscous brown solid (64 mg, 50%). **$^1$H NMR** (400 MHz, $CDCl_3$): δ 7.98 (s, 1 H), 7.66 (s, 1 H), 7.62 (t, *J* = 3.2 Hz, 1 H), 7.55 (d, *J* = 8.0 Hz, 2 H), 7.34-7.28 (ovrlp, 2 H), 7.18 (d, *J* = 8.1 Hz, 2 H), 3.79 (t, *J* = 6.9 Hz, 2 H), 2.80 (t, *J* = 6.9 Hz, 2 H), 2.38 (s, 3 H), 0.88 (s, 9 H). **$^{13}$C NMR** (100 MHz, $CDCl_3$): δ 166.0, 138.7, 136.3, 135.5, 135.1, 132.5, 129.8, 128.6, 127.9, 124.1, 120.3, 64.6, 39.2, 26.1, 21.5, 18.4. -5.2. **HRMS** (ESI): Calcd for $C_{22}H_{32}NO_2Si$ [M+H]: 370.2202; Found: 370.2199.

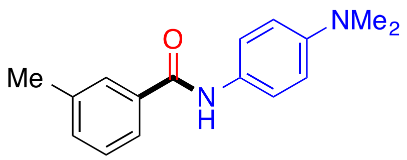


***N*-(4-(Dimethylamino)phenyl)-3-methylbenzamide (3bb).** Following the general procedure A, the title compound was prepared using 3-iodotoluene (76 mg) and *N*,*N*-dimethyl-4-nitroaniline (87 mg). The crude product was purified by preparative TLC using hexanes/EtOAc/$CH_2Cl_2$ (75:20:5) as an eluent to afford the title compound as a pale brown amorphous solid (65 mg, 73%). **$^1$H NMR** (400 MHz, $CDCl_3$): δ 7.93 (s, 1 H), 7.65 (s, 1 H), 7.60 (s, 1 H), 7.47 (d, *J* = 8.3 Hz, 2 H), 7.31-7.25 (ovrlp, 2 H), 6.69 (d, *J* = 8.6 Hz, 2 H), 2.90 (s, 6 H), 2.36 (s, 3 H). **$^{13}$C NMR** (100 MHz, $CDCl_3$): δ 165.9, 148.1,

138.5, 135.3, 132.2, 128.5, 128.0, 127.9, 124.0, 122.2, 113.1, 41.0, 21.4. **HRMS** (ESI): Calcd for $C_{16}H_{19}N_2O$ [M+H]: 255.1497; Found: 255.1501.

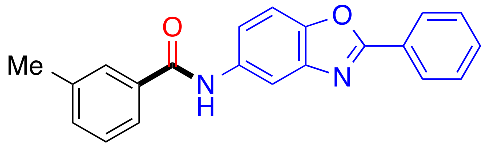

**3-Methyl-*N*-(2-phenylbenzo[*d*]oxazol-5-yl)benzamide (3cc).** Following the general procedure A, the title compound was prepared using 3-iodotoluene (76 mg), 5-nitro-2-phenylbenzo[*d*]oxazole (126 mg), Ni(glyme)$Cl_2$ (15 mol %, 11.6 mg), **L1** (15 mol %, 14.1 mg), $Co_2(CO)_8$ (1 equiv, 120 mg), and DMF (1.4 mL). The crude product was purified by preparative TLC using hexanes/EtOAc/$CH_2Cl_2$ (85:10:5) as an eluent to afford the title compound as a off-white amorphous solid (93 mg, 81%). **$^1$H NMR** (400 MHz, DMSO-$d_6$): δ 10.39 (s, 1 H), 8.30 (s, 1 H), 8.24-8.17 (ovrlp, 2 H), 7.82-7.75 (ovrlp, 4 H), 7.64-7.59 (ovrlp, 3 H), 7.45-7.39 (ovlp, 2 H), 2.41 (s, 3 H). **$^{13}$C NMR** (100 MHz, DMSO-$d_6$): δ 165.7, 162.9, 146.6, 141.6, 137.7, 136.4, 134.9, 132.2, 131.9, 129.3, 128.3, 128.1, 127.2, 126.4, 124.8, 118.8, 111.2, 110.5, 21.0. **HRMS** (ESI): Calcd for $C_{21}H_{17}N_2O_2$ [M+H]: 329.1290; Found: 329.1291.

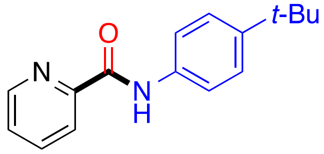

***N*-(4-(*tert*-Butyl)phenyl)picolinamide (3dd).** Following the general procedure A, the title compound was prepared using 2-iodopyridine (72 mg) and 1-(*tert*-butyl)-4-nitrobenzene (95 mg), Ni(glyme)$Cl_2$ (15 mol %, 11.6 mg), **L1** (15 mol %, 14.1 mg), and $Co_2(CO)_8$ (1 equiv, 120 mg). The crude product was purified by preparative TLC using hexanes/EtOAc/$CH_2Cl_2$ (75:20:5) as an eluent to afford the title compound as an off-white amorphous solid (46 mg, 51%). **$^1$H NMR** (400 MHz, $CDCl_3$): δ 9.97 (s, 1 H), 8.61 (d, $J$ = 3.7 Hz, 1 H), 8.30 (d, $J$ = 7.6 Hz, 1 H), 7.90 (t, $J$ = 7.5 Hz, 1 H), 7.70 (d, $J$ = 8.4 Hz, 2 H), 7.47 (dd, $J$ = 6.4 Hz, $J$ = 5.2 Hz, 1 H), 7.41 (d, $J$ = 8.4 Hz, 2 H), 1.33 (s, 9 H). **$^{13}$C NMR** (100 MHz, $CDCl_3$): δ 162.0, 150.1, 148.1, 147.4, 137.8, 135.3, 126.5, 126.0, 122.5, 119.6, 34.6, 31.5. **HRMS** (ESI): Calcd for $C_{16}H_{19}N_2O$ [M+H]: 255.1497; Found: 255.1503.

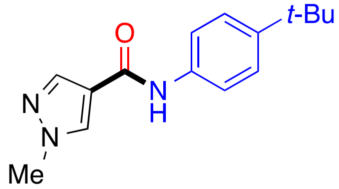

***N*-(4-(*tert*-Butyl)phenyl)-1-methyl-1*H*-pyrazole-4-carboxamide (3ee).** Following the general procedure A, the title compound was prepared using 4-iodo-1-methyl-1*H*-pyrazole (73 mg) and 1-(*tert*-butyl)-4-nitrobenzene (1.5 equiv., 88 mg). The crude product was purified by preparative TLC using hexanes/EtOAc/$CH_2Cl_2$ (65:30:5) as an eluent to afford the title compound as a brown amorphous solid (59 mg, 66%). **$^1$H NMR** (400 MHz, $CDCl_3$): δ 8.29 (s, 1 H), 8.03-7.86 (ovrlp, 2 H), 7.52 (d, $J$ = 7.2 Hz,

2 H), 7.30 (d, $J$ = 7.5 Hz, 2 H), 3.78 (s, 3 H), 1.28 (s, 9 H). $^{13}$**C NMR** (100 MHz, $CDCl_3$): δ 161.4 (br), 147.4, 138.9 (br), 135.4, 132.8 (br), 125.8, 120.5, 119.5 (br), 39.3, 34.4, 31.4. **HRMS** (ESI): Calcd for $C_{15}H_{20}N_3O$ [M+H]: 258.1606; Found: 258.1607.

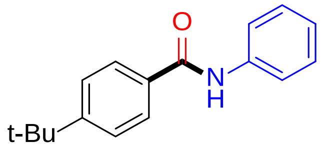


**4-(*tert*-butyl)-N-phenylbenzamide (3ff).** The title compound was prepared using 4-(*tert*-butyl)-1-iodobenzene (92.8 mg) and nitrobenzene (69.8 mg). The crude product was purified by preparative TLC using hexanes/EtOAc/$CH_2Cl_2$ (170/20/10) as an eluent to afford the title compound as an off-white amorphous solid (53 mg, 59%). $^1$**H NMR** (400 MHz, $CDCl_3$): δ 7.93 (s, 1H), 7.81 (d, $J$ = 8.4 Hz, 2H), 7.65 (d, $J$ = 7.2 Hz, 1H), 7.48 (d, $J$ = 8.4 Hz, 2H), 7.36 (t, $J$ = 7.8 Hz, 2H), 7.14 (t, $J$ = 7.4 Hz, 1H), 1.35 (s, 9H). $^{13}$**C NMR** (100 MHz, $CDCl_3$): δ 165.72, 155.41, 138.09, 132.11, 129.06, 126.91, 125.71, 124.41, 120.20, 77.37, 77.05, 76.73, 35.00, 31.18. **HRMS** (ESI): Calcd for $C_{17}H_{20}NO$ [M+H]: 254.1545; Found: 254.1541

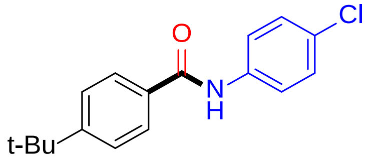


**4-(*tert*-butyl)-N-(4-chlorophenyl)benzamide (3gg).** The title compound was prepared using 4-(*tert*-butyl)-1-iodobenzene (97.1 mg) and 1-chloro-4-nitrobenzene (85.4 mg). The crude product was purified by preparative TLC using hexanes/EtOAc/$CH_2Cl_2$ (170/20/10) as an eluent to afford the title compound as a light-yellow amorphous solid (47 mg, 44%). $^1$**H NMR** (400 MHz, DMSO-$d_6$) δ 10.29 (s, 1H), 7.88 (d, $J$ = 8.4 Hz, 2H), 7.81 (d, $J$ = 8.9 Hz, 2H), 7.54 (d, $J$ = 8.4 Hz, 2H), 7.40 (d, $J$ = 8.8 Hz, 2H), 1.32 (s, 9H). $^{13}$**C NMR** (101 MHz, DMSO) δ 166.05, 155.04, 138.72, 132.47, 128.96, 128.01, 127.56, 125.65, 122.20, 40.63, 40.42, 40.21, 40.00, 39.79, 39.58, 39.37, 35.17, 31.40. **HRMS** (ESI): Calcd for $C_{17}H_{19}ClNO$ [M+H]: 288.1155; Found: 288.1147

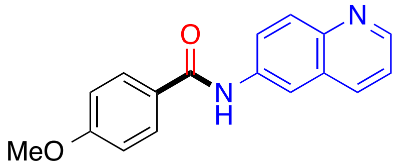


**4-Methoxy-*N*-(quinolin-6-yl)benzamide (4a).** Following the general procedure B, the title compound was prepared using 4-bromoanisole (66 mg), 6-nitroquinoline (91 mg), Ni(glyme)$Cl_2$ (15 mol %, 11.6 mg), and **L4** (15 mol %, 14.1 mg). The crude product was purified by preparative TLC using hexanes/EtOAc/$CH_2Cl_2$ (65:30:5) as an eluent to afford the title compound as a brown amorphous solid (70 mg, 71%). $^1$**H NMR** (400 MHz, DMSO-$d_6$): δ 10.43 (s, 1 H), 8.81 (dd, $J$ = 3.9 Hz, $J$ = 1.2 Hz, 1 H),

8.54 (d, $J$ = 2.1 Hz, 1 H), 8.32 (dd, $J$ = 8.4 Hz, $J$ = 0.8 Hz, 1 H), 8.09-8.00 (ovrlp, 4 H), 7.50 (dd, $J$ = 8.3 Hz, $J$ = 4.2 Hz, 1 H), 7.09 (d, $J$ = 8.8 Hz, 2 H), 3.85 (s, 3 H). **$^{13}$C NMR** (100 MHz, DMSO-$d_6$): δ 165.3, 162.1, 148.9, 144.6, 137.4, 135.7, 129.7, 129.1, 128.3, 126.7, 124.4, 121.7, 116.1, 113.7, 55.4. **HRMS** (ESI): Calcd for $C_{17}H_{15}N_2O_2$ [M+H]: 279.1133; Found: 279.1142.

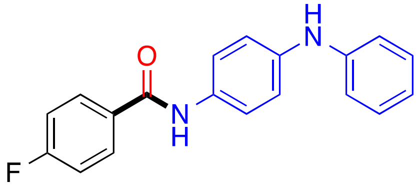


**4-Fluoro-*N*-(4-(phenylamino)phenyl)benzamide (4b).** Following the general procedure B, the title compound was prepared using 1-fluoro-4-nitrobenzene (61 mg) and 4-nitro-*N*-phenylaniline (112 mg). The crude product was purified by preparative TLC using hexanes/EtOAc/$CH_2Cl_2$ (75:20:5) as an eluent to afford the title compound as a brown amorphous solid (78 mg, 73%). **$^1$H NMR** (400 MHz, DMSO-$d_6$): δ 10.12 (s, 1 H), 8.09 (s, 1 H), 8.03 (dd, $^3J_{HH}$ = 8.6 Hz, $^4J_{HF}$ = 5.7 Hz, 2 H), 7.63 (d, $J$ = 8.0 Hz, 2 H), 7.35 (dd, $^3J_{HH}$ = 8.3 Hz, $^3J_{HF}$ = 8.3 Hz, 2 H), 7.21 (t, $J$ = 7.0 Hz, 2 H), 7.09-7.03 (ovrlp, 4 H), 6.78 (t, $J$ = 6.7 Hz, 1 H). **$^{13}$C NMR** (100 MHz. $CDCl_3$): δ 163.93 (d, $^1J_{CF}$ = 246.6 Hz), 163.90, 143.9, 139.4, 131.7, 131.5 (d, $^4J_{CF}$ = 2.2 Hz), 130.2 (d, $^3J_{CF}$ = 8.7 Hz), 129.1, 121.8, 119.1, 117.4, 116.0, 115.2 (d, $^2J_{CF}$ = 21.7). **HRMS** (ESI): Calcd for $C_{19}H_{16}FN_2O$ [M+H]: 307.1247; Found: 307.1247.

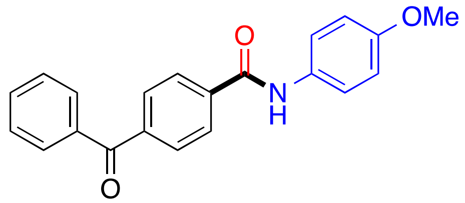


**4-Benzoyl-*N*-(4-methoxyphenyl)benzamide (4c).** Following the general procedure B, the title compound was prepared using (4-bromophenyl)(phenyl)methanone (91 mg) and 4-nitroanisole (80 mg). The crude product was purified by preparative TLC using hexanes/EtOAc/$CH_2Cl_2$ (75:20:5) as an eluent to afford the title compound as a pale brown amorphous solid (55 mg, 47%). **$^1$H NMR** (400 MHz, $CDCl_3$): δ 7.97 (d, $J$ = 7.8 Hz, 2 H), 7.90-7.86 (ovrlp, 3 H), 7.81 (d, $J$ = 7.4 Hz, 2 H), 7.63 (t = $J$ = 8.6 Hz, 1 H), 7.57 (d, $J$ = 8.4 Hz, 2 H), 7.51 (t, $J$ = 7.7 Hz, 2 H), 6.93 (d, $J$ = 8.6 Hz, 2 H), 3.82 (s, 3 H). **$^{13}$C NMR** (100 MHz, $CDCl_3$): δ 196.0, 164.9, 157.0, 140.4, 138.4, 137.1, 133.1, 130.8, 130.4, 130.2, 128.6, 127.1, 122.3, 114.5, 55.7. **HRMS** (ESI): Calcd for $C_{21}H_{18}NO_3$ [M+H]: 332.1287; Found: 322.1286.

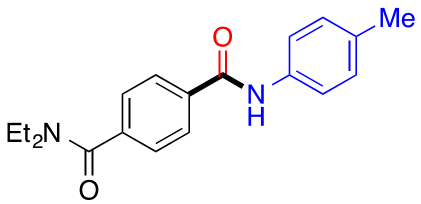

***N*[1],*N*[1]-Diethyl-*N*[4]-(*p*-tolyl)terephthalamide (4d).** Following the general procedure B, the title compound was prepared using 4-bromo-*N*,*N*-diethylbenzamide (90 mg) and 4-nitrotoluene (72 mg). The crude product was purified by preparative TLC using hexanes/EtOAc/$CH_2Cl_2$ (65:30:5) as an eluent to afford the title compound as a pale-brown amorphous solid (88 mg, 81%). **$^1$H NMR** (400 MHz, $CDCl_3$): δ 9.15 (s, 1 H), 7.80 (d, *J* = 8.2 Hz, 2 H), 7.67 (d, *J* = 8.3 Hz, 2 H), 7.19 (d, *J* = 8.2 Hz, 2 H), 7.14 (d, *J* = 8.2 Hz, 2 H), 3.54 (q, *J* = 6.8 Hz, 2 H), 3.17 54 (q, *J* = 6.7 Hz, 2 H), 2.33 (s, 3 H), 1.24 (t, *J* = 6.5 Hz, 3 H), 1.06 t, *J* = 6.5 Hz, 3 H). **$^{13}$C NMR** (100 MHz, $CDCl_3$): δ 170.8, 165.6, 139.4, 136.0, 134.0, 129.4, 127.7, 126.1, 120.6, 43.4, 39.6, 21.0, 14.2, 13.0. **HRMS** (ESI): Calcd for $C_{19}H_{23}N_2O_2$ [M+H]: 311.1760; Found: 311.1771.

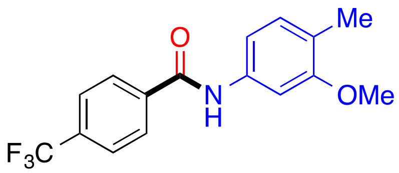

***N*-(3-Methoxy-4-methylphenyl)-4-(trifluoromethyl)benzamide (4e).** Following the general procedure B, the title compound was prepared using 4-bromobenzotrifluoride (79 mg) and 2-methoxy-4-nitrotoluene (88 mg). The crude product was purified by preparative TLC using hexanes/EtOAc/$CH_2Cl_2$ (75:20:5) as an eluent to afford the title compound as a pale brown amorphous solid (88 mg, 81%). **$^1$H NMR** (400 MHz, $CD_2Cl_2$): δ 8.07 (s, 1 H), 7.97 (d, *J* = 7.3 Hz, 2 H), 7.73 (d, *J* = 7.3 Hz, 2 H), 7.36 (s, 1 H), 7.10 (d, *J* = 7.5 Hz, 1 H), 7.02 (d, *J* = 6.7 Hz, 1 H), 3.82 (s, 3 H), 2.19 (s, 3 H). **$^{13}$C NMR** (100 MHz, $CD_2Cl_2$): δ 164.7, 158.5, 139.0, 137.2, 133.5 (q, $^2J_{CF}$ = 32.5 Hz), 130.9, 128.0, 126.2 (q, $^3J_{CF}$ = 3.7 Hz), 124.3 (q, $^1J_{CF}$ = 270.7 Hz), 123.7, 112.4, 103.7, 55.8, 15.9. **HRMS** (ESI): Calcd for $C_{16}H_{15}F_3NO_2$ [M+H]: 310.1055; Found: 310.1063.

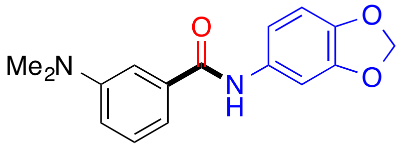

***N*-(Benzo[*d*][1,3]dioxol-5-yl)-3-(dimethylamino)benzamide (4f).** Following the general procedure B, the title compound was prepared using 4-bromo-*N*,*N*-diethylbenzamide (70 mg) and 5-nitrobenzo[*d*][1,3]dioxole (88 mg). The crude product was purified by preparative TLC using hexanes/EtOAc/$CH_2Cl_2$ (75:20:5) as an eluent to afford the title compound as an off-white amorphous solid (70 mg, 71%). **$^1$H NMR** (400 MHz, $CDCl_3$): δ 7.99 (s, 1 H), 7.34 (d, *J* = 1.8 Hz, 1 H), 7.26-7.21 (ovrlp, 2 H), 7.04 (d, *J* = 7.5 Hz, 1 H), 6.90 (dd, *J* = 8.3 Hz, *J* = 2.0 Hz, 1 H), 6.83 (d, *J* = 8.2 Hz, *J* = 2.4 Hz, 1 H), 6.73 (d, *J* = 8.3 Hz, 1 H), 5.96 (s, 2 H), 2.96 (s, 9 H). **$^{13}$C NMR** (100 MHz, $CDCl_3$): δ

166.7, 150.8, 147.8, 144.4, 135.8, 132.5, 129.3, 115.5, 114.0, 113.6, 111.4, 108.1, 103.3, 101.3, 40.5. **HRMS** (ESI): Calcd for $C_{16}H_{17}N_2O_3$ [M+H]: 285.1239; Found: 285.1239.

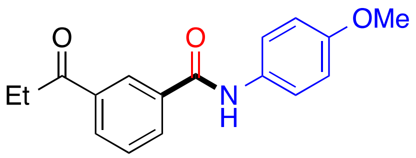


***N*-(4-Methoxyphenyl)-3-propionylbenzamide (4g).** Following the general procedure B, the title compound was prepared using 1-(4-bromophenyl)propan-1-one (75 mg) and 4-nitroanisole (80 mg). The crude product was purified by preparative TLC using hexanes/EtOAc/$CH_2Cl_2$ (75:20:5) as an eluent to afford the title compound as a brown amorphous solid (50 mg, 51%). **$^1$H NMR** (400 MHz, $CDCl_3$): δ 8.41 (s, 1 H), 8.11-8.06 (ovrlp, 2 H), 8.03 (s, 1 H), 7.59-7.52 (ovrlp, 3 H), 6.90 (d, $J$ = 8.4 Hz, 2 H), 3.81 (s, 3 H), 3.04 (q, $J$ = 7.0 Hz, 2 H), 1.23 (t, $J$ = 7.0 Hz, 3 H). **$^{13}$C NMR** (100 MHz, $CDCl_3$): δ 200.2, 165.0, 156.9, 137.2, 135.6, 131.7, 131.0, 130.9, 129.2, 126.3, 122.4, 114.3, 55.6, 32.1, 8.2. **HRMS** (ESI): Calcd for $C_{17}H_{18}NO_3$ [M+H]: 284.1287; Found: 284.1289.

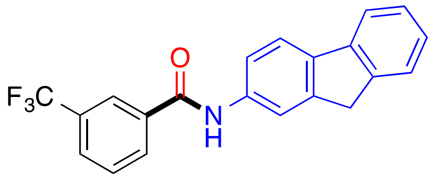


***N*-(9*H*-Fluoren-2-yl)-3-(trifluoromethyl)benzamide (4h).** Following the general procedure B, the title compound was prepared using 3-bromobenzotrifluoride (79 mg) and 2-nitro-9*H*-fluorene (111 mg). The crude product was purified by preparative TLC using hexanes/EtOAc/$CH_2Cl_2$ (85:10:5) as an eluent to afford the title compound as a pale brown amorphous solid (62 mg, 50%). **$^1$H NMR** (400 MHz, $CDCl_3$): δ 8.15 (s, 1 H), 8.09 (d, $J$ = 7.8 Hz, 1 H), 8.02 (s, 1 H), 7.93 (s, 1 H), 7.83 (d, $J$ = 7.8 Hz, 1 H), 7.79-7.75 (ovrlp, 2 H), 7.65 (t, $J$ = 7.8 Hz, 1 H), 7.56-7.50 (ovrlp, 2 H), 7.38 (t, $J$ = 7.4 Hz, 1 H), 7.30 (td, $J$ = 7.4 Hz, $J$ = 1.1 Hz, 1 H), 3.94 (s, 2 H). **$^{13}$C NMR** (100 MHz, $CDCl_3$): δ 164.4, 144.6, 143.4, 141.3, 139.0, 136.4, 136.1, 131.6 (q, $^2J_{CF}$ = 32.7 Hz), 130.5, 129.7, 128.6 (q, $^3J_{CF}$ = 3.6 Hz), 127.0, 126.7, 125.2, 124.1 (q, $^3J_{CF}$ = 3.8 Hz), 122.7 (q, $^1J_{CF}$ = 270.8 Hz), 120.4, 119.8, 119.3, 117.5, 37.2. **HRMS** (ESI): Calcd for $C_{21}H_{15}F_3NO$ [M+H]: 354.1106; Found: 354.1109.

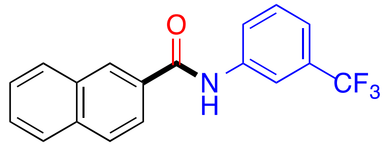


***N*-(3-(Trifluoromethyl)phenyl)-2-naphthamide (4i).**[16] Following the general procedure B, the title compound was prepared using 2-bromonaphthalene (73 mg), 3-nitrobenzotrifluoride (100 mg), Ni(glyme)$Cl_2$ (15 mol %, 11.6 mg), and **L4** (15 mol %, 14.1 mg). The crude product was purified by preparative TLC using hexanes/EtOAc/$CH_2Cl_2$ (85:10:5) as an eluent to afford the title compound as an

off-write amorphous solid (64 mg, 58%). **$^1$H NMR** (400 MHz, $CDCl_3$): δ 8.33 (s, 1 H), 8.27 (s, 1 H), 7.98 (s, 1 H), 7.93-7.85 (ovrlp, 5 H), 7.60-7.52 (ovrlp, 2 H), 7.47 (t, $J$ = 8.0 Hz, 1 H), 7.40 (d, $J$ = 7.8 Hz, 1 H). **$^{13}$C NMR** (100 MHz, $CDCl_3$): δ 138.7, 135.1, 132.7, 131.7, 131.6 (q, $^2J_{CF}$ = 32.3 Hz), 129.8, 129.1, 129.0, 128.3, 128.1, 128.0, 127.8, 127.2 (q, $^1J_{CF}$ = 270.6 Hz), 123.52, 123.46 (q, $^4J_{CF}$ = 0.8 Hz), 121.2 (q, $^3J_{CF}$ = 3.8 Hz), 117.1 (q, $^3J_{CF}$ = 4.0 Hz).

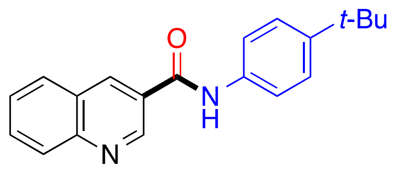


***N*-(4-(*tert*-Butyl)phenyl)quinoline-3-carboxamide (4j).** Following the general procedure B, the title compound was prepared using 3-bromoquinoline (73 mg) and 1-(*tert*-butyl)-4-nitrobenzene (95 mg). The crude product was purified by preparative TLC using hexanes/EtOAc/$CH_2Cl_2$ (75:20:5) as an eluent to afford the title compound as a off-white amorphous solid (85 mg, 80%). **$^1$H NMR** (400 MHz, $CDCl_3$): δ 9.35 (s, 1 H), 8.65 (s, 1 H), 8.34 (s, 1 H), 8.12 (d, $J$ = 8.4 Hz, 1 H), 7.86 (d, $J$ = 7.9 Hz, 1 H), 7.79 (t, $J$ = 7.3 Hz, 1 H), 7.64-7.56 (ovrlp, 3 H), 7.39 (d, $J$ = 8.4 Hz, 2 H), 1.32 (s, 9 H). **$^{13}$C NMR** (100 MHz, $CDCl_3$): δ 164.1, 149.2, 148.24, 148.19, 136.1, 135.1, 131.6, 129.4, 128.9, 127.8, 127.0, 126.1, 120.5, 34.6, 31.5. **HRMS** (ESI): Calcd for $C_{20}H_{21}N_2O$ [M+H]: 305.1654; Found: 305.1649.

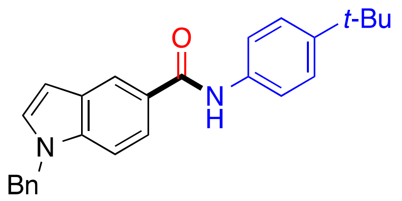


**1-Benzyl-*N*-(4-(*tert*-butyl)phenyl)-1*H*-indole-5-carboxamide (4k).** Following the general procedure B, the title compound was prepared using 1-benzyl-5-bromo-1*H*-indole (100 mg) and 1-(*tert*-butyl)-4-nitrobenzene (95 mg). The crude product was purified by preparative TLC using hexanes/EtOAc/$CH_2Cl_2$ (85:10:5) as an eluent to afford the title compound as a brown amorphous solid (57 mg, 43%). **$^1$H NMR** (400 MHz, $CDCl_3$): δ 8.09 (d, $J$ = 0.9 Hz, 1 H), 7.87 (s, 1 H), 7.59 (dd, $J$ = 8.6 Hz, $J$ = 1.4 Hz, 1 H), 7.49 (d, $J$ = 8.6 Hz, 2 H), 7.27 (d, $J$ = 8.6 Hz, 2 H), 7.21-7.17 (ovrlp, 4 H), 7.09 (d, $J$ = 3.1 Hz, 1 H), 7.00-6.97 (ovrlp, 2 H), 6.52 (d, $J$ = 3.1 Hz, 1 H), 5.22 (s, 2 H), 1.23 (s, 9 H). **$^{13}$C NMR** (100 MHz, $CDCl_3$): δ 166.9, 147.1, 138.1, 137.0, 135.9, 130.0, 129.0, 128.5, 127.9, 126.83, 126.77, 125.9, 121.0, 120.8, 120.1, 109.9, 103.1, 50.4, 34.5, 31.5. **HRMS** (ESI): Calcd for $C_{26}H_{27}N_2O$ [M+H]: 383.2123; Found: 383.2115.

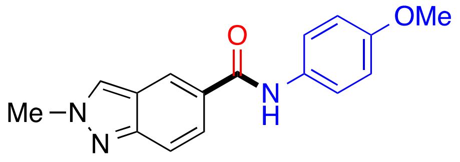

***N*-(4-methoxyphenyl)-2-methyl-2*H*-indazole-5-carboxamide (4l).** Following the general procedure B, the title compound was prepared using 5-bromo-2-methyl-2*H*-indazole (74 mg), 4-nitroanisole (120 mg), Ni(glyme)$Cl_2$ (15 mol %, 11.6 mg), and **L4** (15 mol %, 11.4 mg). The crude product was purified by preparative TLC using hexanes/$CH_2Cl_2$ (90:10) as an eluent to afford the title compound as an off-white amorphous solid (53 mg, 53%). $^1$**H NMR** (400 MHz, DMSO-$d_6$): δ 10.09 (s, 1 H), 8.57 (s, 1 H), 8.42 (s, 1 H), 7.78 (d, *J* = 9.1 Hz, 1 H), 7.70-7.65 (ovrlp, 3 H), 6.93 (d, *J* = 8.5 Hz, 2 H), 4.21 (s, 3 H), 3.75 (s, 3 H). $^{13}$**C NMR** (100 MHz, DMSO-$d_6$): δ 165.5, 155.4, 148.8, 132.5, 127.7, 126.7, 124.5, 121.9, 121.5, 120.8, 116.5, 113.7, 55.2, 40.2. **HRMS** (ESI): Calcd for $C_{16}H_{16}N_3O_2$ [M+H]: 282.1242; Found: 282.1247.

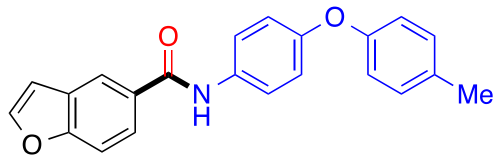


***N*-(4-(*p*-Tolyloxy)phenyl)benzofuran-5-carboxamide (4m).** Following the general procedure B, the title compound was prepared using 5-bromobenzofuran (69 mg), 1-methyl-4-(4-nitrophenoxy)benzene (120 mg), Ni(glyme)$Cl_2$ (15 mol %, 11.6 mg), and **L4** (15 mol %, 11.4 mg). The crude product was purified by preparative TLC using hexanes/EtOAc/$CH_2Cl_2$ (85:10:5) as an eluent to afford the title compound as a pale-brown amorphous solid (55 mg, 46%). $^1$**H NMR** (400 MHz. $CDCl_3$): δ 8.14 (s, 1 H), 7.94 (s, 1 H), 7.81 (d, *J* = 8.4 Hz, 1 H), 7.70 (d, *J* = 0.8 Hz, 1 H), 7.59-7.54 (ovrlp, 3 H), 7.13 (d, *J* = 8.0 Hz, 2 H), 6.99 (d, *J* = 8.7 Hz, 2 H), 6.91 (d, *J* = 8.2 Hz, 2 H), 6.83 (s, 1 H), 2.33 (s, 9 H). $^{13}$**C NMR** (100 MHz. $CDCl_3$): δ 166.1, 156.8, 155.1, 154.4, 146.6, 133.3, 132.9, 130.4, 130.2, 127.9, 123.6, 122.2, 120.9, 119.2, 118.9, 111.8, 107.1, 20.8. **HRMS** (ESI): Calcd for $C_{22}H_{18}NO_3$ [M+H]: 344.1287; Found: 344.1278.

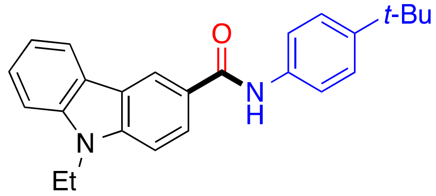


***N*-(4-(*tert*-Butyl)phenyl)-9-ethyl-9*H*-carbazole-3-carboxamide (4n).** Following the general procedure B, the title compound was prepared using 3-bromo-9-ethyl-9*H*-carbazole (96 mg) and 1-(*tert*-butyl)-4-nitrobenzene (95 mg). The crude product was purified by preparative TLC using hexanes/EtOAc/$CH_2Cl_2$ (85:10:5) as an eluent to afford the title compound as a brown amorphous solid (91 mg, 70%). $^1$**H NMR** (400 MHz. $CDCl_3$): δ 8.59 (s, 1 H), 8.11 (s, 1 H), 8.07 (d, *J* = 7.6 Hz, 1 H), 7.95 (d, *J* = 8.2 Hz, 1 H), 7.64 (d, *J* = 8.0 Hz, 2 H), 7.49 (t, *J* = 7.4 Hz, 1 H), 7.43-7.31 (ovlp, 4 H), 7.24 (t, *J* = 7.9 Hz, 1 H), 4.30 (q, *J* = 7.6 Hz, 2 H), 1.39 (t, *J* = 6.8 Hz, 3 H), 1.33 (s, 9 H). $^{13}$**C NMR** (100 MHz. $CDCl_3$): δ 166.6, 147.3, 141.9, 140.7, 136.0, 126.5, 126.0, 125.6, 124.9, 123.0, 122.9, 120.8, 120.2, 120.1, 119.8, 109.0, 108.4, 37.9, 34.5, 31.5, 13.9. **HRMS** (ESI): Calcd for $C_{25}H_{27}N_2O$ [M+H]: 371.2123; Found: 371.2118.

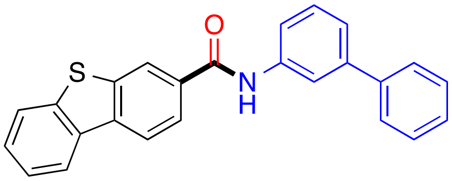


***N*-([1,1'-Biphenyl]-3-yl)dibenzo[*b*,*d*]thiophene-3-carboxamide (4o).** Following the general procedure B, the title compound was prepared using 3-bromodibenzo[*b*,*d*]thiophene (92 mg) and 3-nitro-1,1'-biphenyl (105 mg). The crude product was purified by preparative TLC using hexanes/EtOAc/$CH_2Cl_2$ (85:10:5) as an eluent to afford the title compound as a off-white amorphous solid (110 mg, 83%). **$^1$H NMR** (400 MHz, DMSO-$d_6$): δ 10.53 (s, 1 H), 9.03 (s, 1 H), 8.51-8.46 (m, 1 H), 8.22-8.14 (ovrlp, 3 H), 8.10-8.06 (m, 1 H), 7.92 (d, $J$ = 8.0 Hz, 1 H), 7.69 (d, $J$ = 7.4 Hz, 2 H), 7.61-7.55 (ovrlp, 2 H), 7.52-7.46 (ovrlp, 3 H), 7.44-7.37 (ovrlp, 2 H). **$^{13}$C NMR** (100 MHz, DMSO-$d_6$): δ 165.4, 141.9, 140.7, 140.2, 139.8, 139.1, 134.9, 134.8, 131.5, 129.3, 129.0, 127.6, 126.6, 126.2, 125.1, 123.2, 123.0, 122.3, 122.0, 121.3, 119.4, 118.7. **HRMS** (ESI): Calcd for $C_{25}H_{18}NOS$ [M+H]: 380.1109; Found: 380.1105.

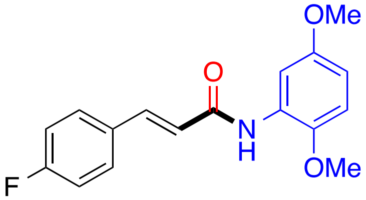


**(*E*)-*N*-(2,5-Dimethoxyphenyl)-3-(4-fluorophenyl)acrylamide (4p).** Following the general procedure B, the title compound was prepared using (*E*)-1-(2-bromovinyl)-4-fluorobenzene (70 mg) and 1,4-dimethoxy-2-nitrobenzene (96 mg). The crude product was purified by preparative TLC using hexanes/EtOAc/$CH_2Cl_2$ (75:20:5) as an eluent to afford the title compound as a brown amorphous solid (46 mg, 44%). **$^1$H NMR** (400 MHz, $CDCl_3$): δ 8.27 (s, 1 H), 7.97 (s, 1 H), 7.70 (d, $J$ = 15.5 Hz, 1 H), 7.53 (dd, $^3J_{HH}$ = 8.6 Hz, $^4J_{HF}$ = 5.4 Hz, 2 H), 7.07 (dd, $^3J_{HH}$ = 8.6 Hz, $^3J_{HF}$ = 8.6 Hz, 2 H), 6.81 (d, $J$ = 8.9 Hz, 1 H), 6.60 (dd, $J$ = 8.9 Hz, $J$ = 3.0 Hz, 1 H), 6.51 (d, $J$ = 15.5 Hz, 1 H), 3.87 (s, 3 H), 3.81 (s, 3 H). **$^{13}$C NMR** (100 MHz, $CDCl_3$): δ 163.8 (d, $^1J_{CF}$ = 249.2 Hz), 163.7, 154.1, 142.2, 141.0, 131.1, 129.9 (d, $^3J_{CF}$ = 8.4 Hz), 128.6, 121.1 (d, $^4J_{CF}$ = 1.6 Hz), 116.1 (d, $^2J_{CF}$ = 21.8 Hz), 110.8, 109.0, 106.1, 56.4, 55.9. **HRMS** (ESI): Calcd for $C_{17}H_{17}FNO_3$ [M+H]: 302.1192; Found: 302.1194.

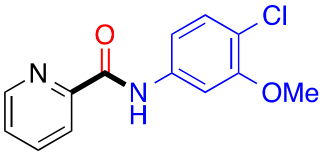


***N*-(4-Chloro-3-methoxyphenyl)picolinamide (5).**[17] Following the general procedure A, the title compound was prepared using 2-iodopyridine (72 mg), 2-chloro-5-nitroanisole (99 mg), Ni(glyme)$Cl_2$ (15 mol %, 11.6 mg), **L1** (15 mol %, 14.1 mg), $Co_2(CO)_8$ (1 equiv, 120 mg), and DMF (1.4 mL). The crude product was purified by preparative TLC using hexanes/EtOAc/$CH_2Cl_2$ (75:20:5) as an eluent to

afford the title compound as an off-white amorphous solid (58 mg, 63%). **$^{1}$H NMR** (400 MHz, DMSO-$d_6$): δ 10.76 (s, 1 H), 8.75 (d, $J$ = 4.6 Hz, 1 H), 8.16 (d, $J$ = 7.8 Hz, 1 H), 8.08 (td, $J$ = 7.6 Hz, $J$ = 4.0 Hz, 1 H), 7.84 (d, $J$ = 2.2 Hz, 1 H), 7.69 (ddd, $J$ = 7.4 Hz, $J$ = 4.8 Hz, $J$ = 1.0 Hz, 1 H), 7.63 (dd, $J$ = 8.6 Hz, $J$ = 2.2 Hz, 1 H), 7.39 (d, $J$ = 8.6 Hz, 1 H), 3.86 (s, 3 H). **$^{13}$C NMR** (100 MHz, DMSO-$d_6$): δ 162.6, 154.4, 149.6, 148.4, 138.6, 138.2, 129.6, 127.1, 122.4, 115.4, 112.8, 105.1, 55.9.

# References

(1) Gottlieb, H. E.; Kotlyar, V.; Nudelman, A. *J. Org.Chem.* **1997**, *62*, 7512-7515.

(2) Fulmer, G. R.; Miller, A. J. M.; Sherden, N. H.; Gottlieb, H. E.; Nudelman, A.; Stoltz, B. M.; Bercaw, J. E.; Goldberg, K. I. *Organometallics* **2010**, *9*, 2176-2179.

(3) Hrobarikova, V.; Hrobarik, P.; Gajdos, P.; Fitilis, I.; Fakis, M.; Persephonis, P.; Zahradnik, P. *J. Org. Chem.* **2010**, *75*, 3053-3068.

(4) Obolda, A.; Peng, Q.; He, C.; Zhang, T.; Ren, J.; Ma, H.; Shuai, Z.; Li, F. *Adv. Mater.* **2016**, *28*, 4740-4746.

(5) Bair, K. W.; Baumeister, T. R.; Dragovich, P.; Zak, M.; Zhao, G.; Zheng, X. (Genentech, Inc., U.S.A.; Forma TM, LLC.) US Patent 2014074715, May 15, 2014.

(6) Lei, X.; Jalla, A.; Abou Shama, M. A.; Stafford, J. M.; Cao, B. *Synthesis* **2015**, *47*, 2578-2585.

(7) Kawano, Y.; Togo, H. *Tetrahedron* **2009**, *65*, 6251-6256.

(8) Yang, T.; Liu, Q.; Cheng, Y.; Cai, W.; Ma, Y.; Yang, L.; Wu, Q.; Orband-Miller, L. A.; Zhou, L.; Xiang, Z.; Huxdorf, M.; Zhang, W.; Zhang, J.; Xiang, J.-N.; Leung, S.; Qiu, Y.; Zhong, Z.; Elliott, J.; Lin, X.; Wang, Y. *ACS Med. Chem. Lett.* **2014**, *5*, 65-68.

(9) Thomson, S. P.; Davies, R. T.; Allanson, N. M.; Kuvshinov, A.; Davies, G. M.; Edwards, P. N. (F2G Ltd., U.K.) US Patent 2006123145, 23 November 23, 2006.

(10) Hattori, K.; Sajiki, H.; Hirota, K. *Tetrahedron* **2001**, *57*, 2109-2114.

(11) Kawashita, Y.; Nakamichi, N.; Kawabata, H.; Hayashi, M. *Org. Lett.* **2003**, *5*, 3713-3715.

(12) Lanning, M. E.; Yu, W.; Yap, J. L.; Chauhan, J.; Chen, L.; Whiting, E.; Pidugu, L. S.; Atkinson, T.; Bailey, H.; Li, W.; Roth, B. M.; Hynicka, L.; Chesko, K.; Toth, E. A.; Shapiro, P.; MacKerell, A. D. Jr.; Wilder, P. T.; Fletcher, S. *Eur. J. Med. Chem.* **2016**, *113*, 273-292.

(13) Xu, X.-H.; Liu, G.-K.; Azuma, A.; Tokunaga, E.; Shibata, N. *Org. Lett.* **2011**, *13*, 4854-4857.

(14) Cheung, C. W.; Buchwald, B. L. *J. Org. Chem.* **2012**, *77*, 7526-7537.

(15) Liu, J.; Liu, Q.; Yi, H.; Qin, C.; Bai, R.; Qi, X.; Lan, Y.; Lei, A. *Angew. Chem. Int. Ed.* **2014**, *53*, 502-506.

(16) Cheung, C. W.; Ploeger, M. L.; Hu, X. *Nat. Commun.* **2017**, *8*, 14878.

(17) Engers, D. W.; Niswender, C. M.; Weaver, C. D.; Jadhav, S.; Menon, U. N.; Rocio Zamorano, R.; Conn, P. J.; Lindsley, C. W.; Hopkins, C. R. *J. Med. Chem.* **2009**, *52*, 4115-4118.

**$^{1}$H and $^{13}$C NMR of Fluoro-3-(4-nitrophenyl)pyridine (S1).**

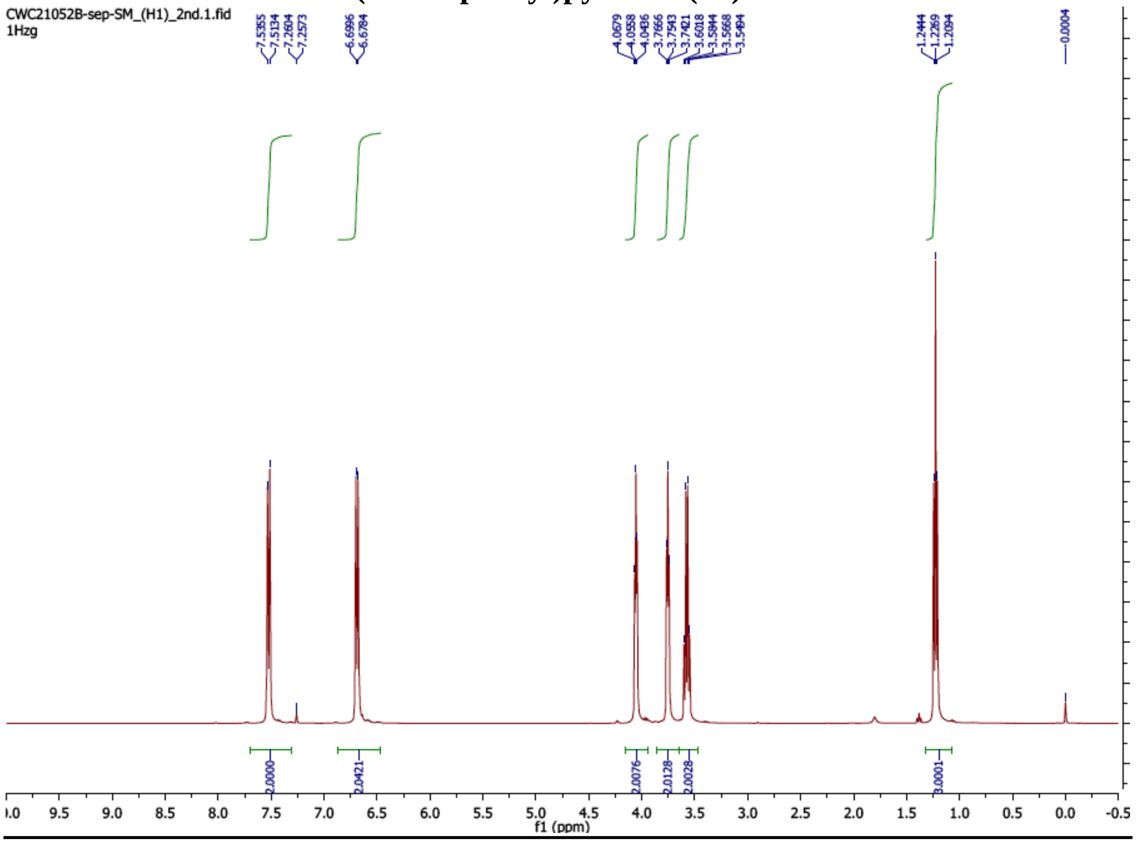

$^{1}$H and $^{13}$C NMR of 2-Fluoro-3-(4-nitrophenyl)pyridine (S2).

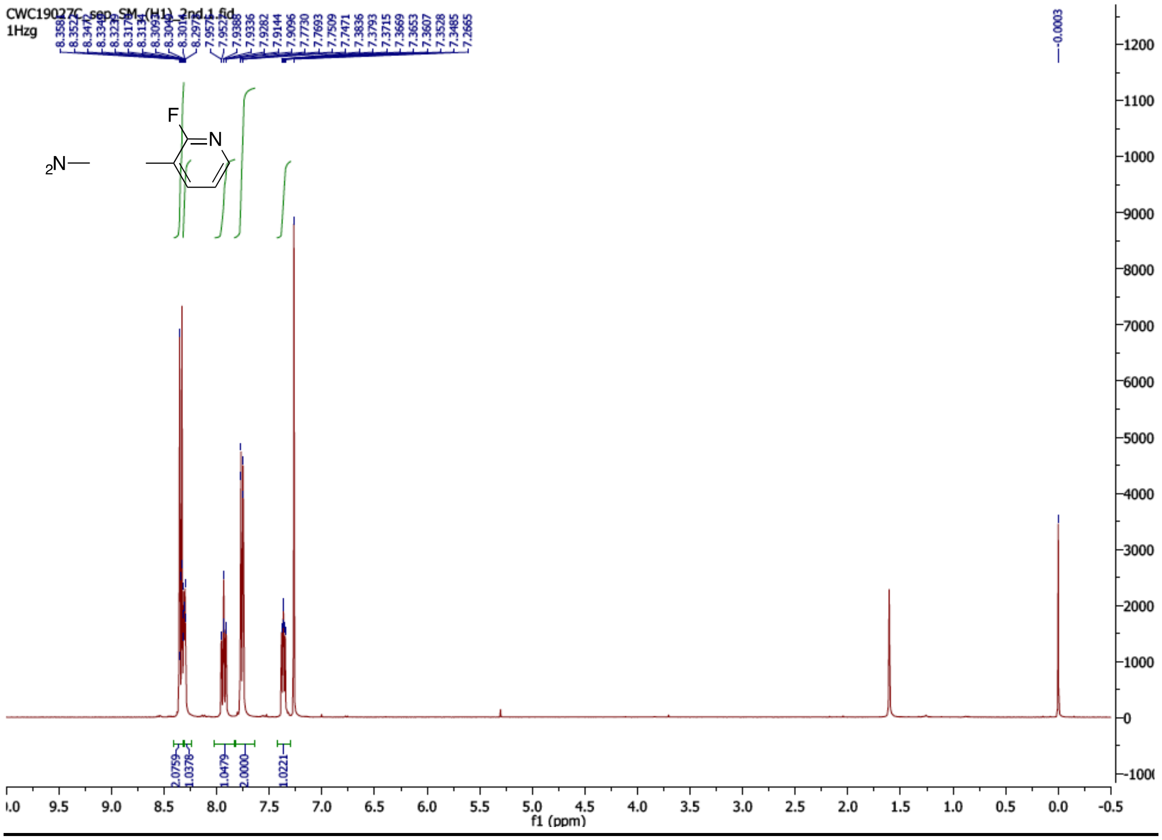

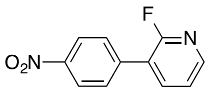

# $^{1}$H and $^{13}$C NMR of *N*-(4-(methylthio)phenyl)benzamide (3a).

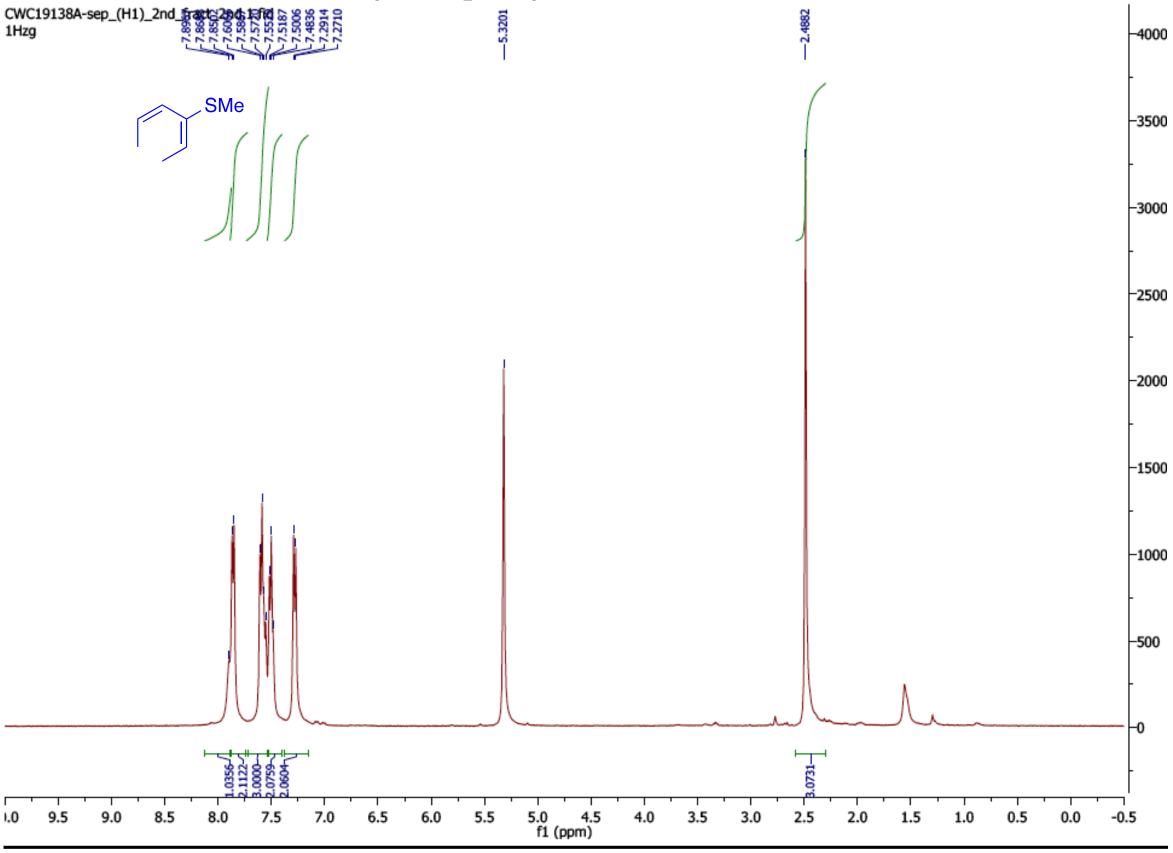


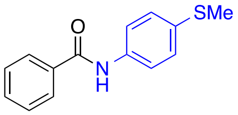

$^{1}H$ and $^{13}C$ NMR of *N*-(3-fluoro-4-methylphenyl)benzamide (3b).

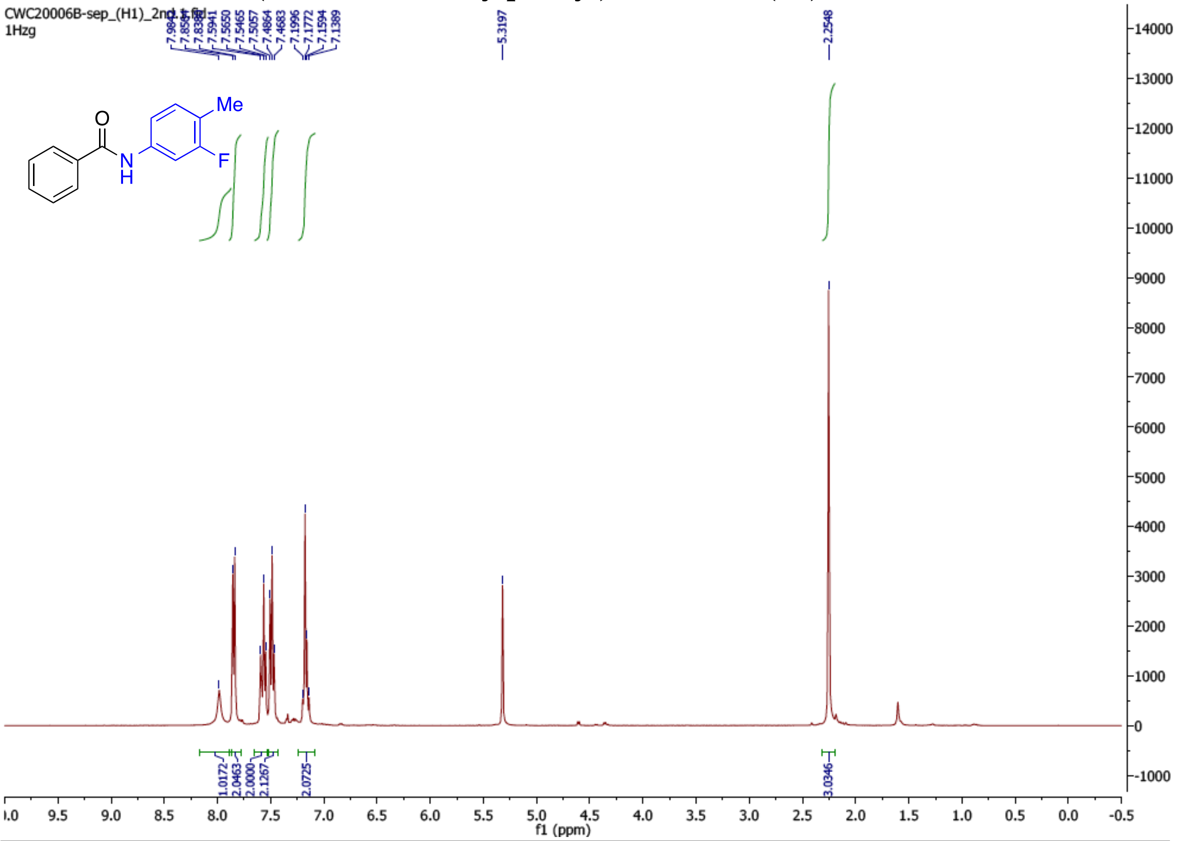


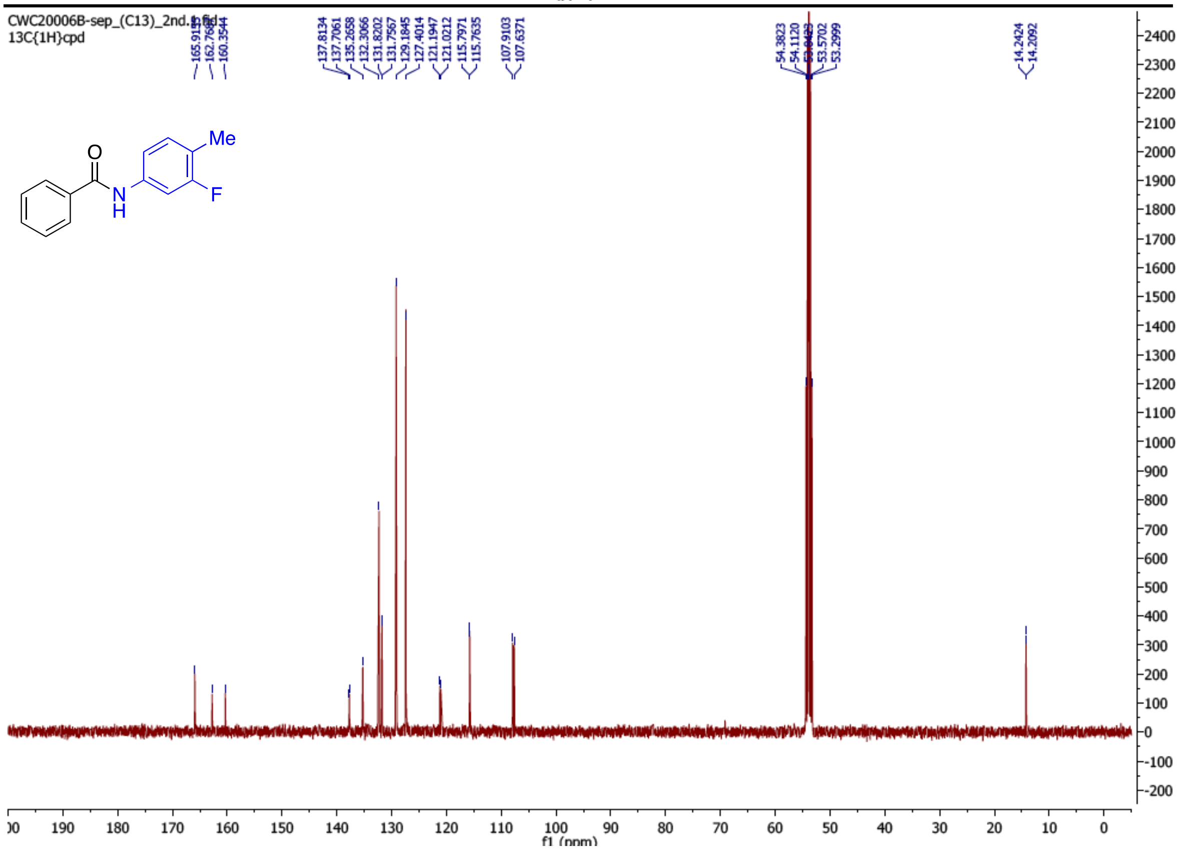

**$^{1}$H and $^{13}$C NMR of *N*-(3-chloro-4-methoxyphenyl)benzamide (3c).**

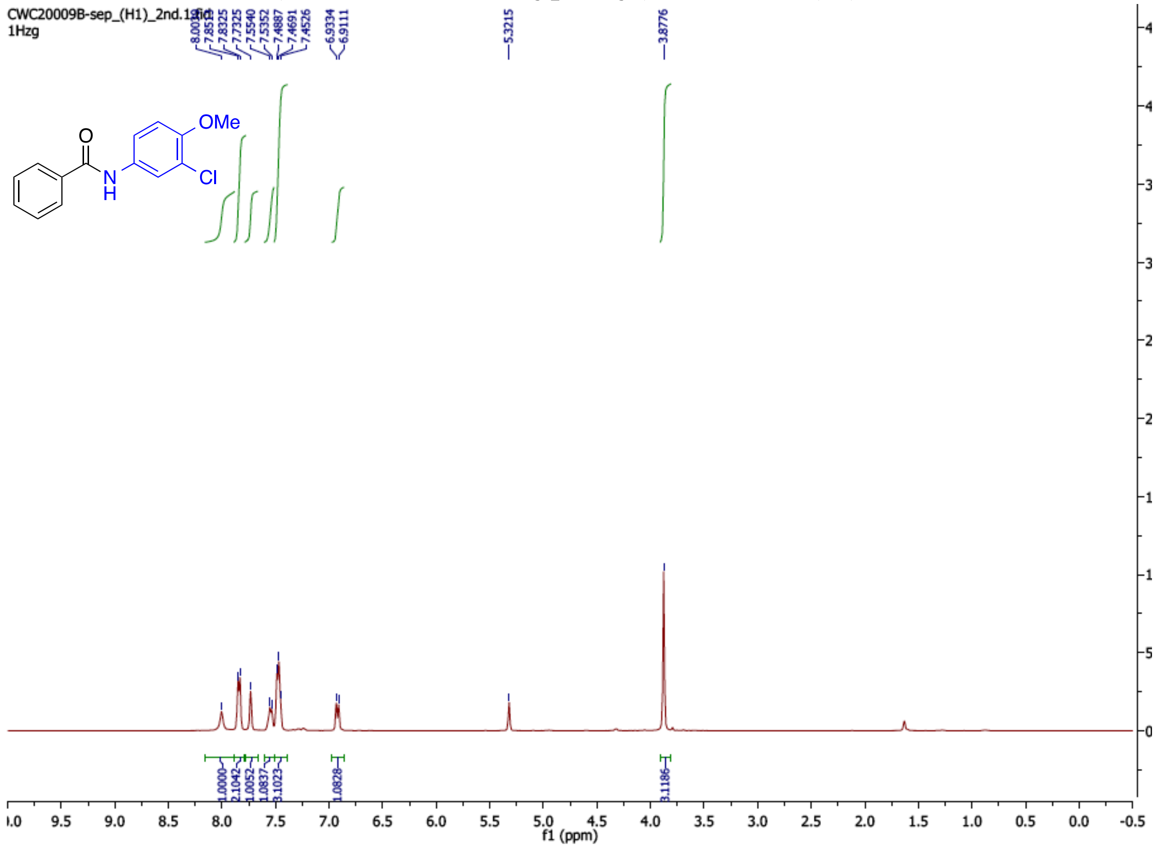


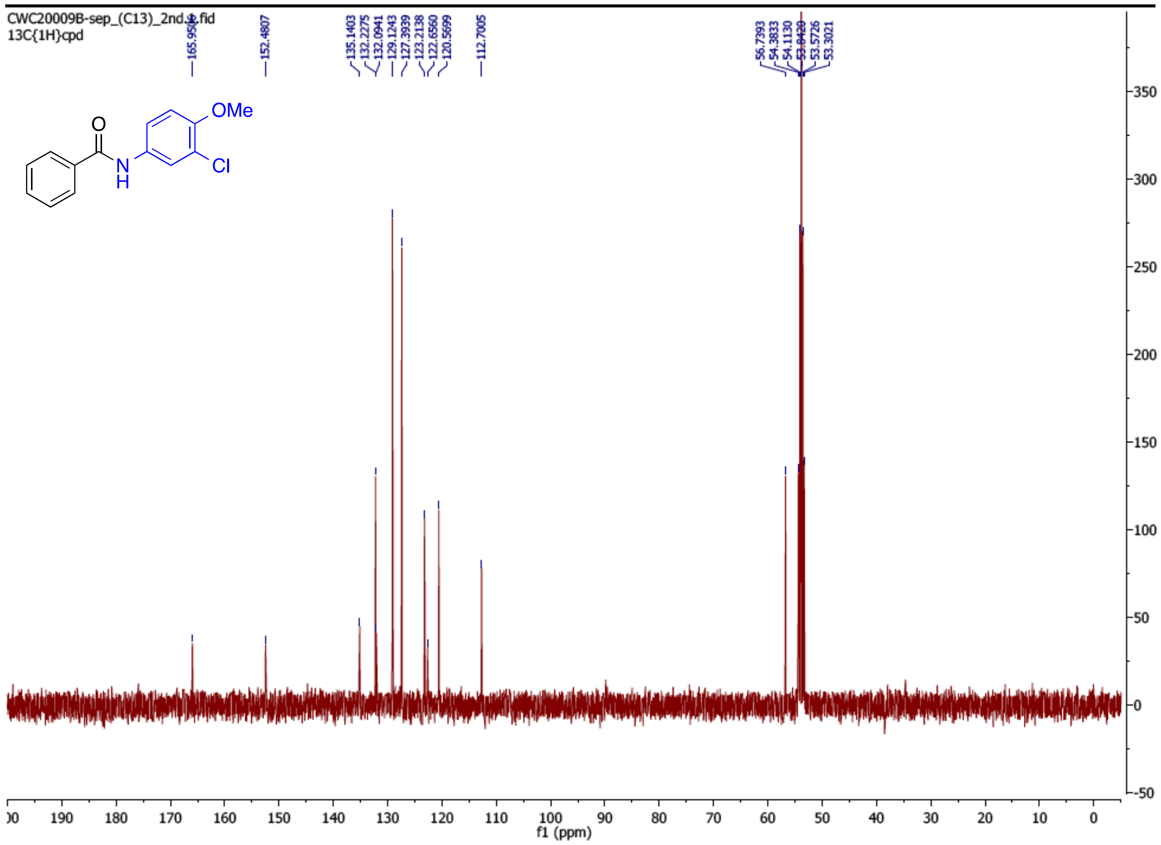

$^{1}H$ and $^{13}C$ NMR of *N*-(3-bromo-4-methylphenyl)benzamide (3d).

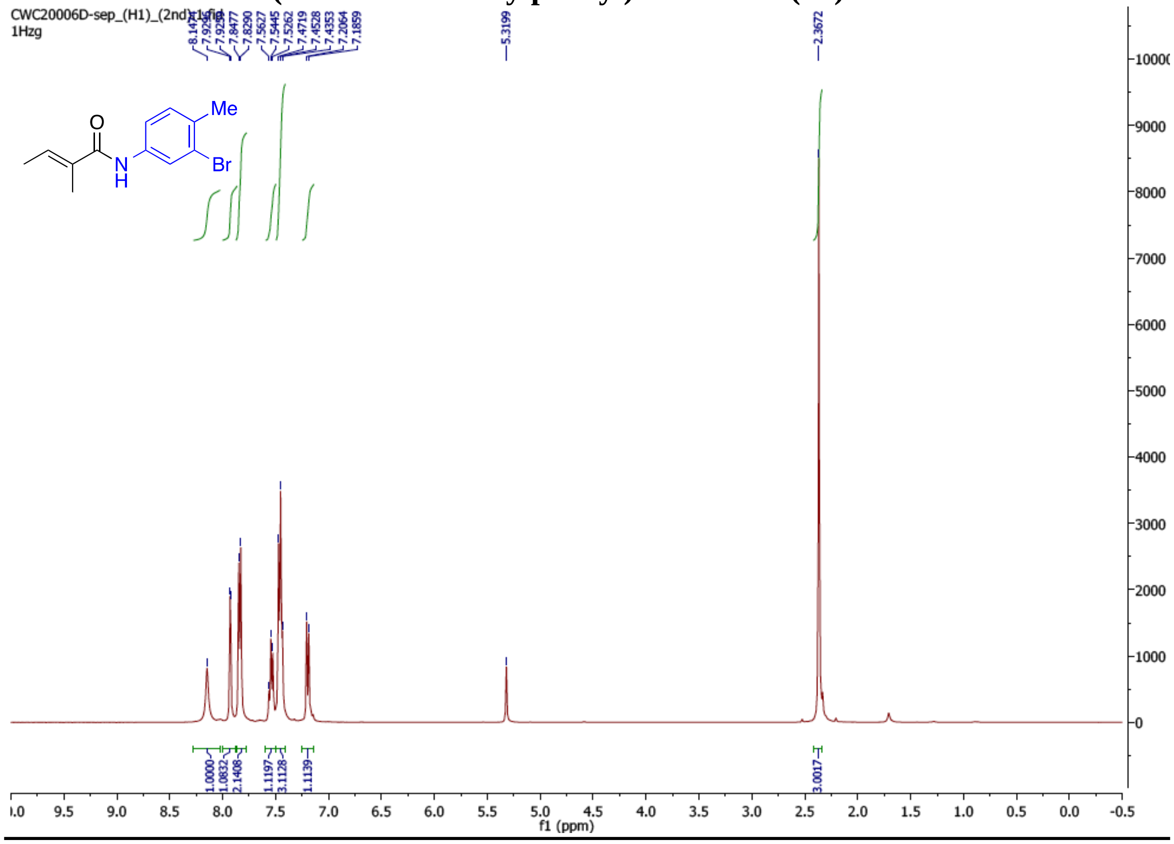


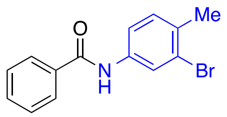

**$^{1}$H and $^{13}$C NMR of *N*-(4-(2-fluoropyridin-3-yl)phenyl)benzamide (3e).**

O

grease H

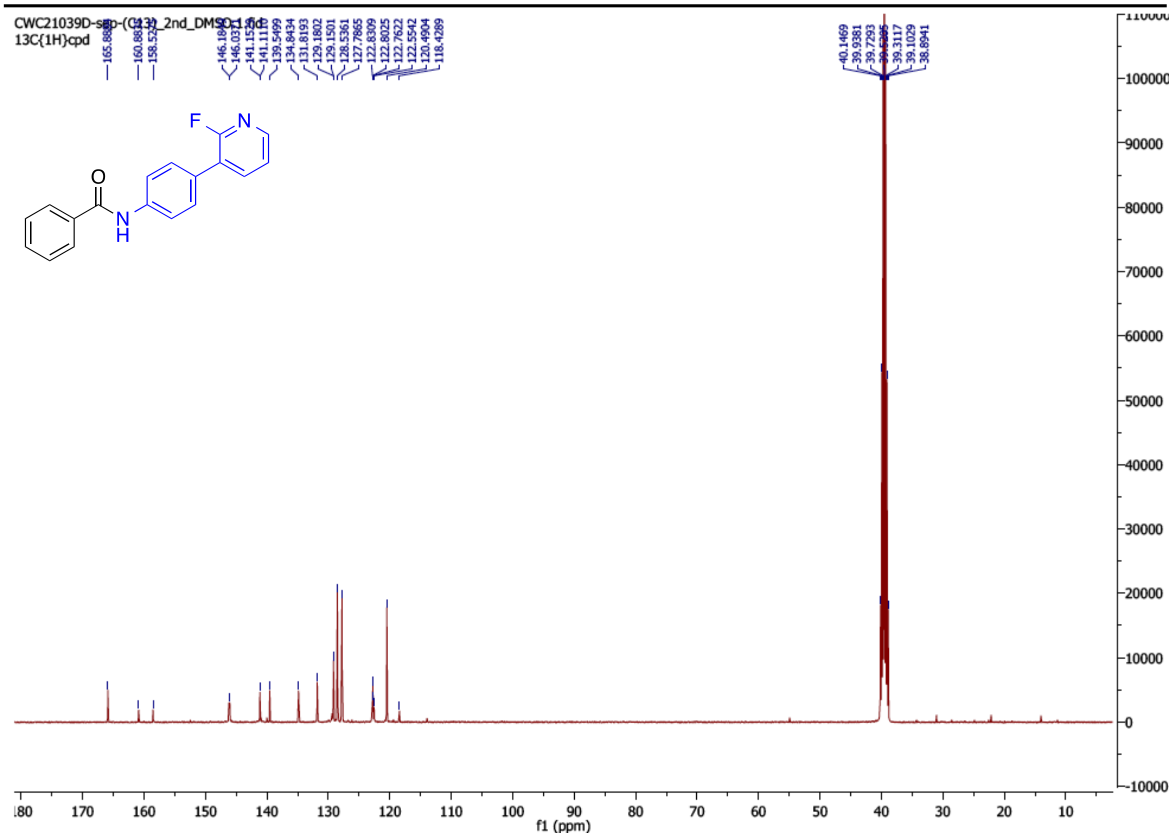

**$^{1}$H and $^{13}$C NMR of *N*-(4-(1*H*-pyrrol-1-yl)phenyl)benzamide (3f).**

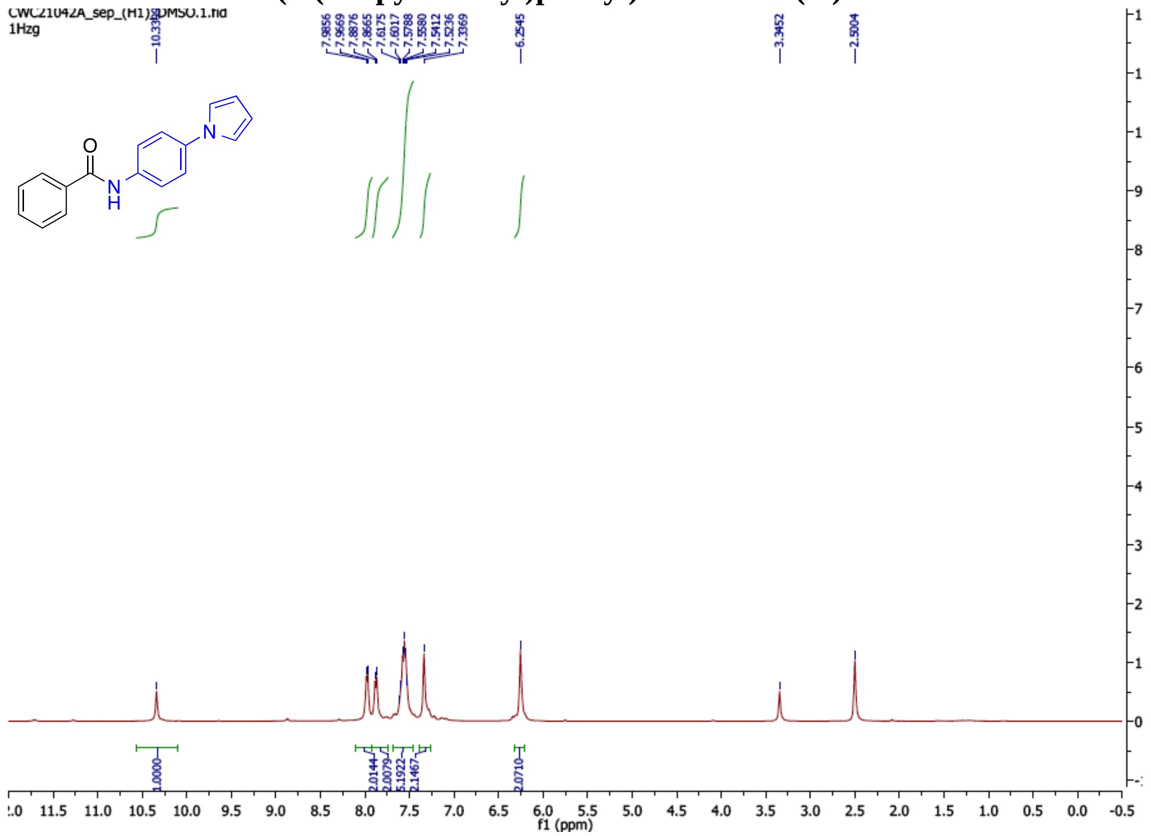

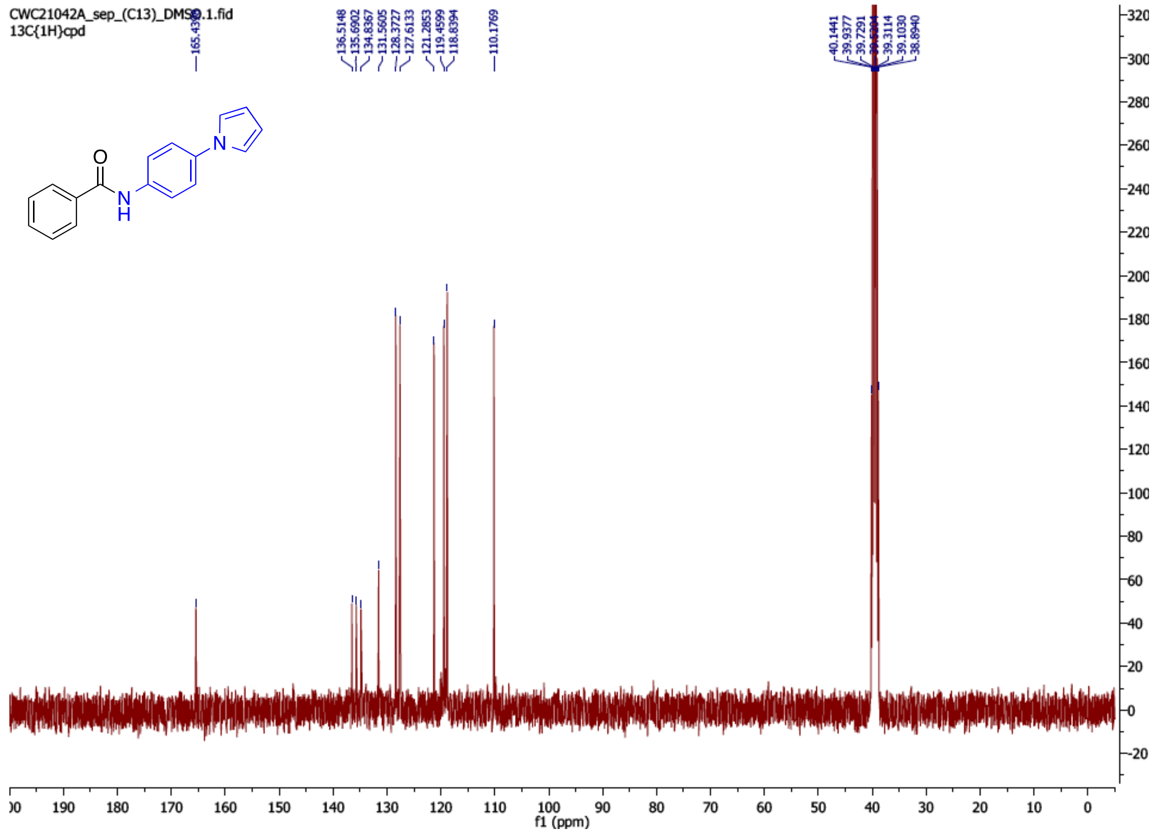

**$^{1}$H and $^{13}$C NMR of *N*-(1*H*-indol-6-yl)benzamide (3g).**

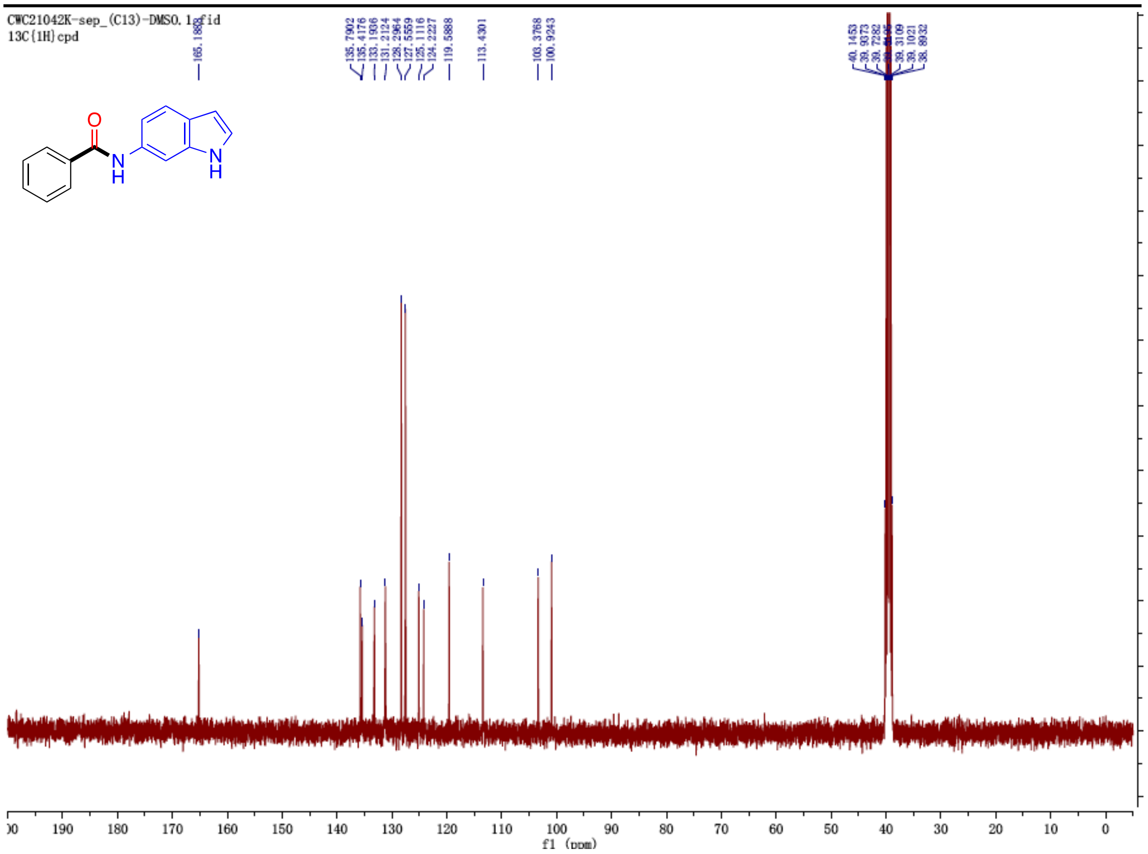

**$^{1}$H and $^{13}$C NMR of *N*-(3-cyano-4-methylphenyl)-4-methoxybenzamide (3h).**

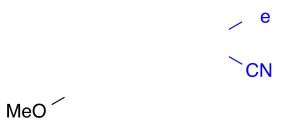


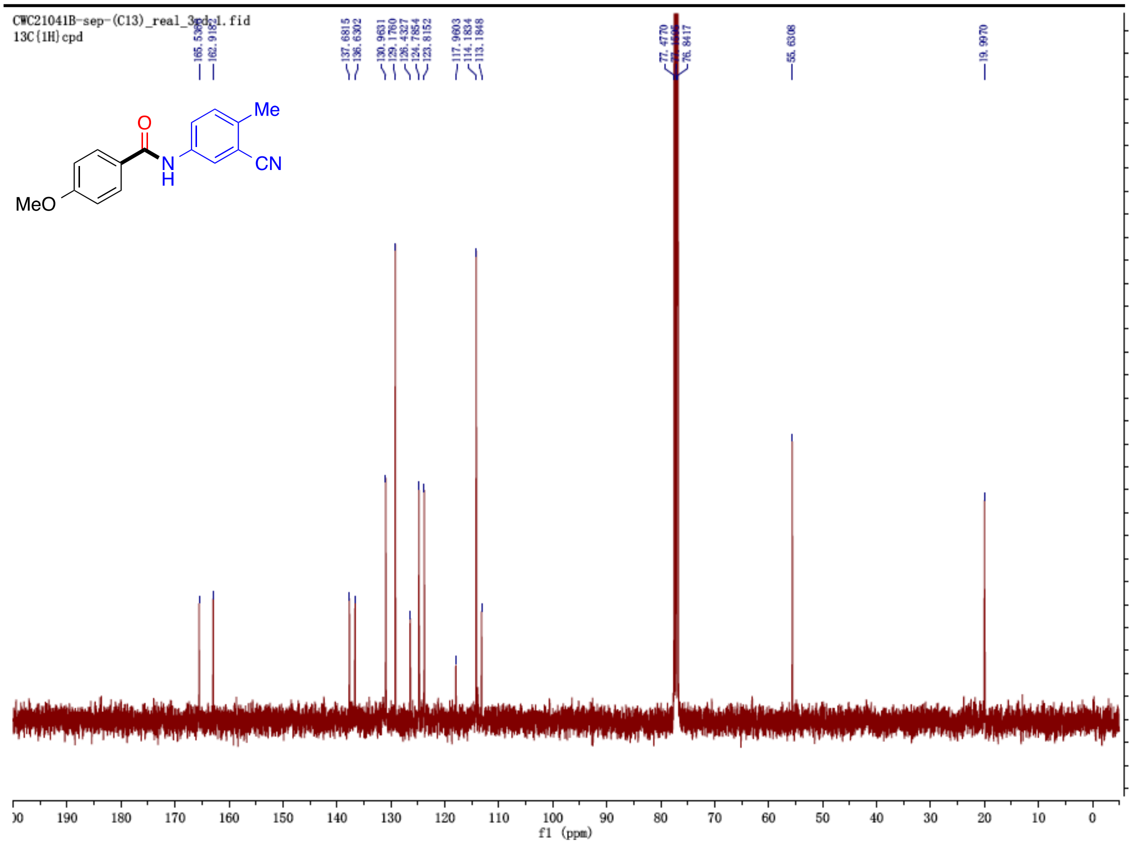

$^{1}$H and $^{13}$C NMR of *N*-(2,3-dihydro-1*H*-inden-4-yl)-4-methoxybenzamide (3i).

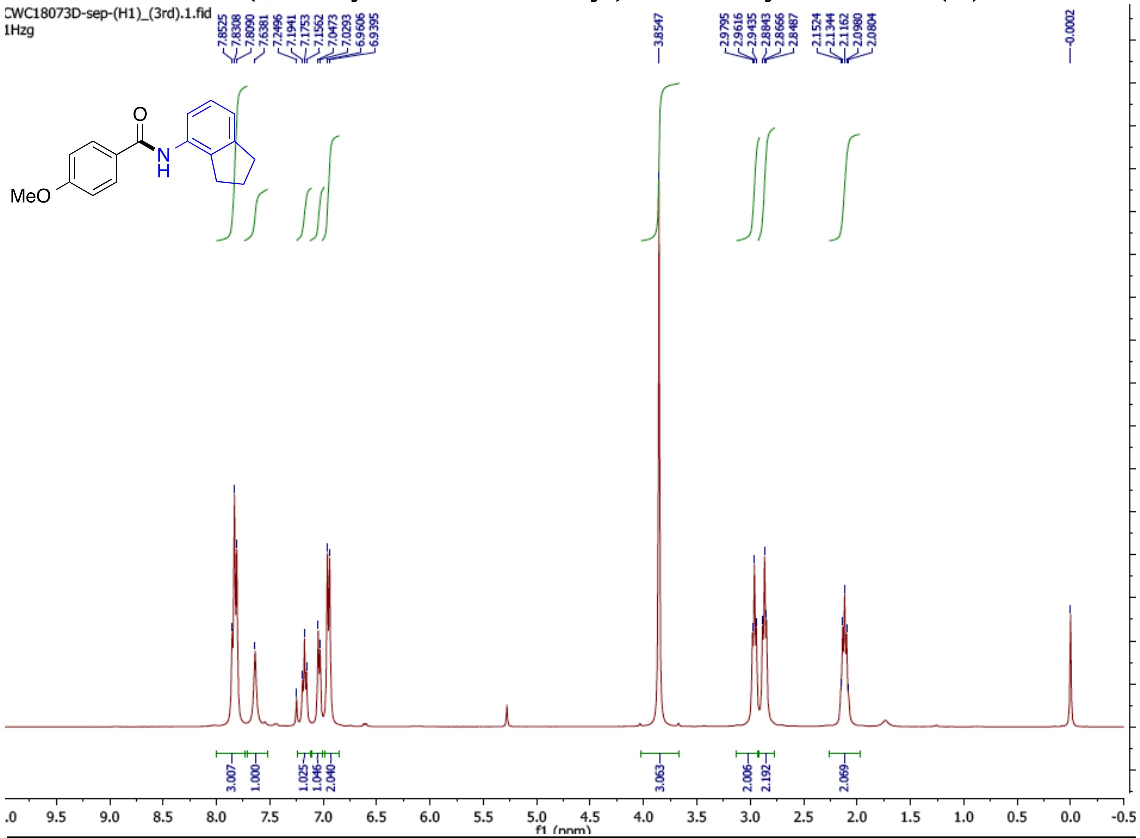


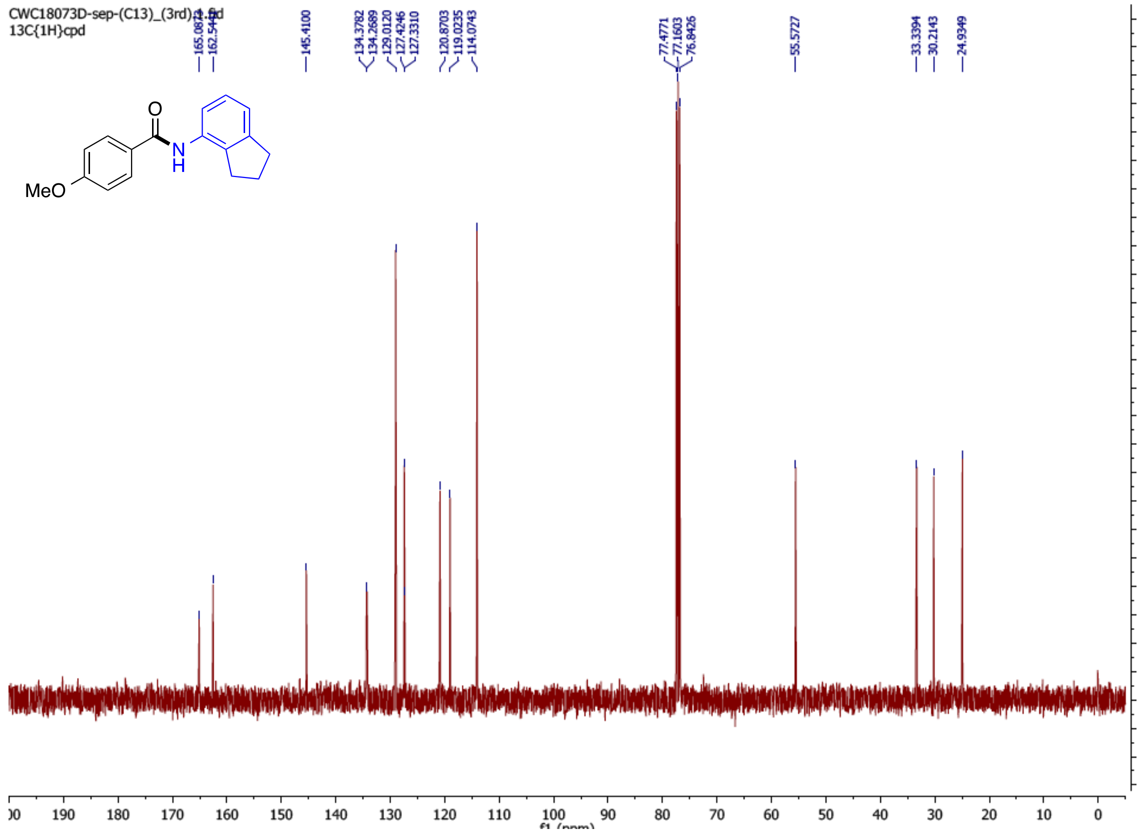

**$^1$H and $^{13}$C NMR of *N*-(2,4-dimethylphenyl)-4-methoxybenzamide (3j).**

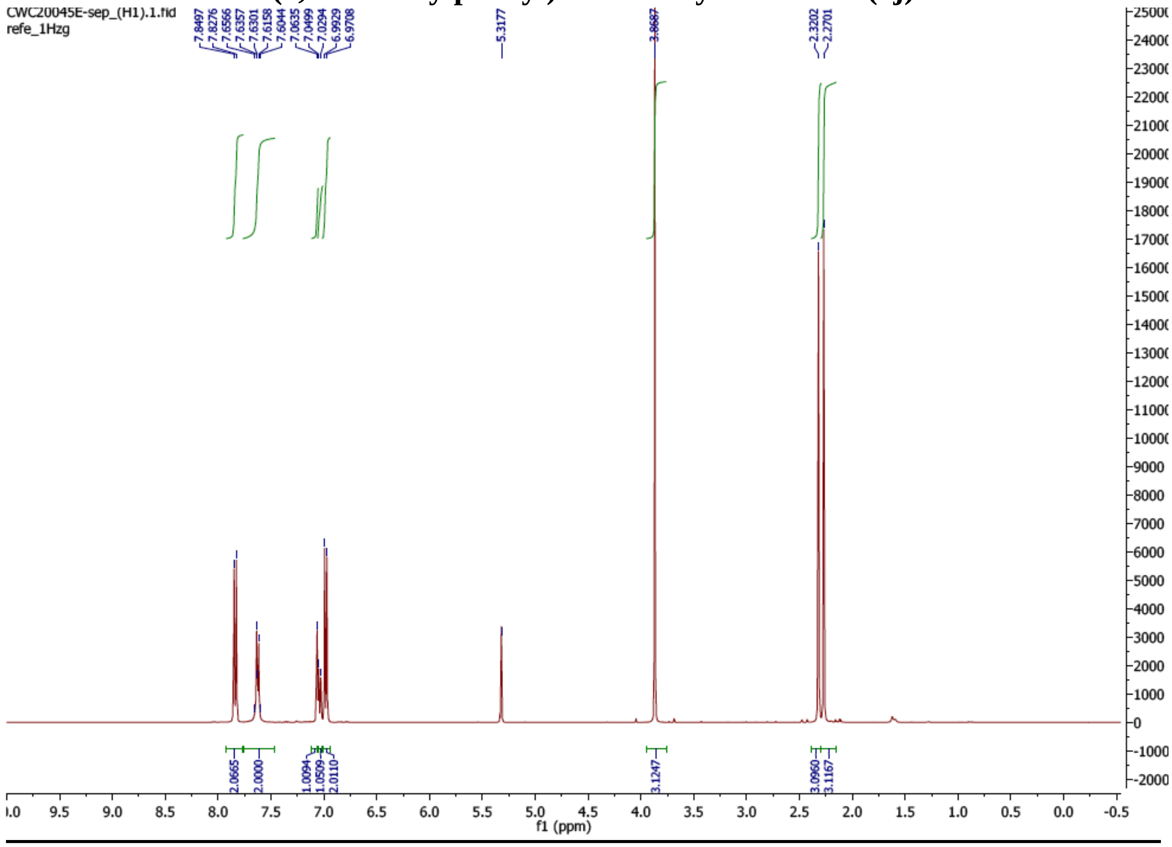


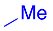

**$^{1}$H and $^{13}$C NMR of 4-methoxy-*N*-(6-methoxypyridin-3-yl)benzamide (3k).**

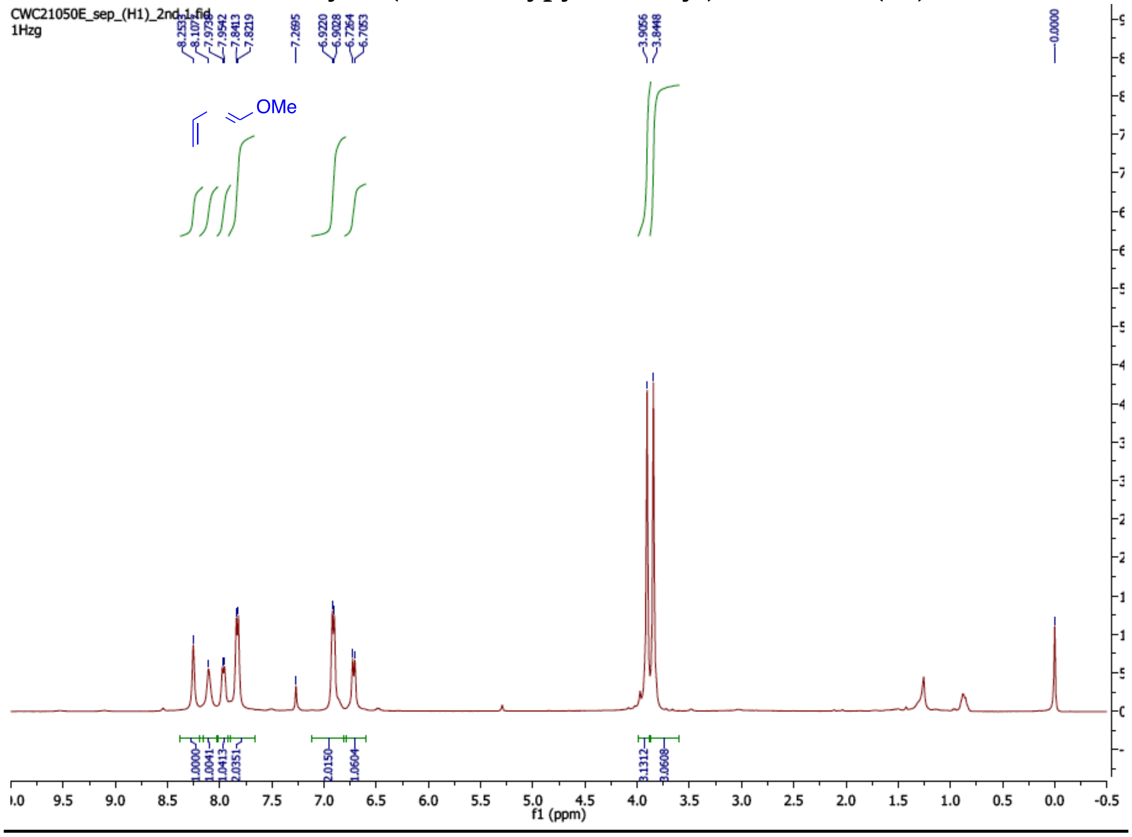


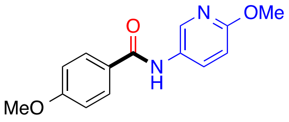

**$^{1}$H and $^{13}$C NMR of 4-methoxy-*N*-(2-methylbenzo[*d*]thiazol-6-yl)benzamide (3l).**

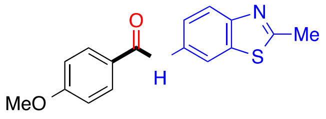


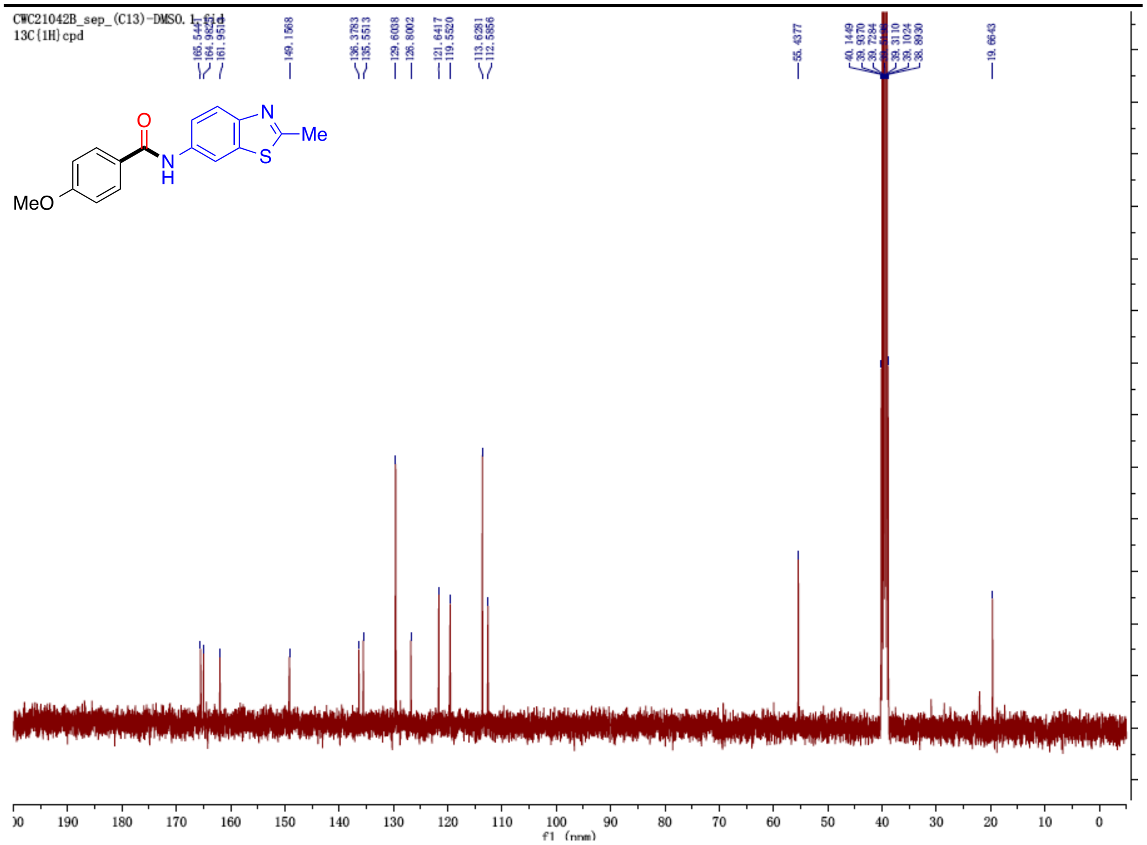

**$^{1}$H and $^{13}$C NMR of 4-methoxy-*N*-(2-oxo-2*H*-chromen-6-yl)benzamide (3m).**

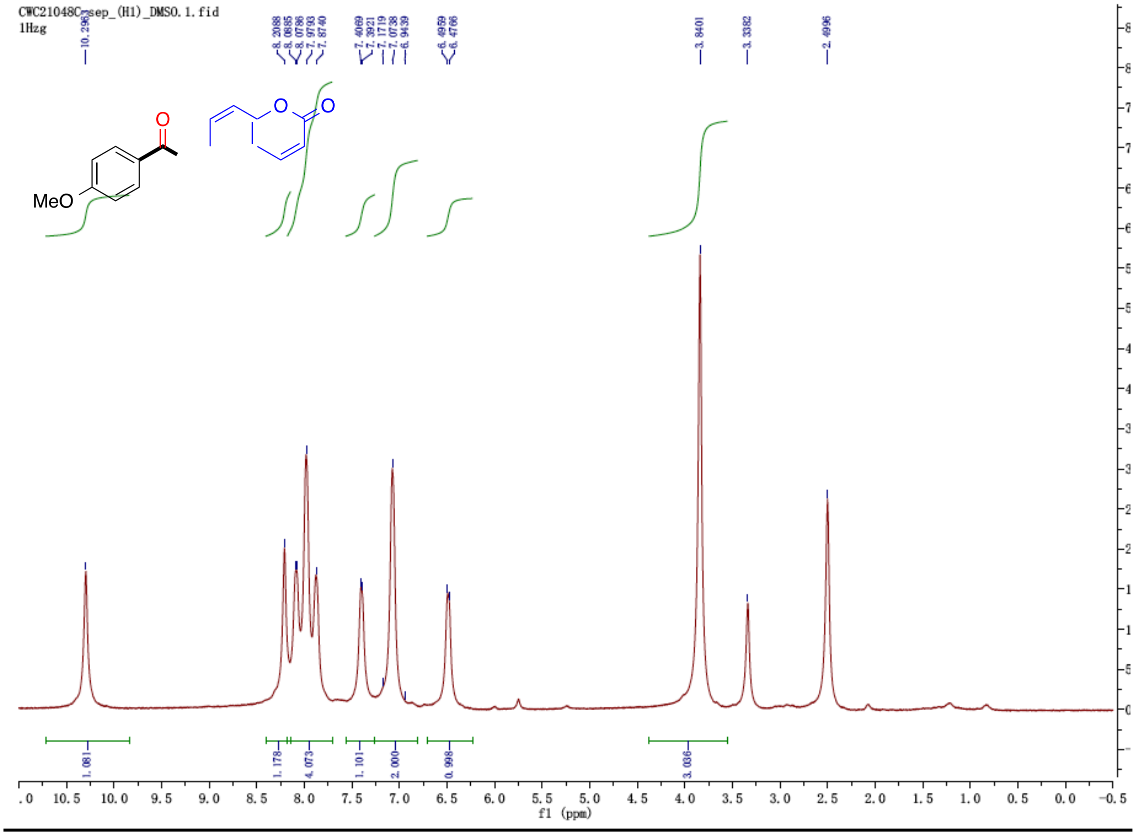


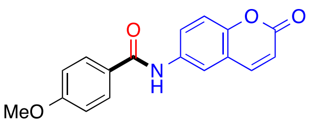

$^{1}$H and $^{13}$C NMR of (*E*)-4-(2-ethoxyethoxy)-*N*-(4-styrylphenyl)benzamide (3n).

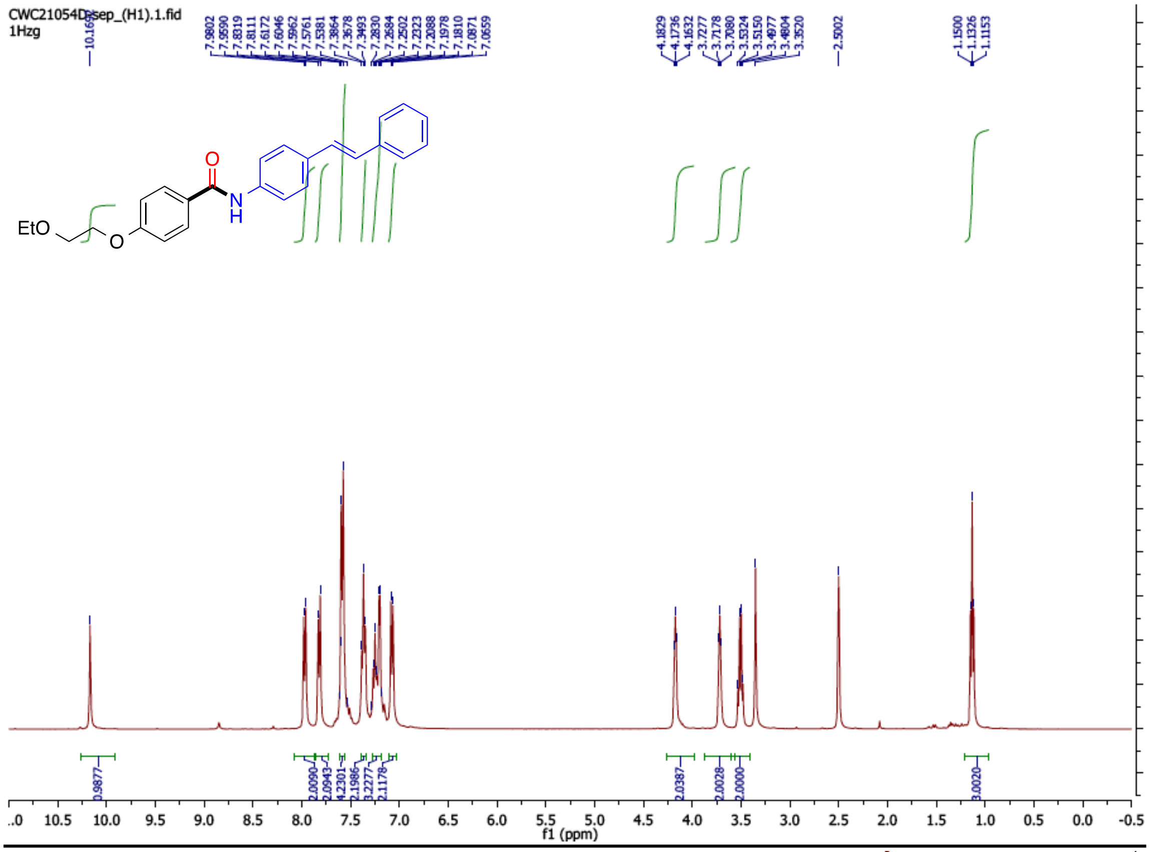

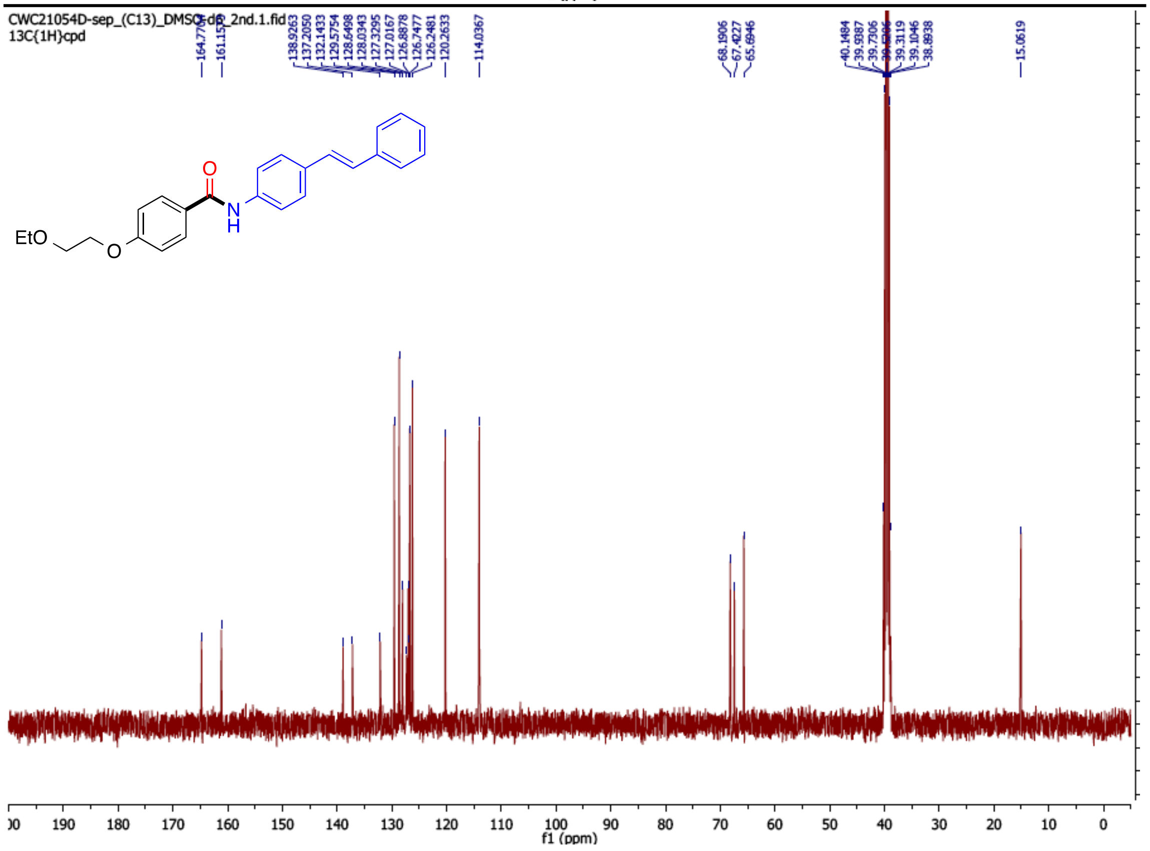

**$^{1}$H and $^{13}$C NMR of 4-(4-(*tert*-butyl)benzamido)phenyl pivalate (3o).**

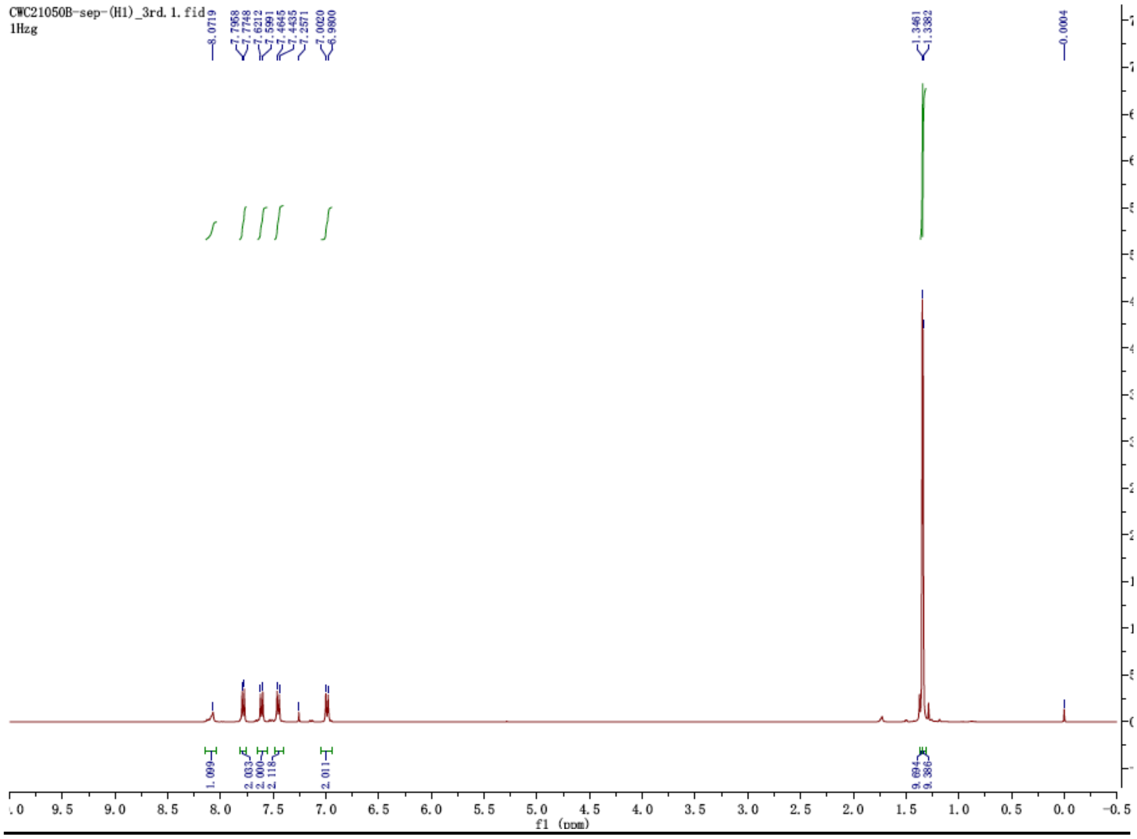


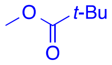

**$^{1}$H and $^{13}$C NMR of *N*-(4-(9*H*-carbazol-9-yl)phenyl)-4-methylbenzamide (3p).**

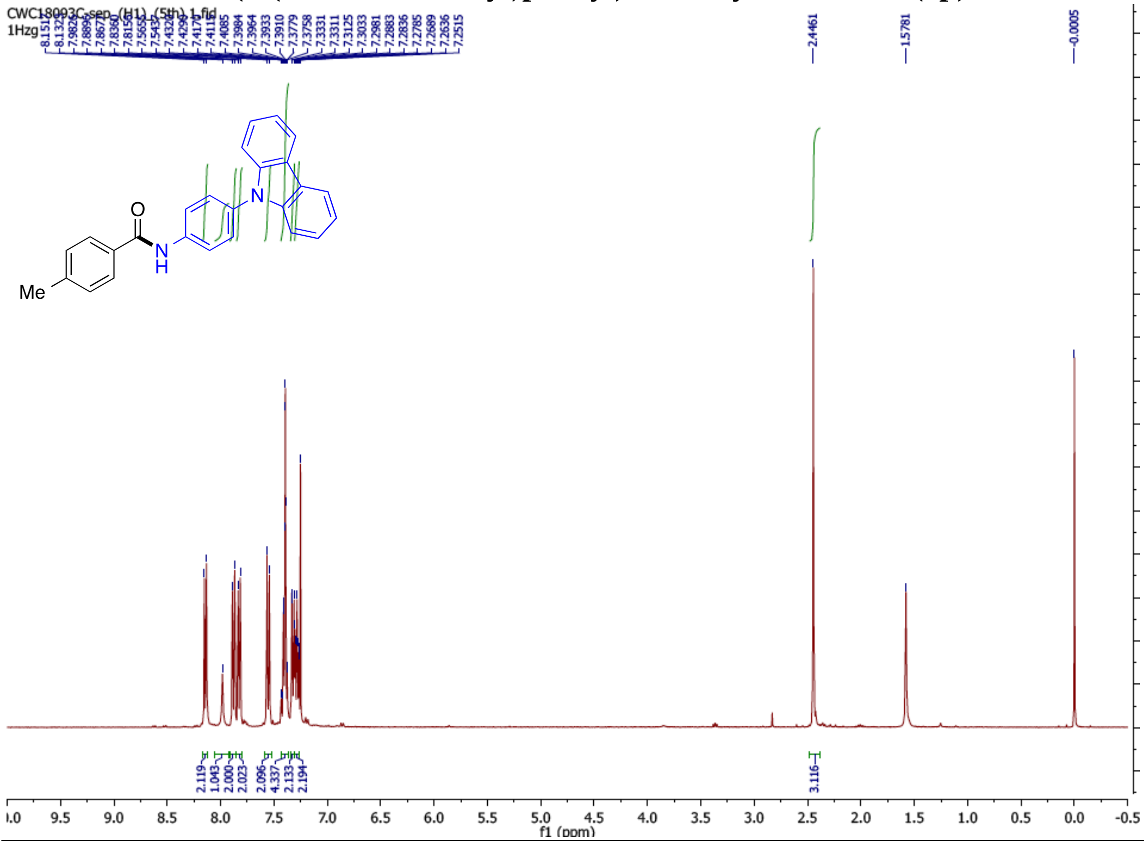

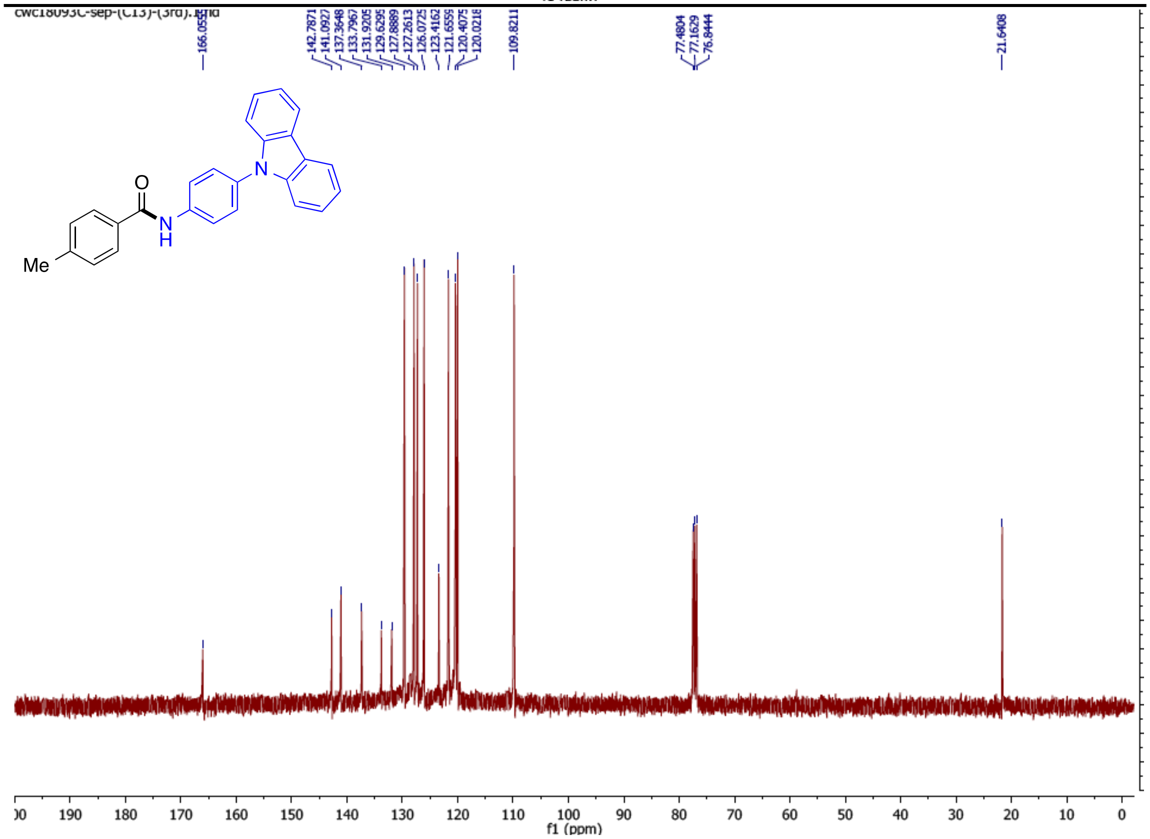

**$^{1}$H and $^{13}$C NMR of *N*-(4-(1*H*-pyrazol-1-yl)phenyl)-2-methylbenzamide (3q).**

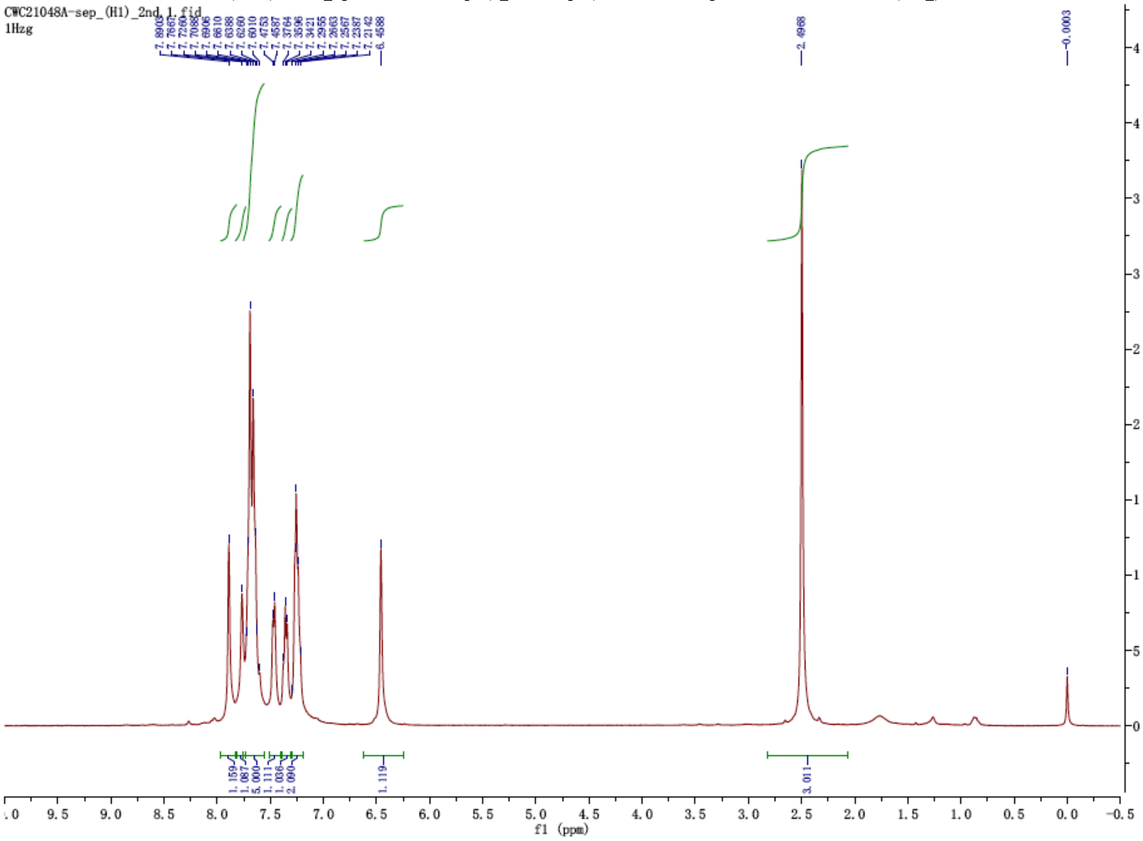

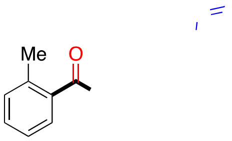

$^{1}$H and $^{13}$C NMR of *N*-(4-(*tert*-butyl)phenyl)-4-chlorobenzamide (3r).

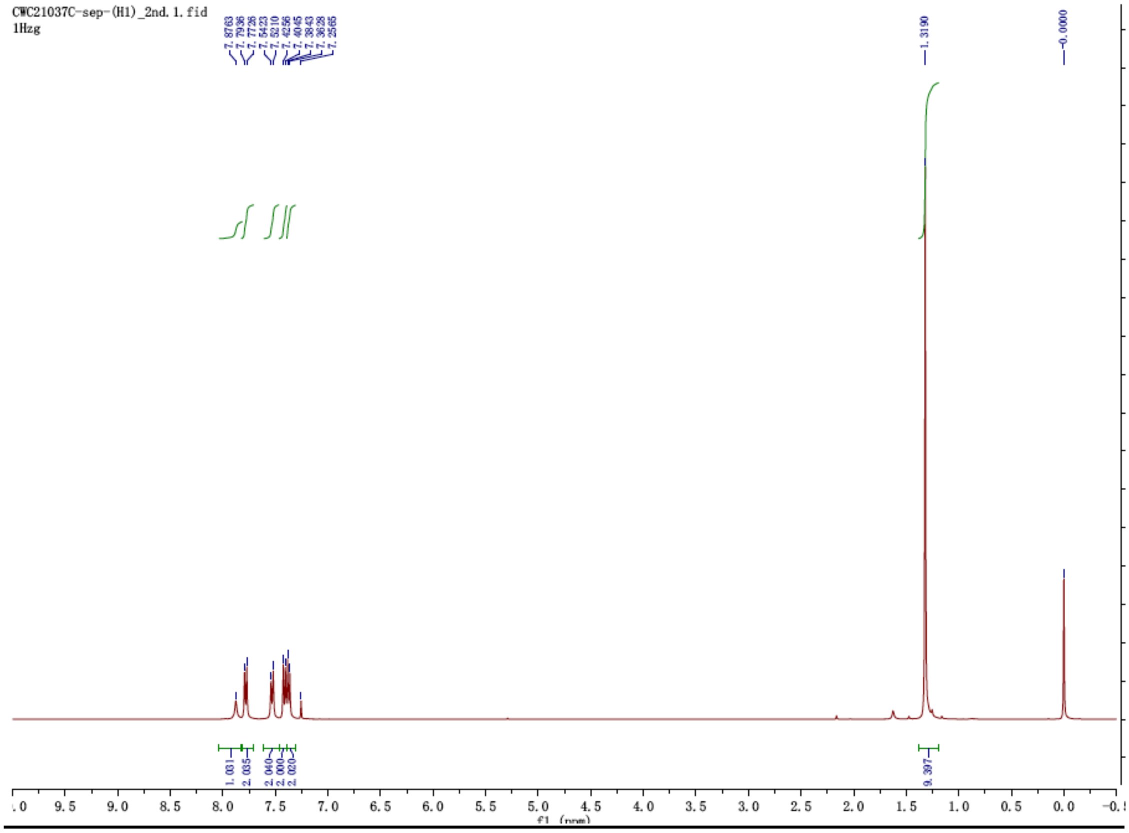

**$^{1}H$ and $^{13}C$ NMR of *N*-(4-(*tert*-butyl)phenyl)-4-fluorobenzamide (3s).**

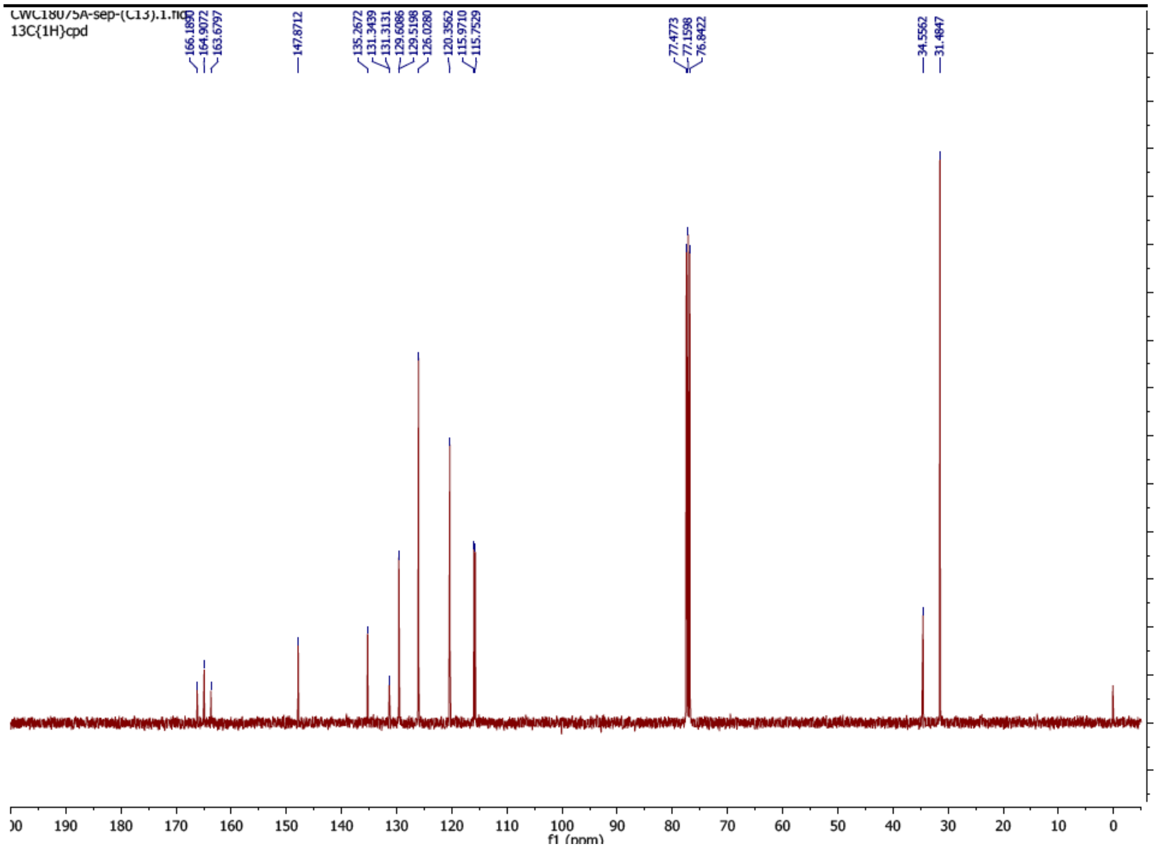

**$^{1}$H and $^{13}$C NMR of *N*-(4-(phenylthio)phenyl)-4-(trifluoromethoxy)benzamide (3t).**

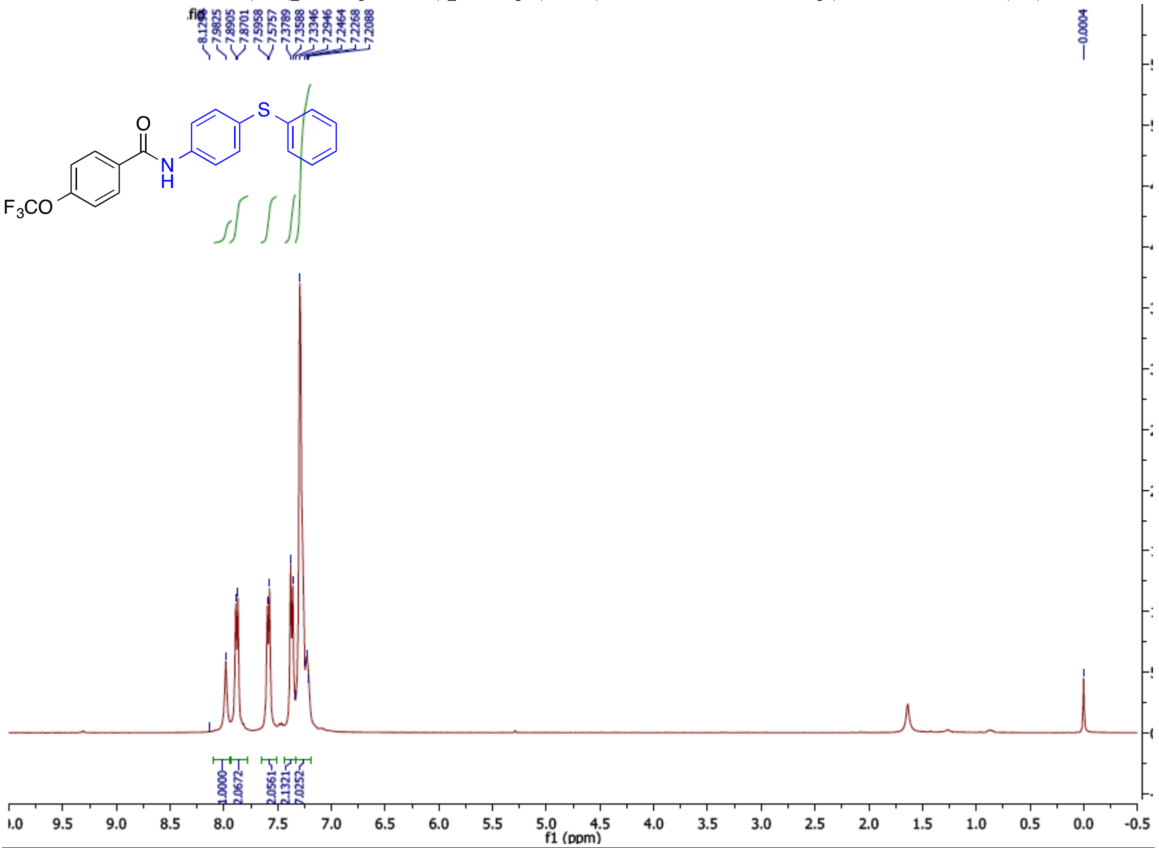

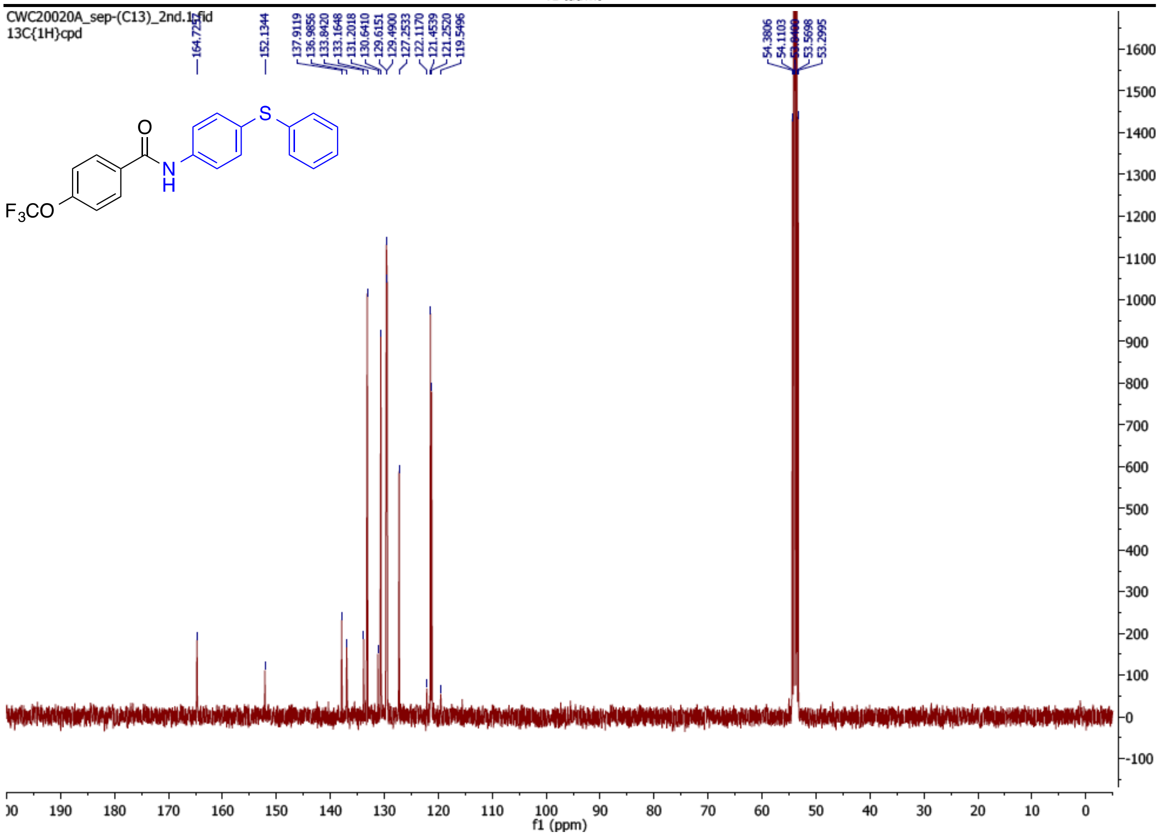

$^{1}$H and $^{13}$C NMR of *N*-(4-(*tert*-butyl)phenyl)-4-(trifluoromethyl)benzamide (3u).

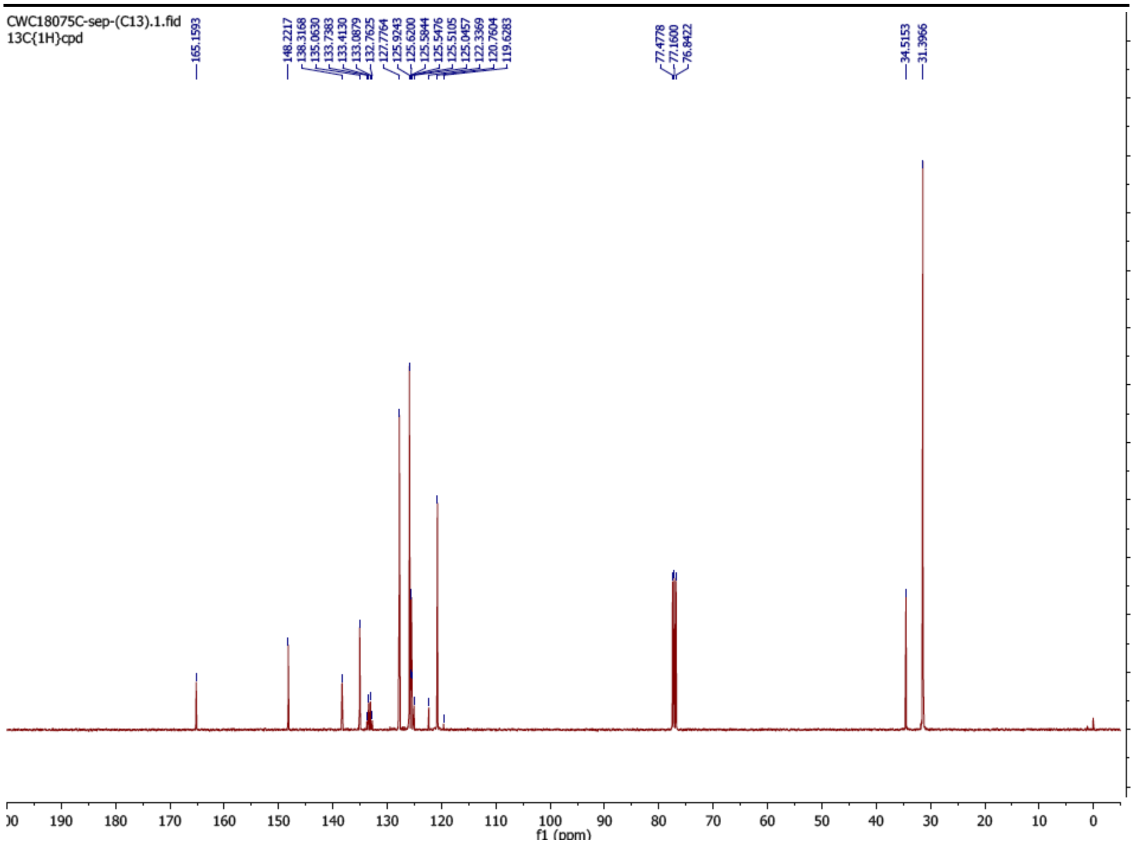

$^{1}$H and $^{13}$C NMR of *N*-(4-(*tert*-butyl)phenyl)-4-cyanobenzamide (3v).

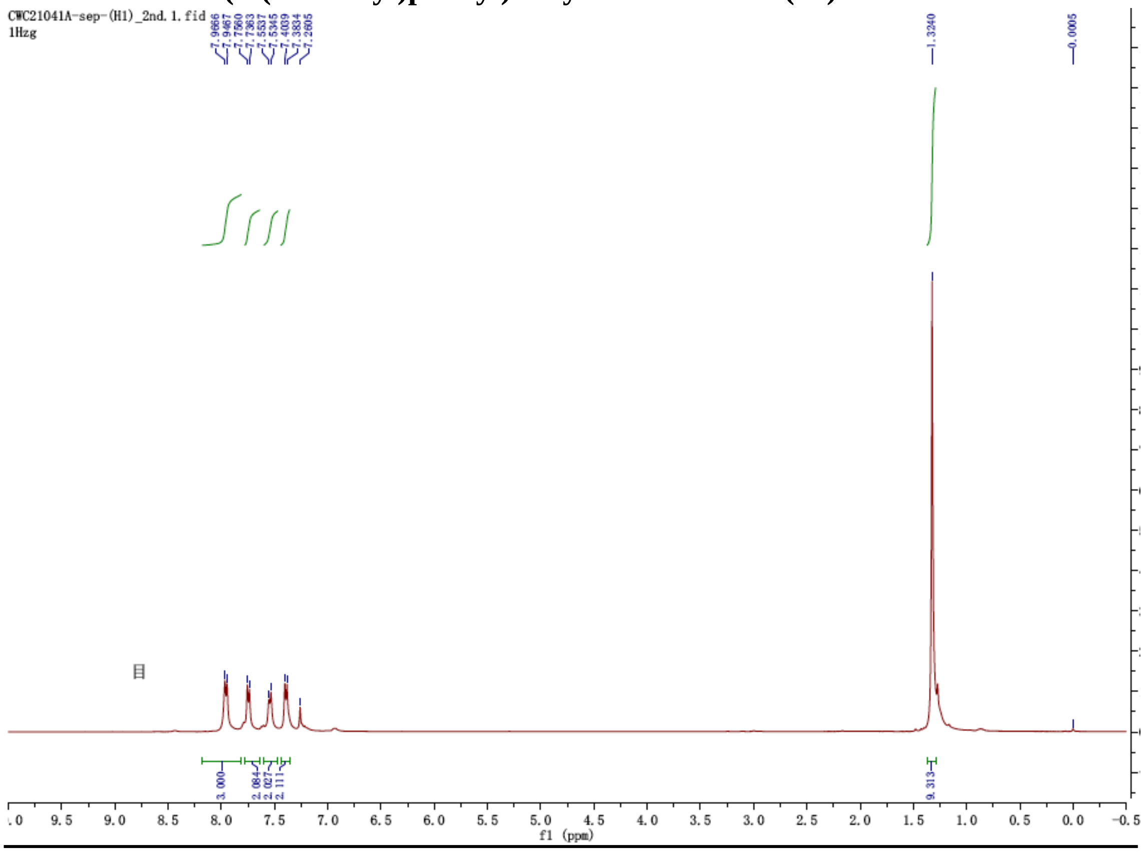

**$^{1}$H and $^{13}$C NMR of 4-(3-methoxybenzamido)phenyl 4-methylbenzenesulfonate (3w).**

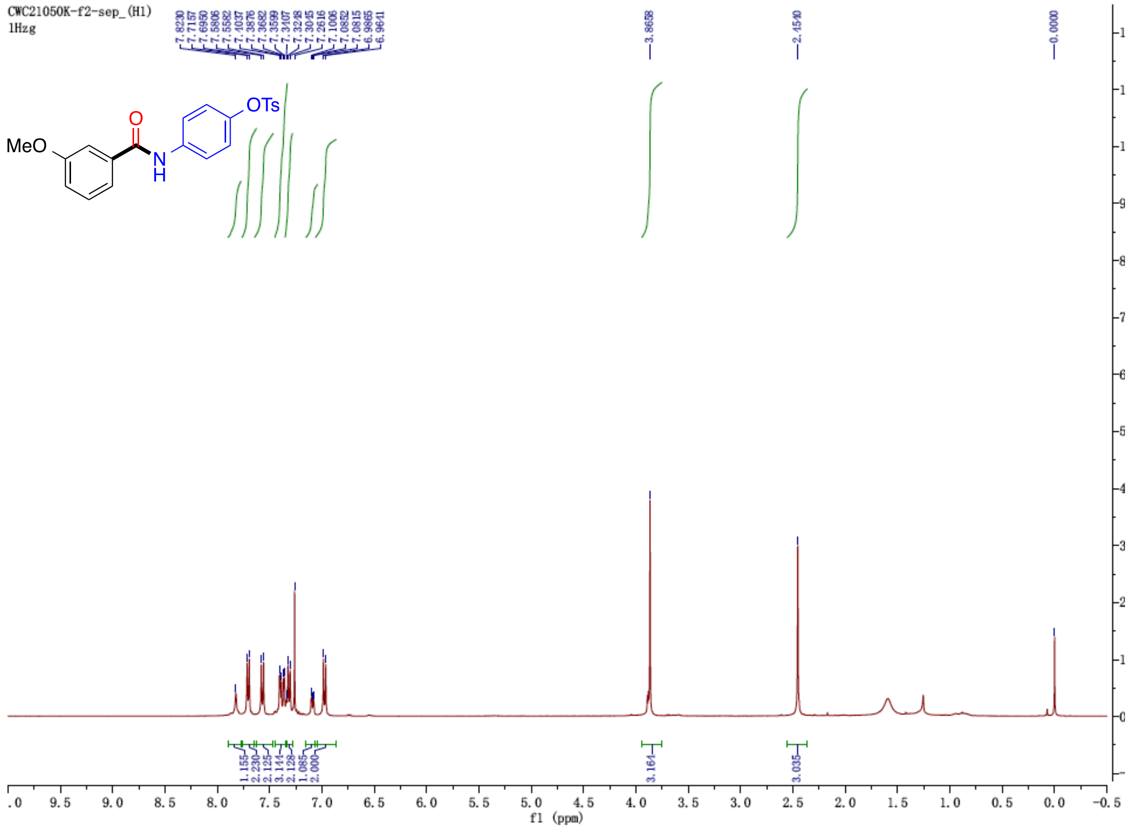

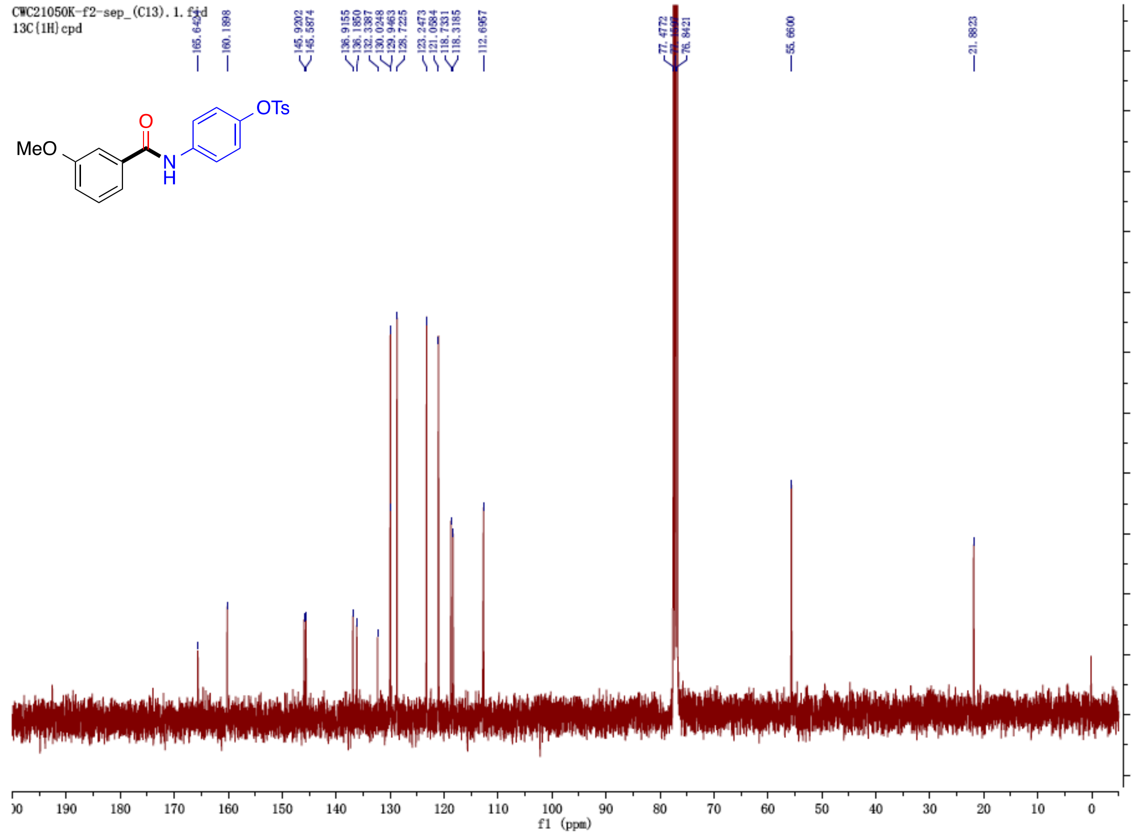

# $^{1}$H and $^{13}$C NMR of *N*-(4-(2-ethyloxazol-5-yl)phenyl)-3-methoxybenzamide (3x).

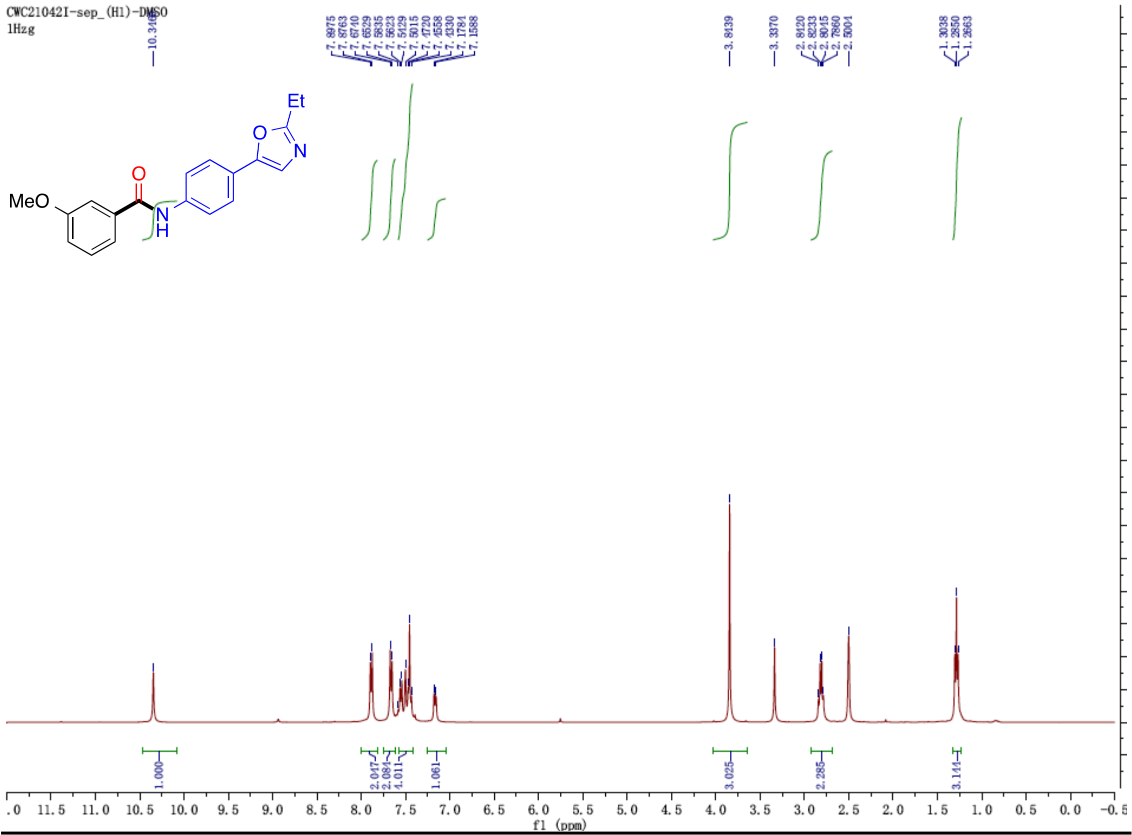

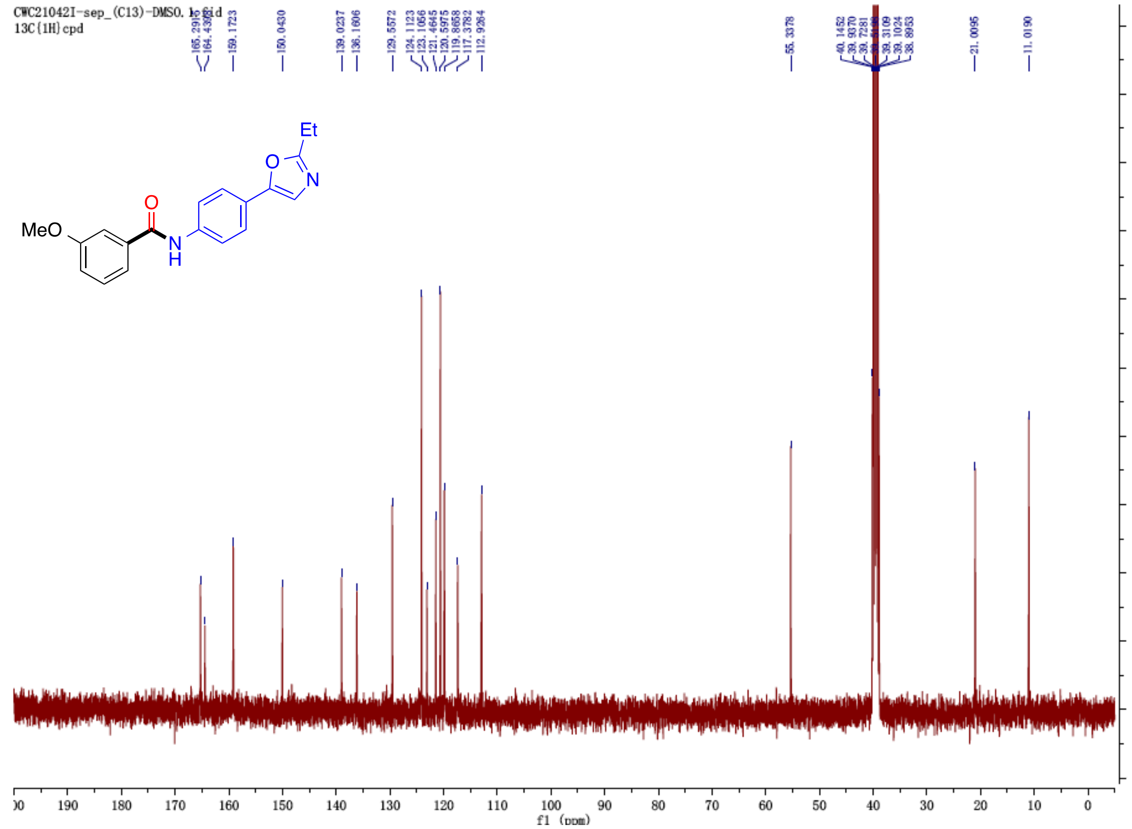

**$^{1}$H and $^{13}$C NMR of *N*-(4-(1,3-dioxolan-2-yl)phenyl)-3-(trifluoromethyl)benzamide (3y).**

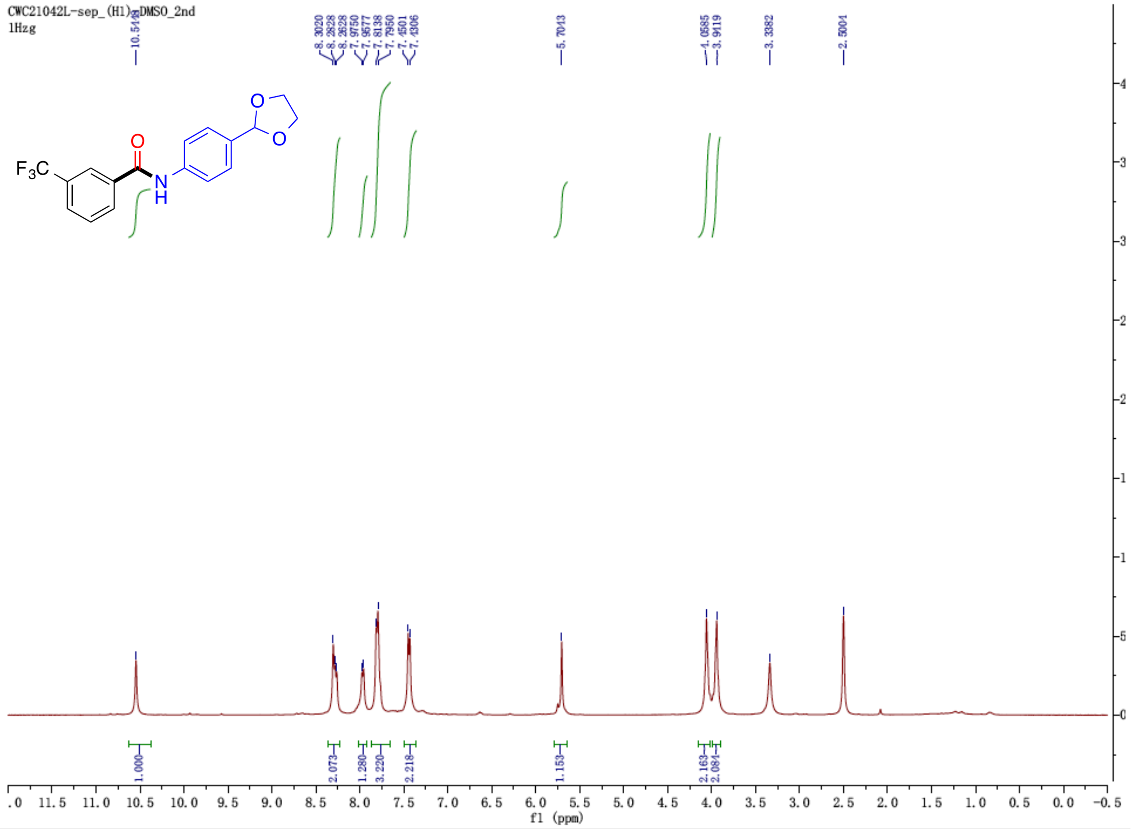


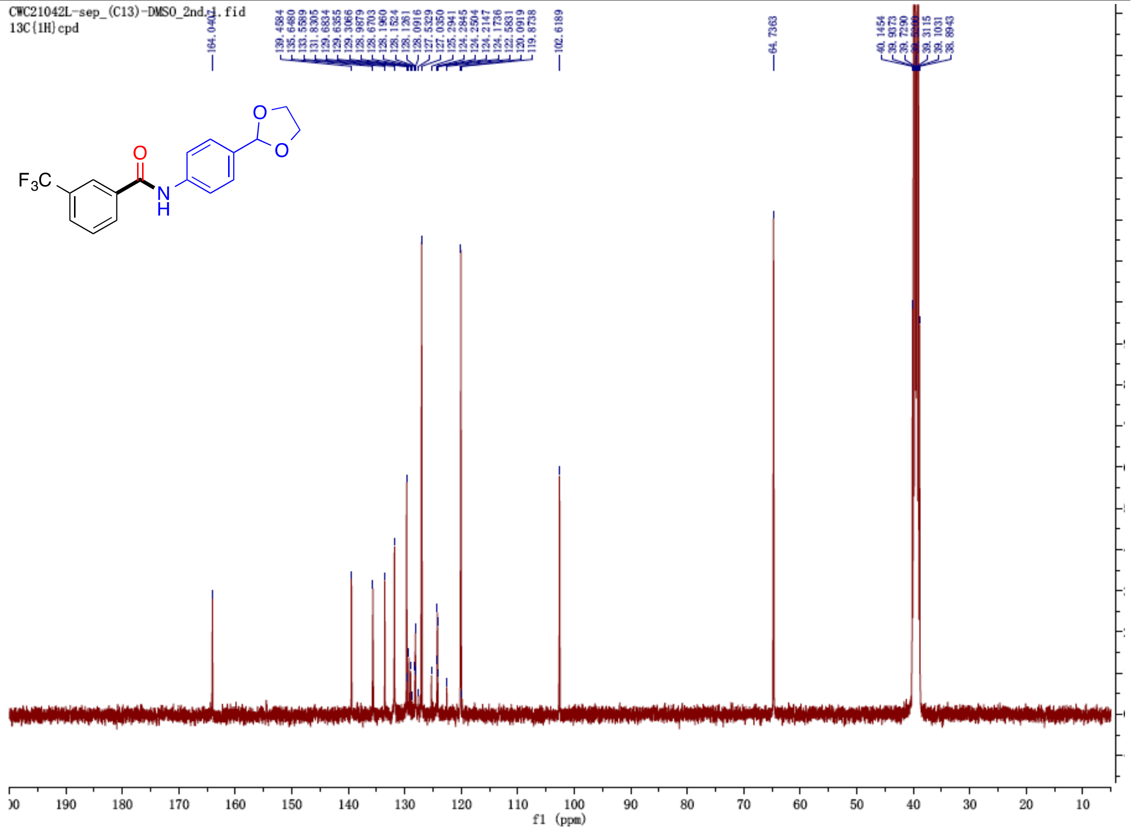

$^1$H and $^{13}$C NMR of *N*-(4-(2-methyl-1,3-dioxolan-2-yl)phenyl)-3-(trifluoromethyl)benzamide (3z).

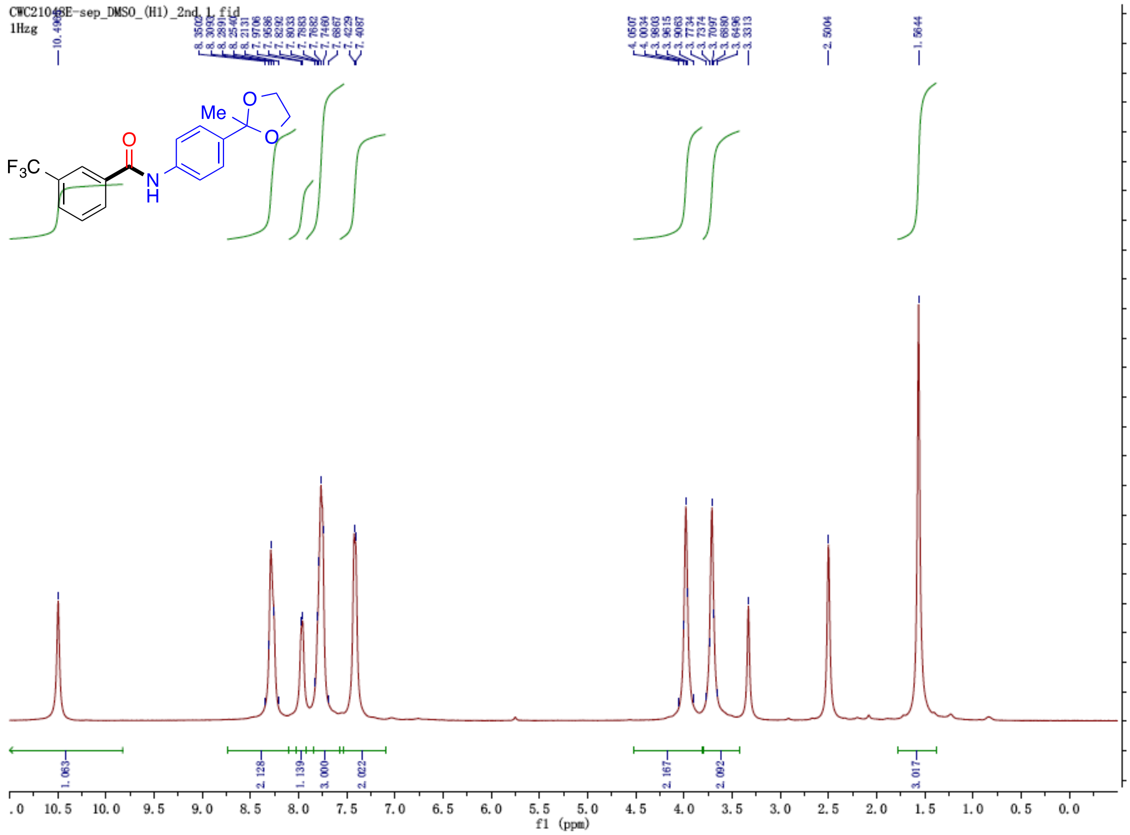

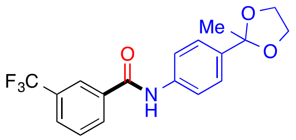

$^{1}$H and $^{13}$C NMR of *N*-(4-(2-((*tert*-butyldimethylsilyl)oxy)ethyl)phenyl)-3-methylbenzamide (3aa).

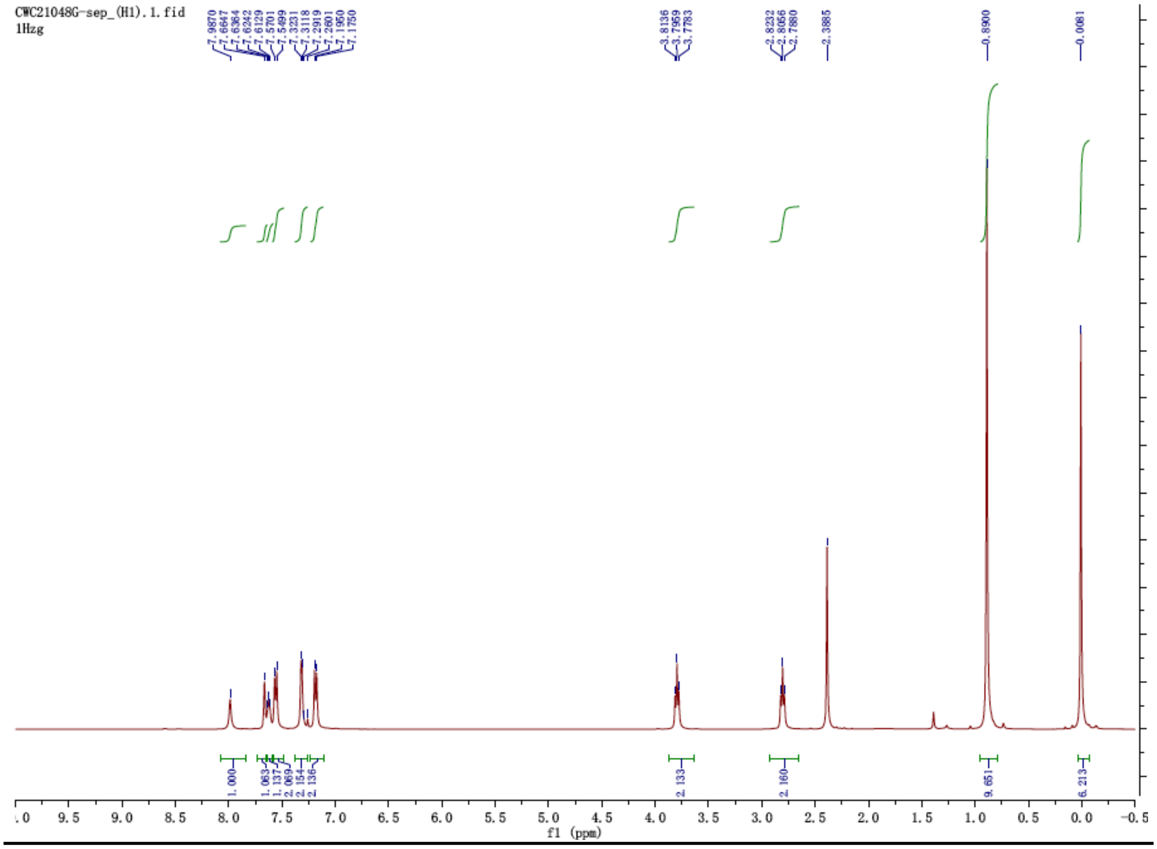

$^{1}$H and $^{13}$C NMR of *N*-(4-(dimethylamino)phenyl)-3-methylbenzamide (3bb).

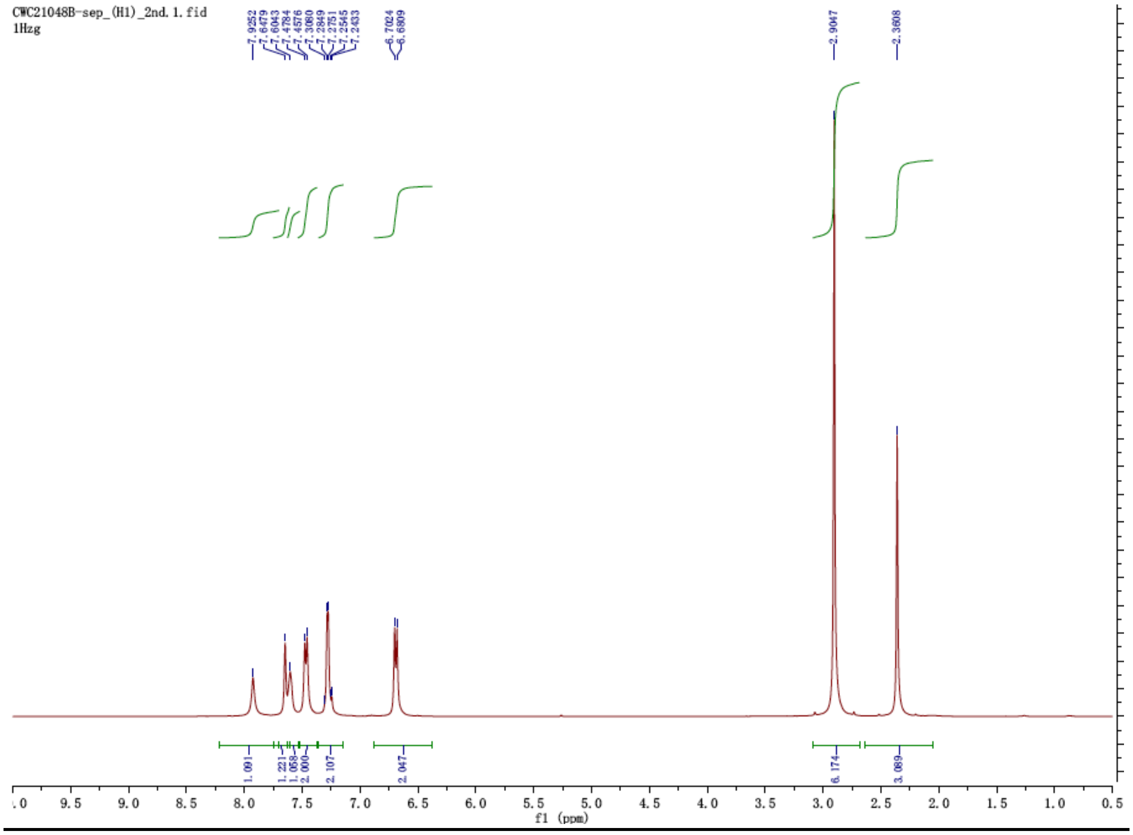

$^{1}H$ and $^{13}C$ NMR of 3-methyl-*N*-(2-phenylbenzo[*d*]oxazol-5-yl)benzamide (3cc).

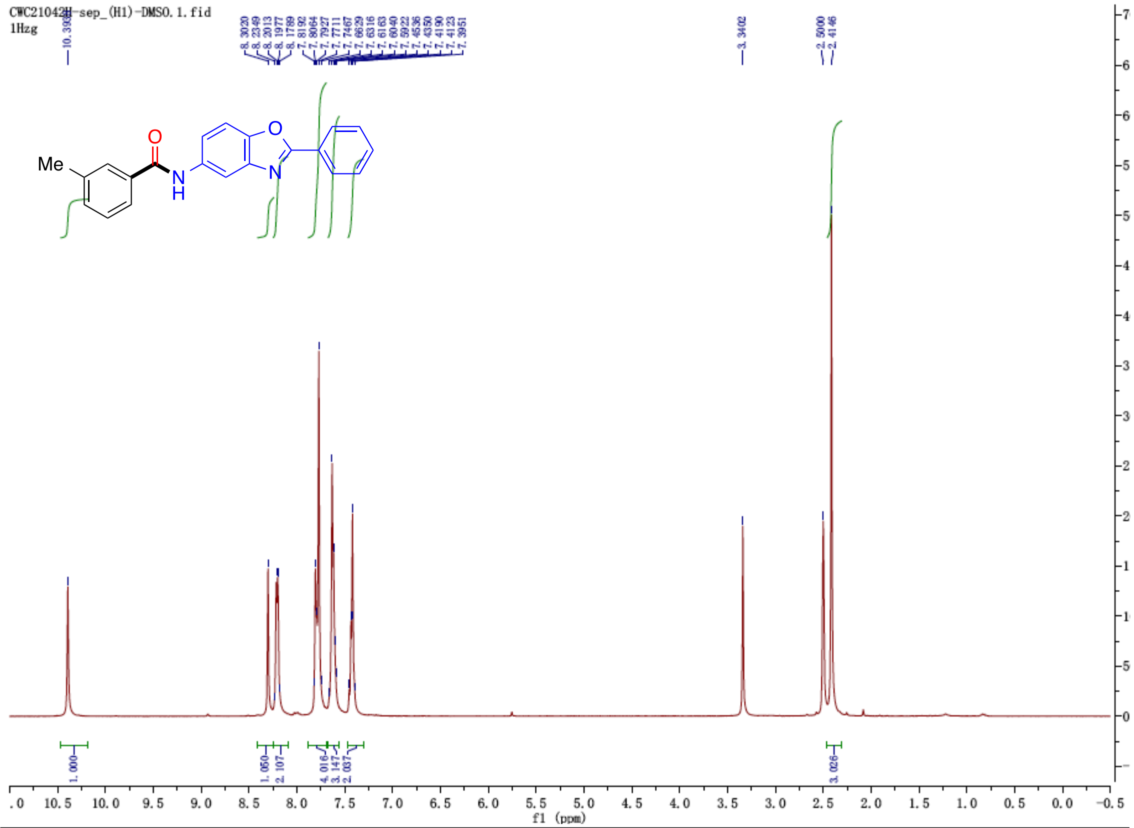

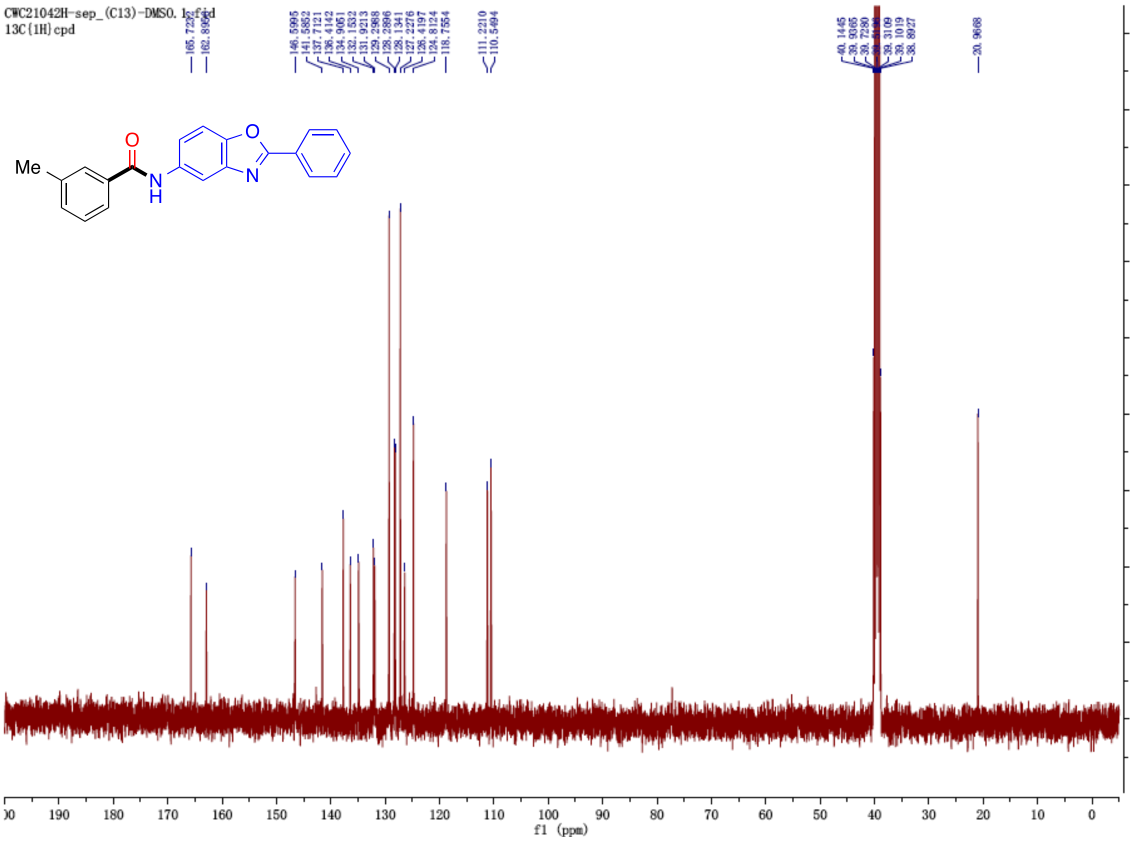

$^{1}$H and $^{13}$C NMR of *N*-(4-(*tert*-butyl)phenyl)picolinamide (3dd).

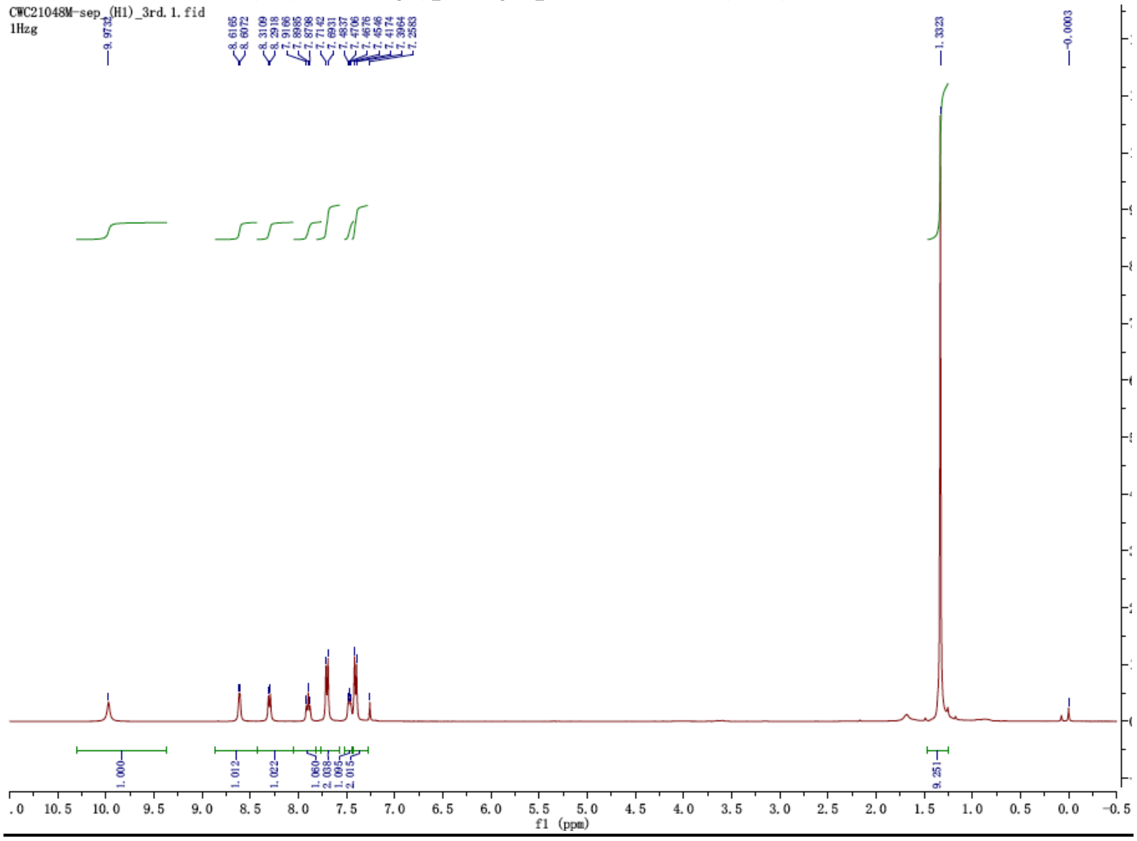

**$^{1}H$ and $^{13}C$ NMR of *N*-(4-(*tert*-butyl)phenyl)-1-methyl-1*H*-pyrazole-4-carboxamide (3ee).**

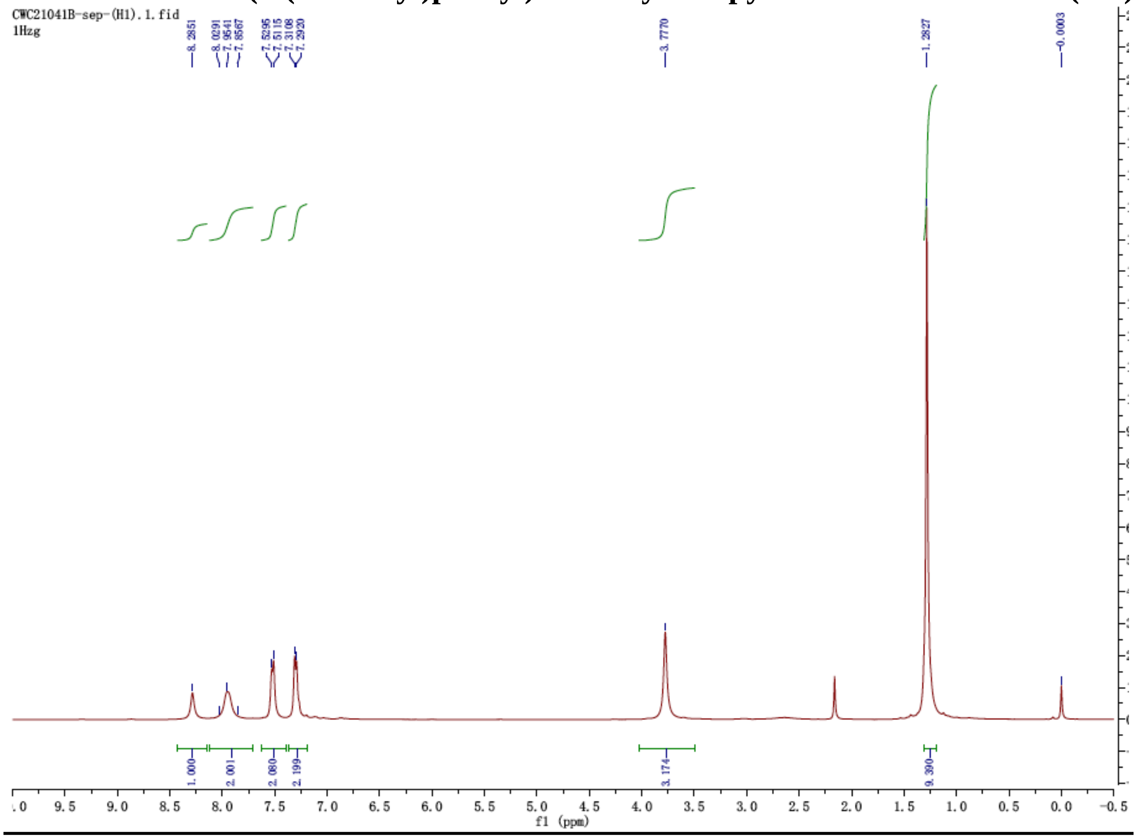

**$^{1}$H and $^{13}$C NMR of 4-(*tert*-butyl)-N-phenylbenzamide (3ff)**

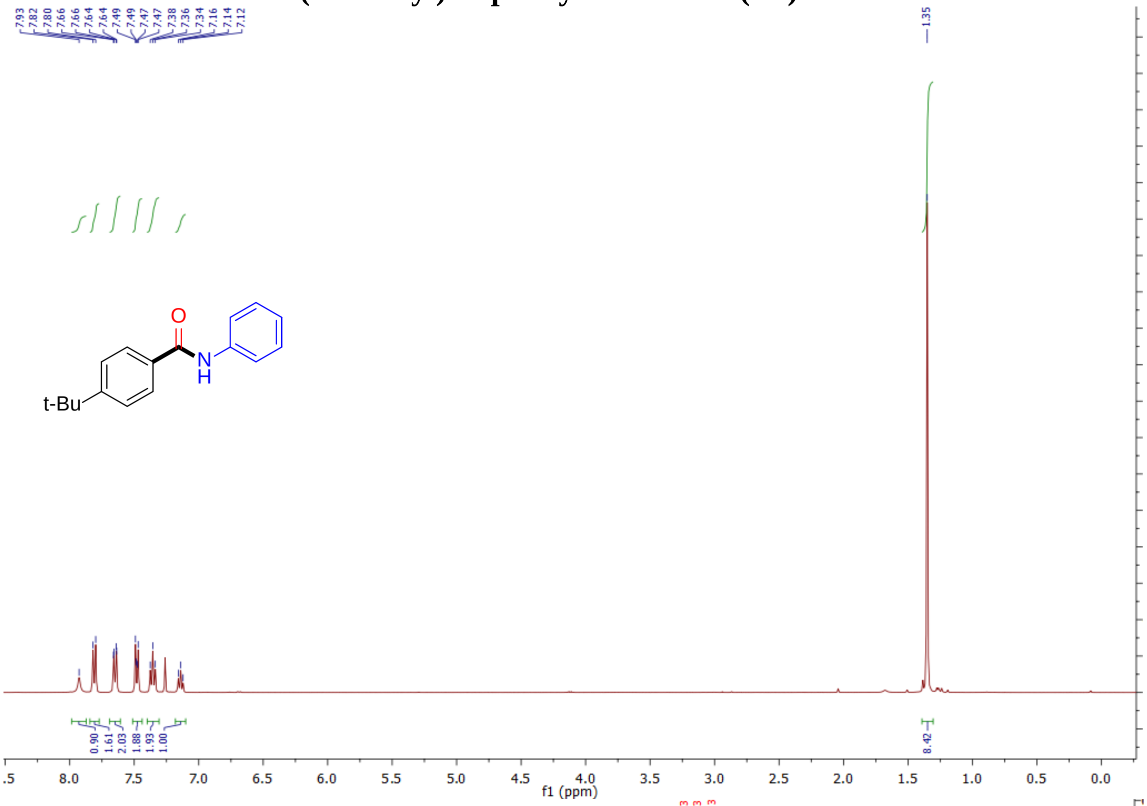

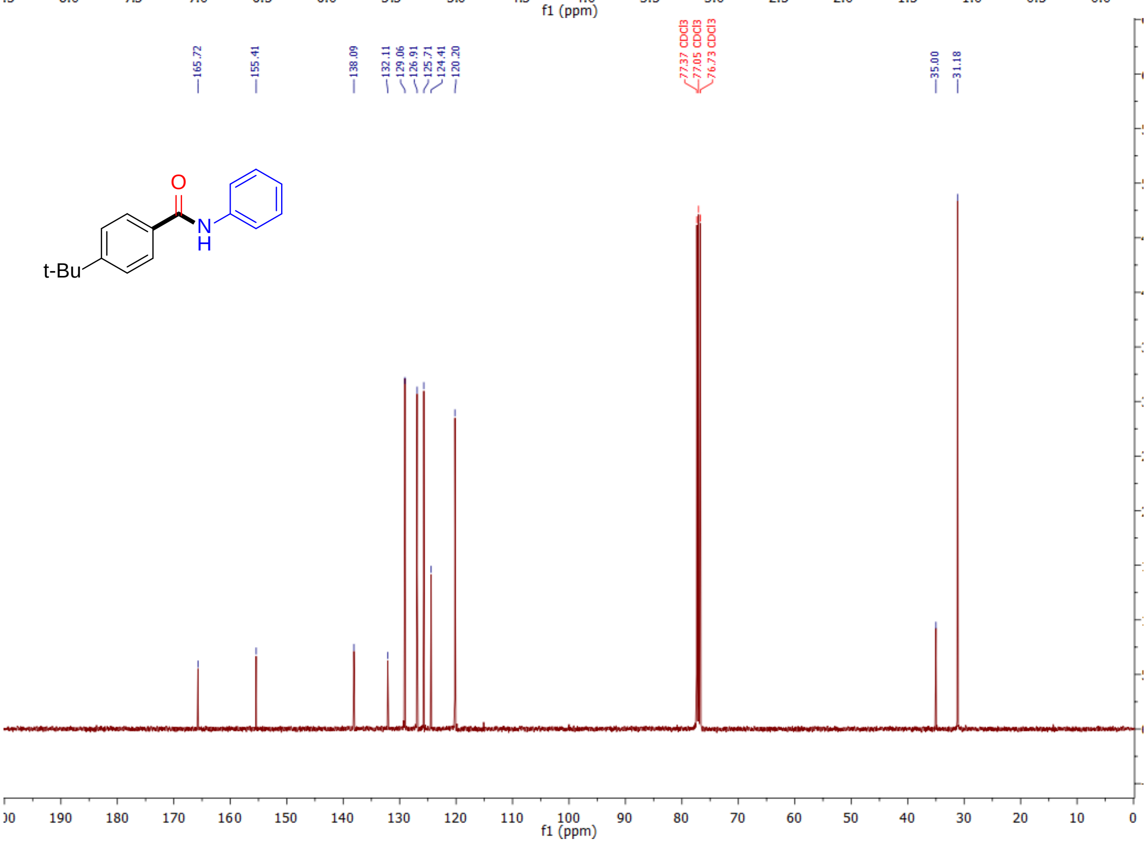

**$^{1}$H and $^{13}$C NMR of 4-(*tert*-butyl)-N-(4-chlorophenyl)benzamide (3gg).**

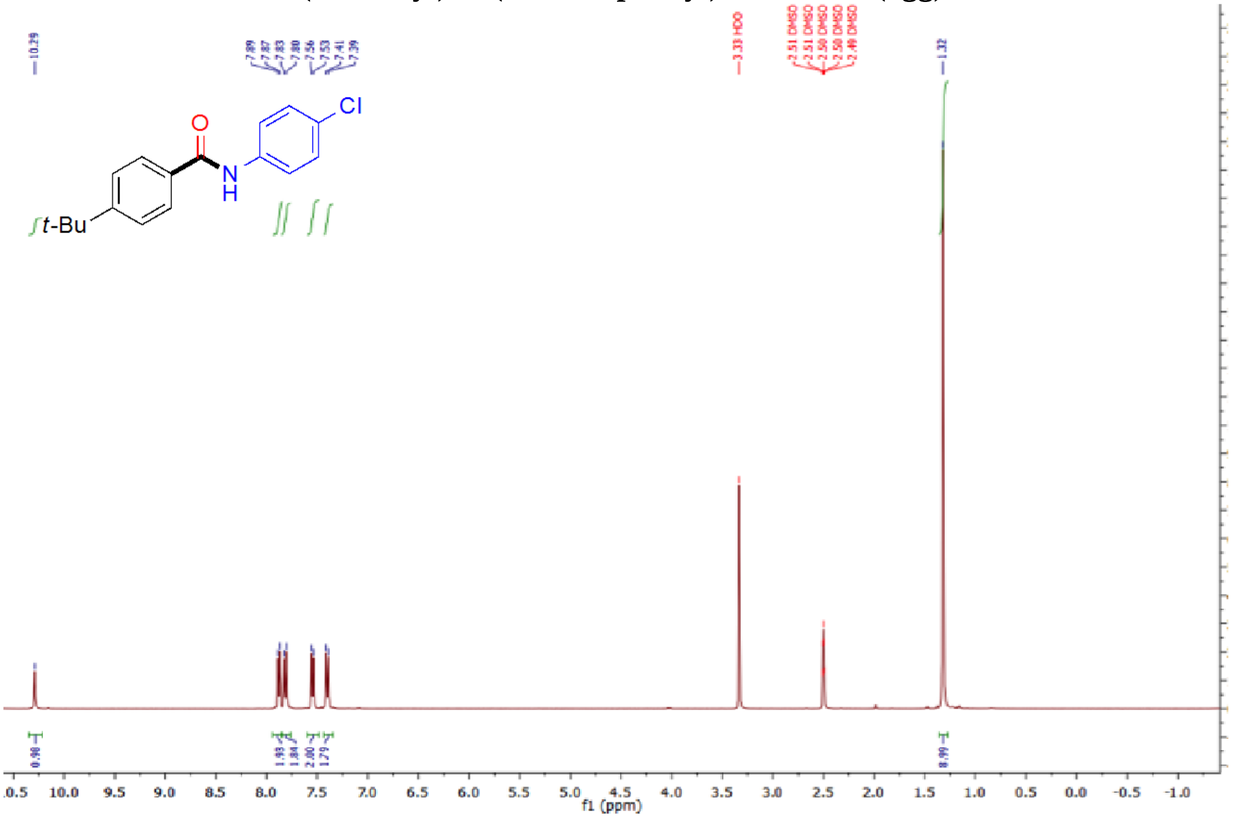

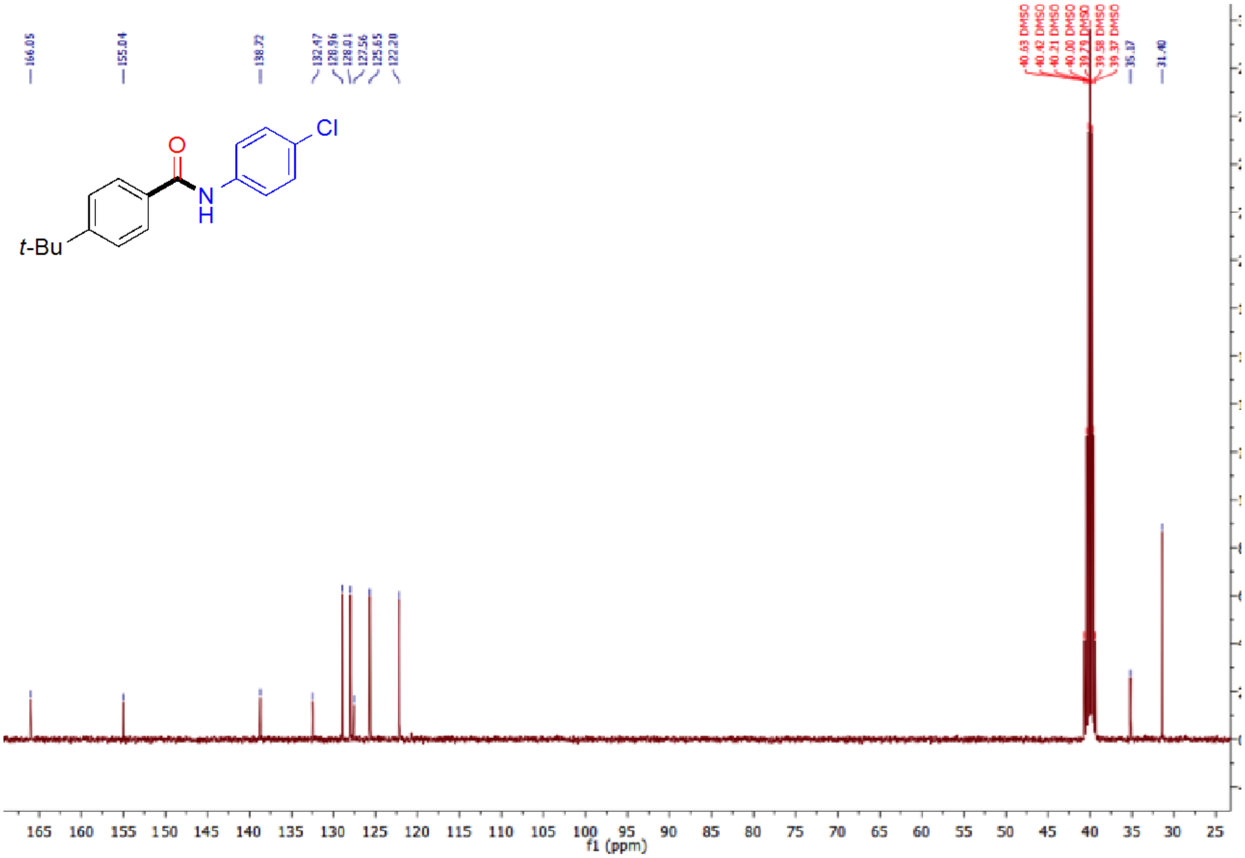

$^{1}H$ and $^{13}C$ NMR of 4-methoxy-*N*-(quinolin-6-yl)benzamide (4a).

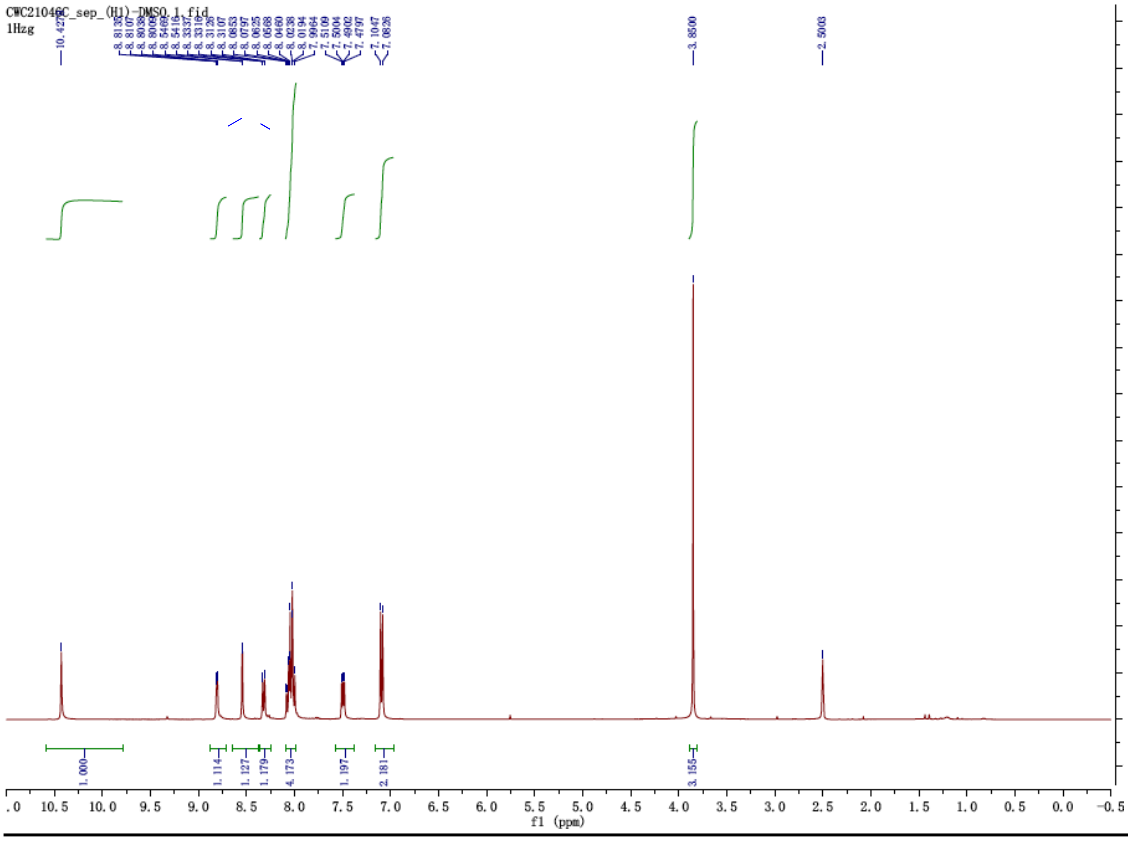

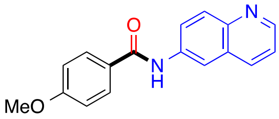

$^{1}$H and $^{13}$C NMR of 4-fluoro-*N*-(4-(phenylamino)phenyl)benzamide (4b).

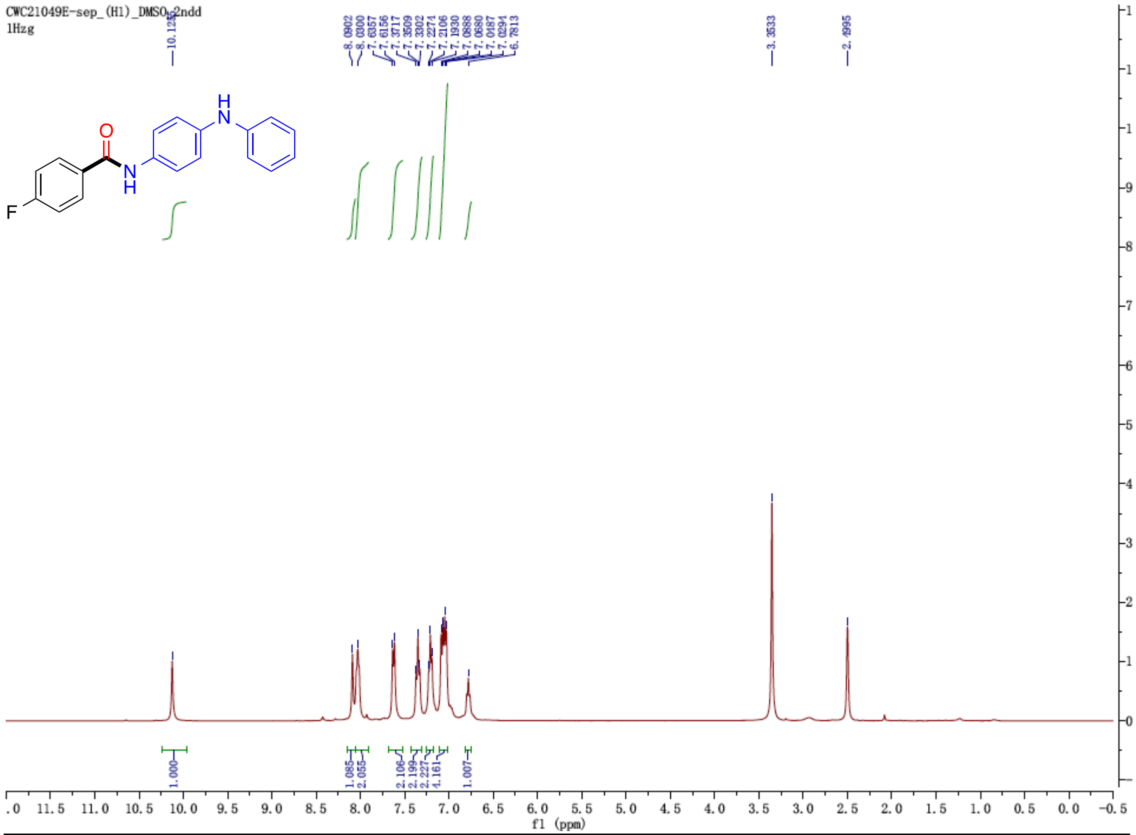


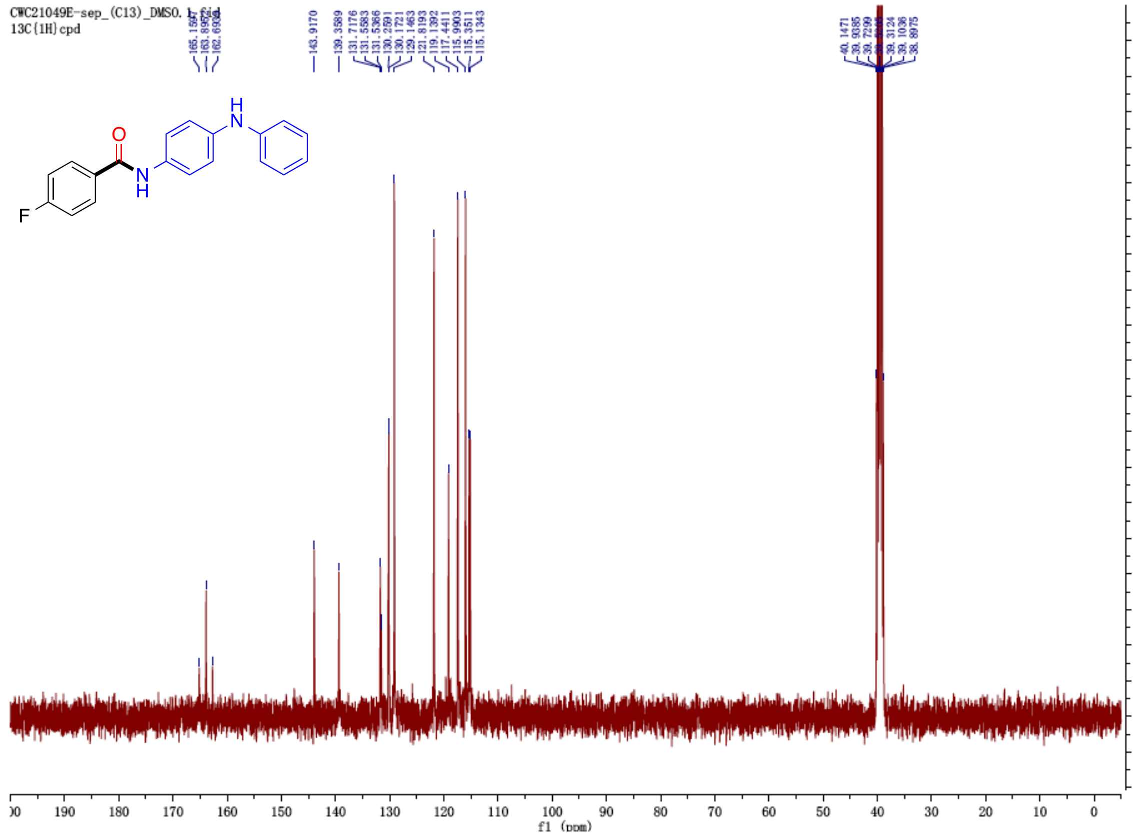

**$^{1}$H and $^{13}$C NMR of 4-benzoyl-*N*-(4-methoxyphenyl)benzamide (4c).**

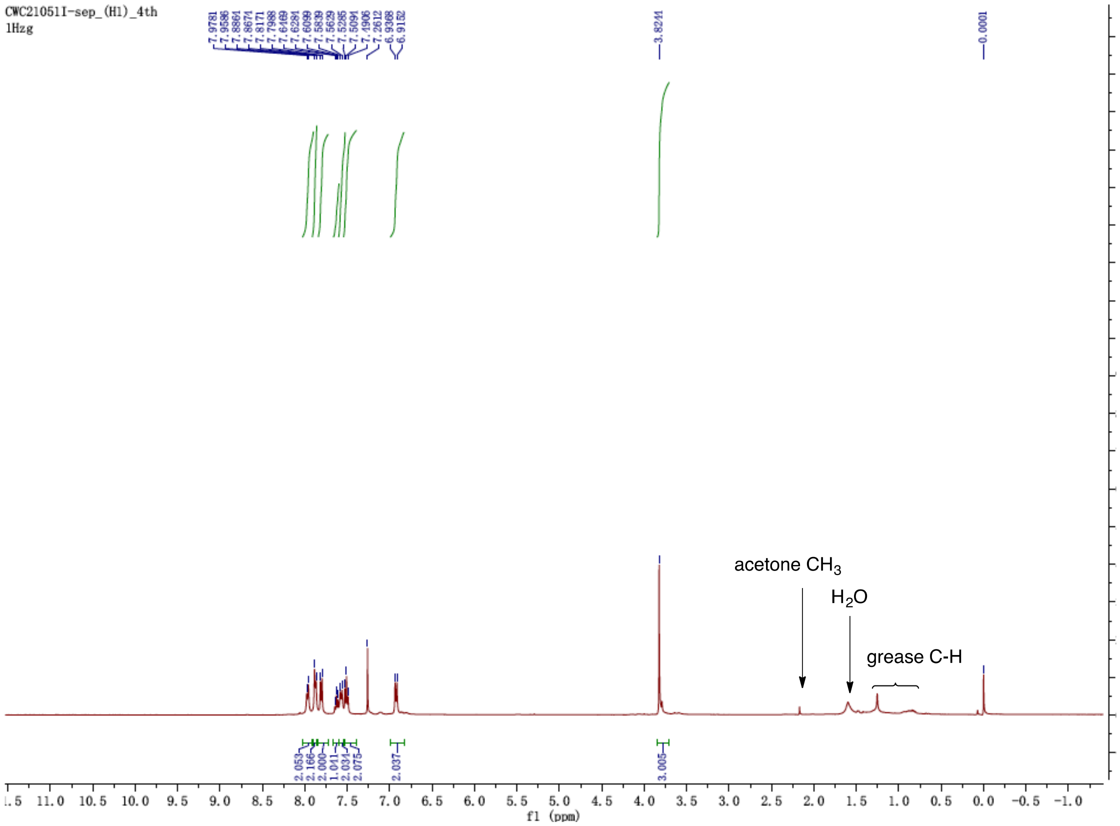


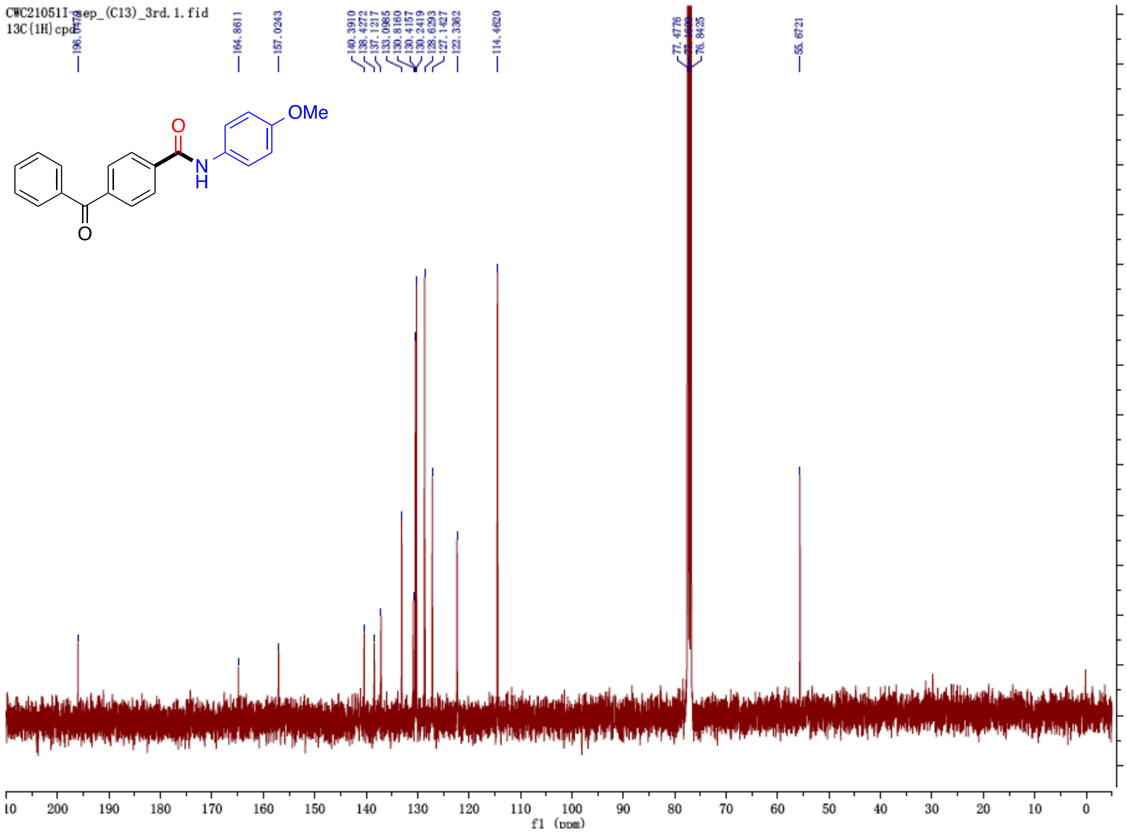

**$^{1}$H and $^{13}$C NMR of *N*$^{1}$,*N*$^{1}$-diethyl-*N*$^{4}$-(*p*-tolyl)terephthalamide (4d).**

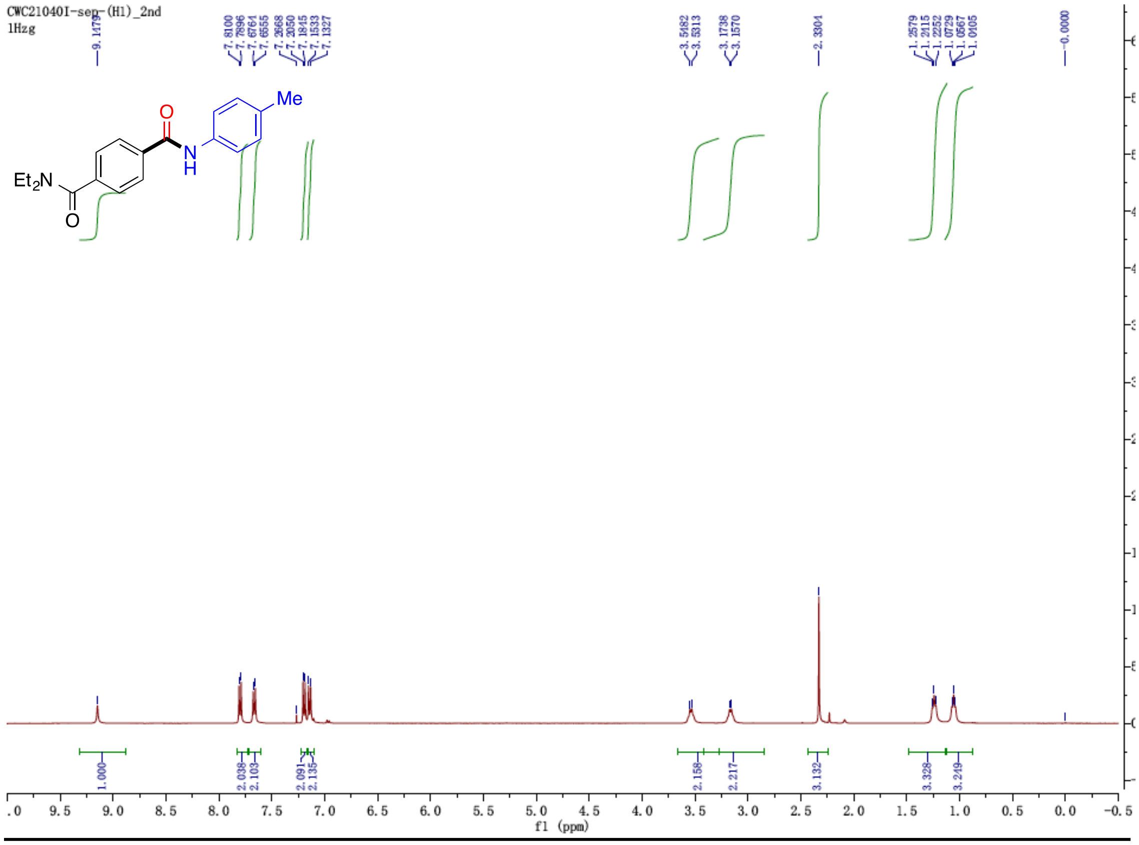

**$^{1}$H and $^{13}$C NMR of *N*-(3-methoxy-4-methylphenyl)-4-(trifluoromethyl)benzamide (4e).**

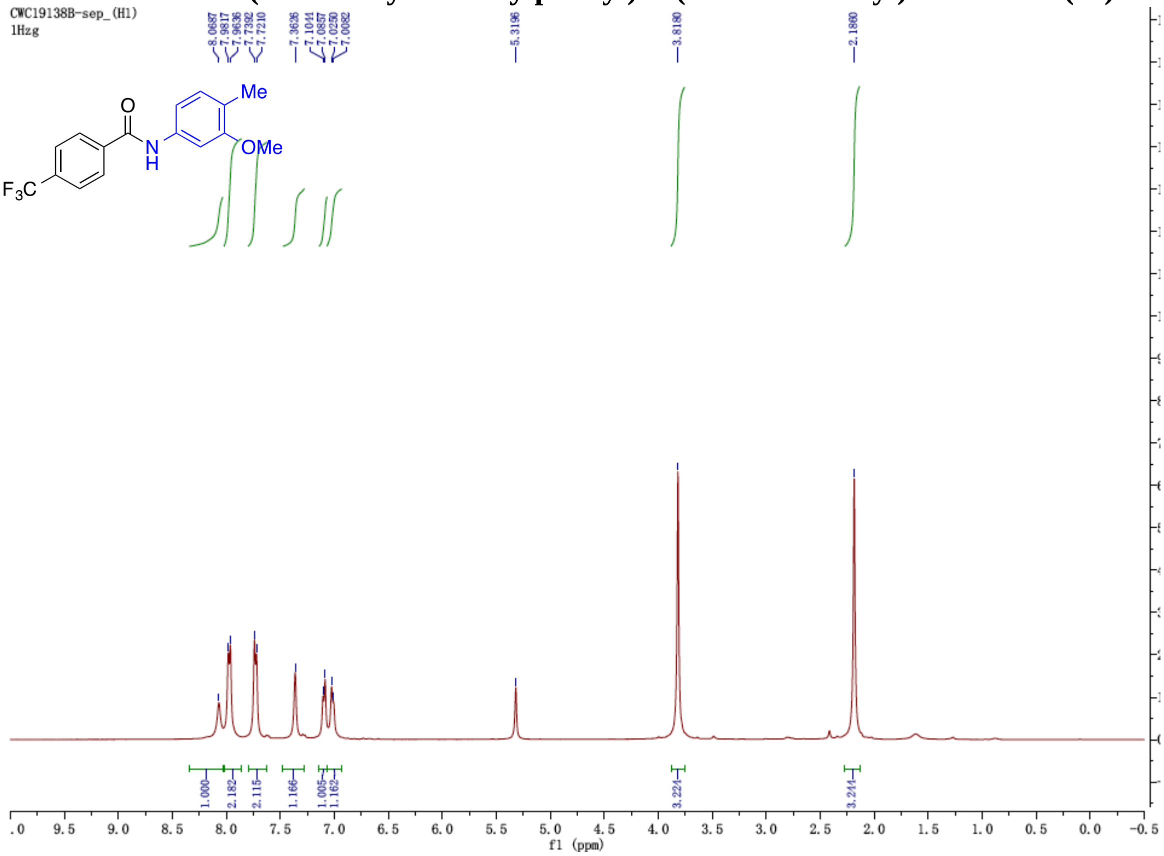

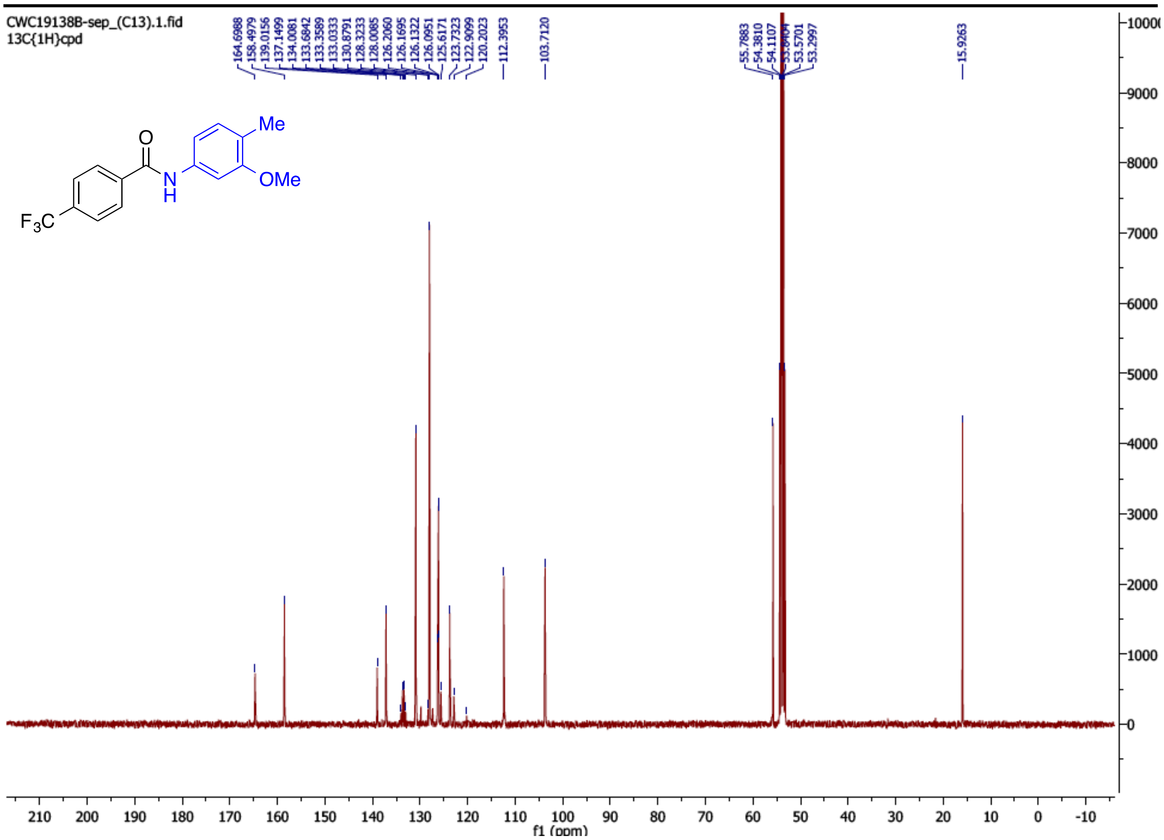

$^1$H and $^{13}$C NMR of *N*-(benzo[*d*][1,3]dioxol-5-yl)-3-(dimethylamino)benzamide (4f).

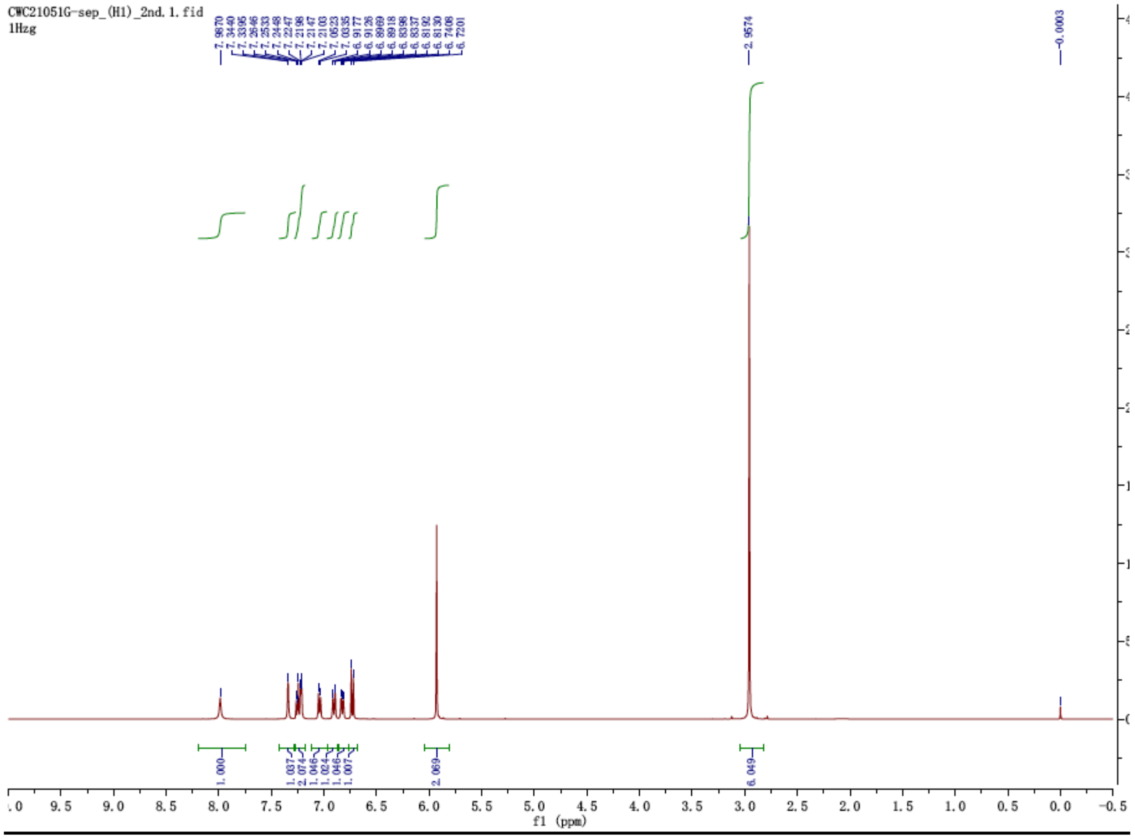

**$^{1}H$ and $^{13}C$ NMR of *N*-(4-methoxyphenyl)-3-propionylbenzamide (4g).**

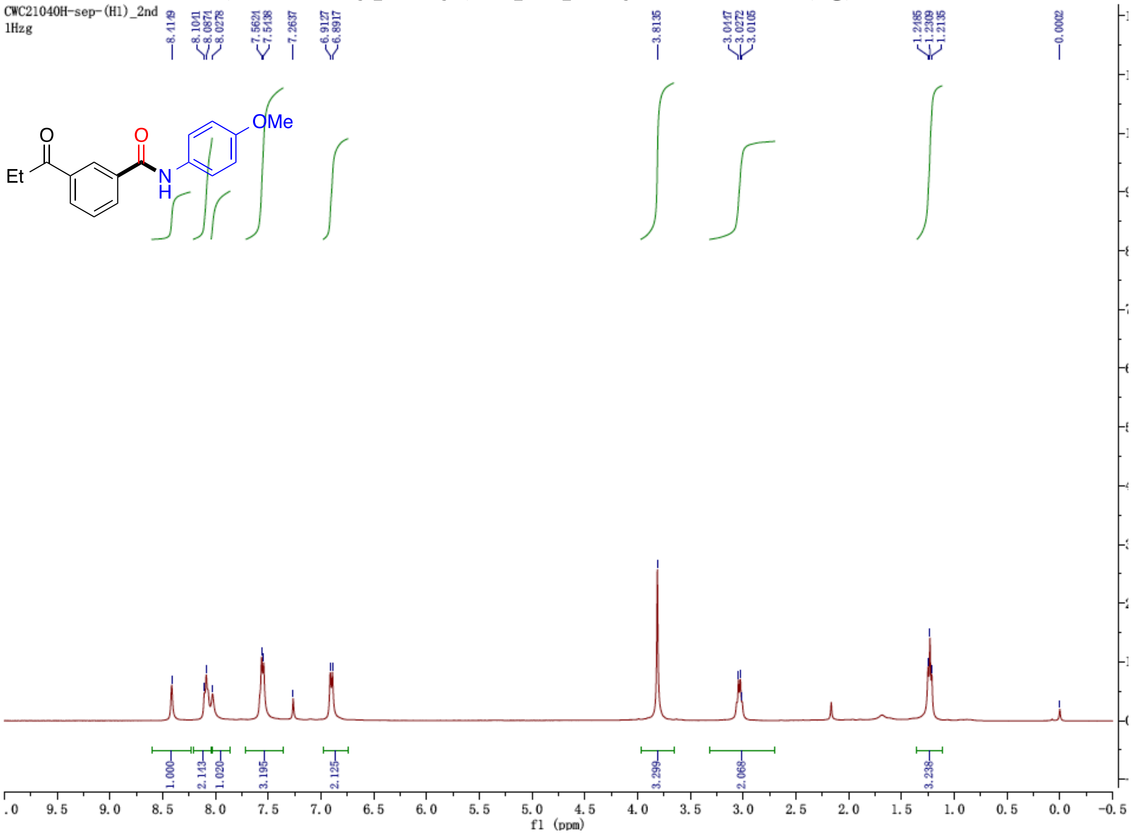

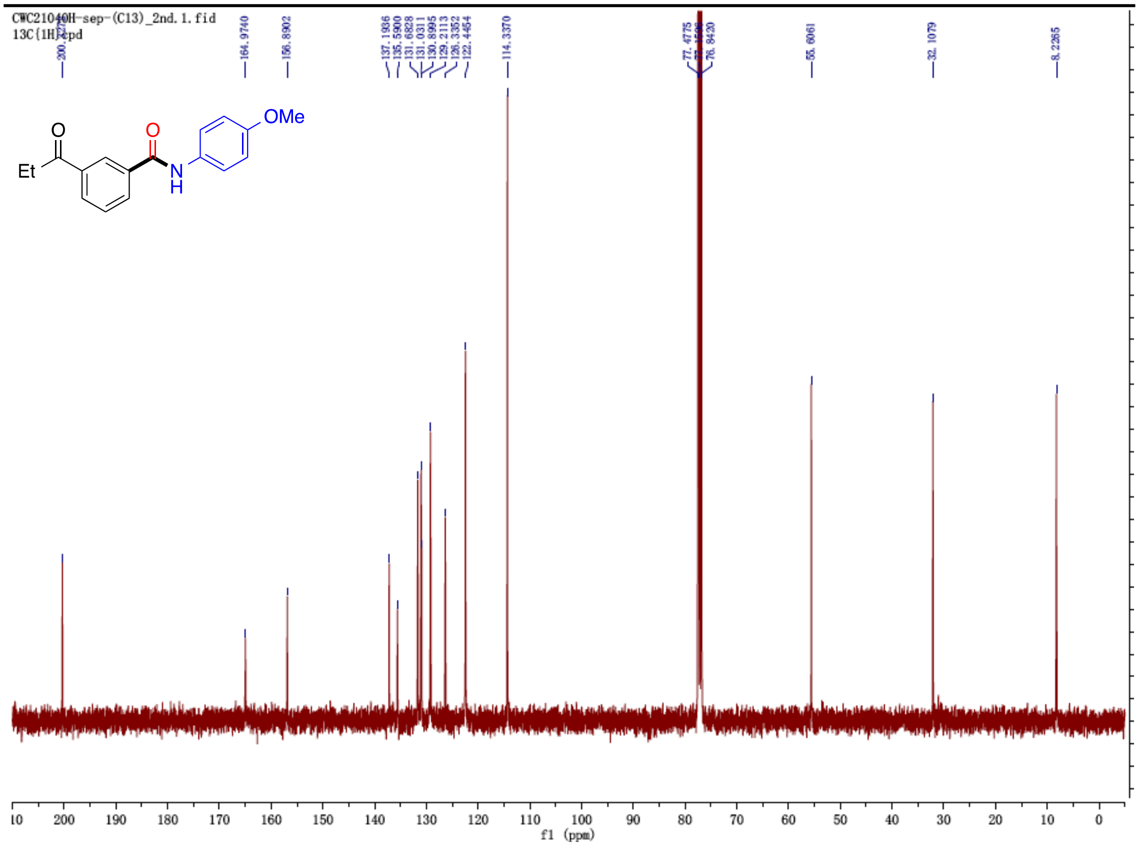

$^{1}H$ and $^{13}C$ NMR of *N*-(9*H*-fluoren-2-yl)-3-(trifluoromethyl)benzamide (4h).

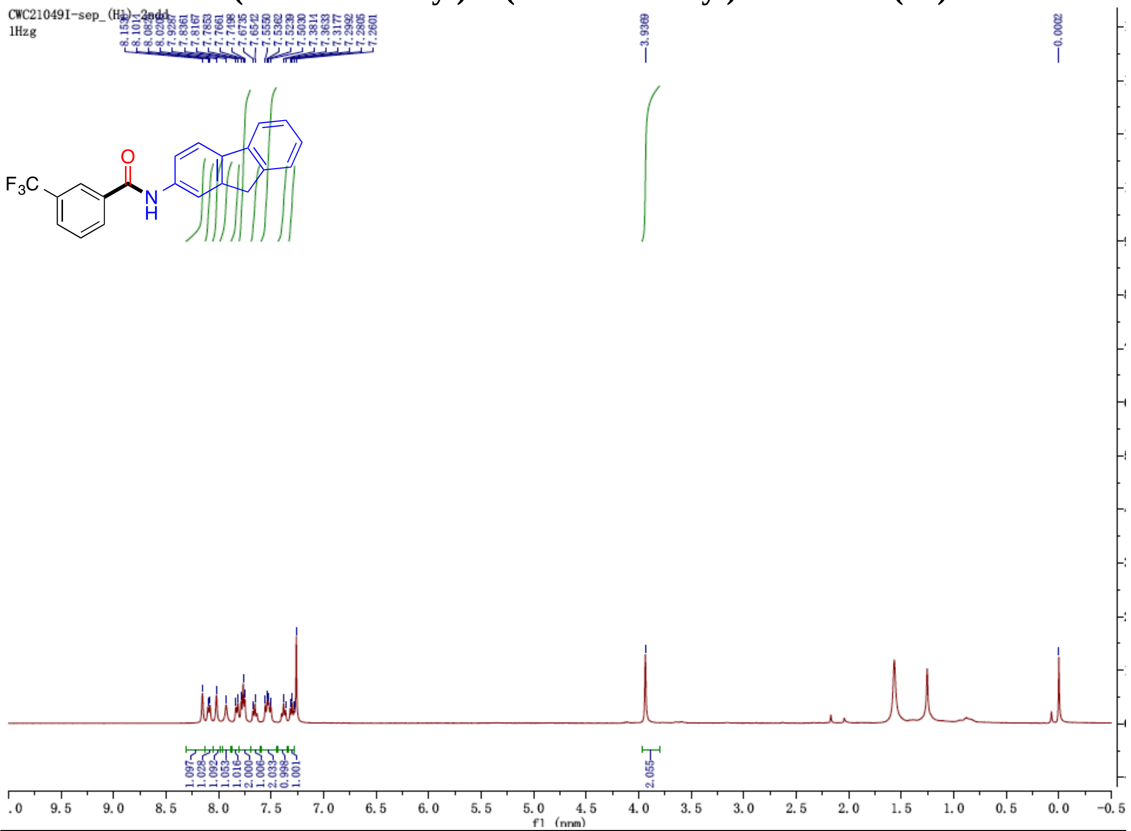

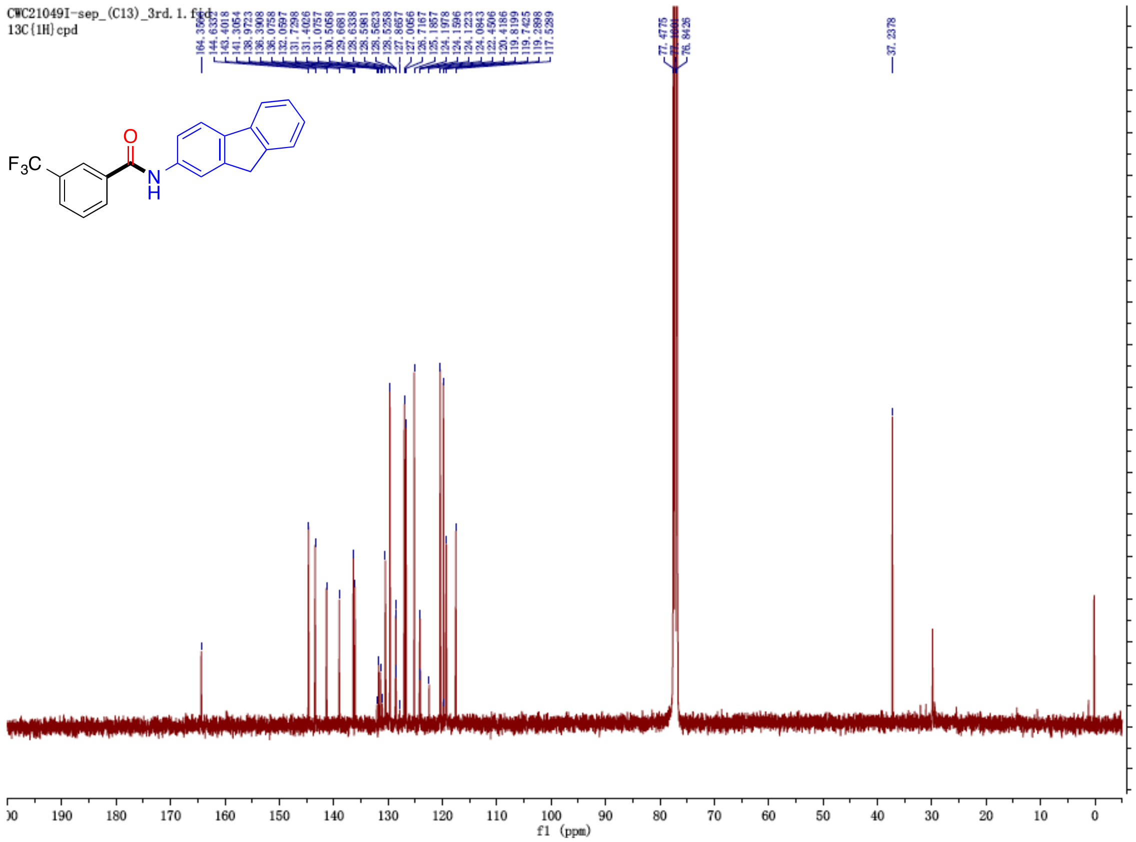

$^{1}H$ and $^{13}C$ NMR of *N*-(3-(trifluoromethyl)phenyl)-2-naphthamide (4i).

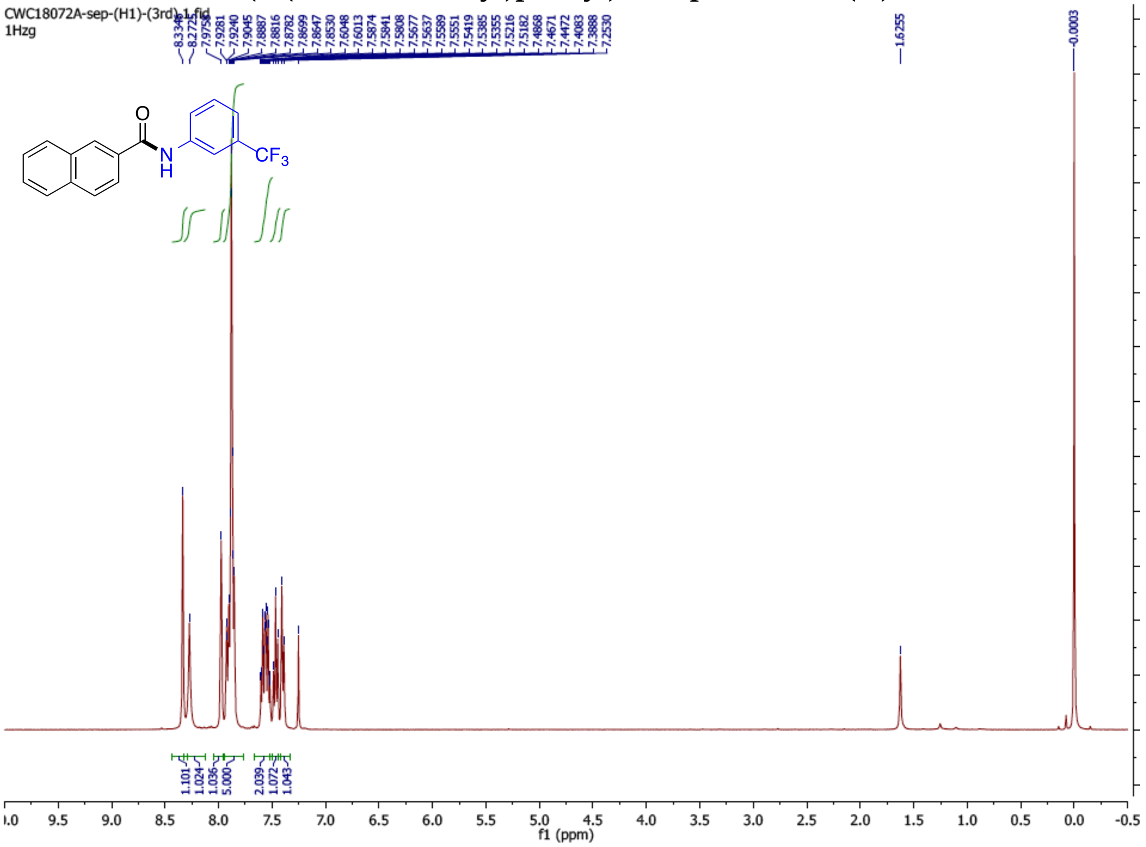


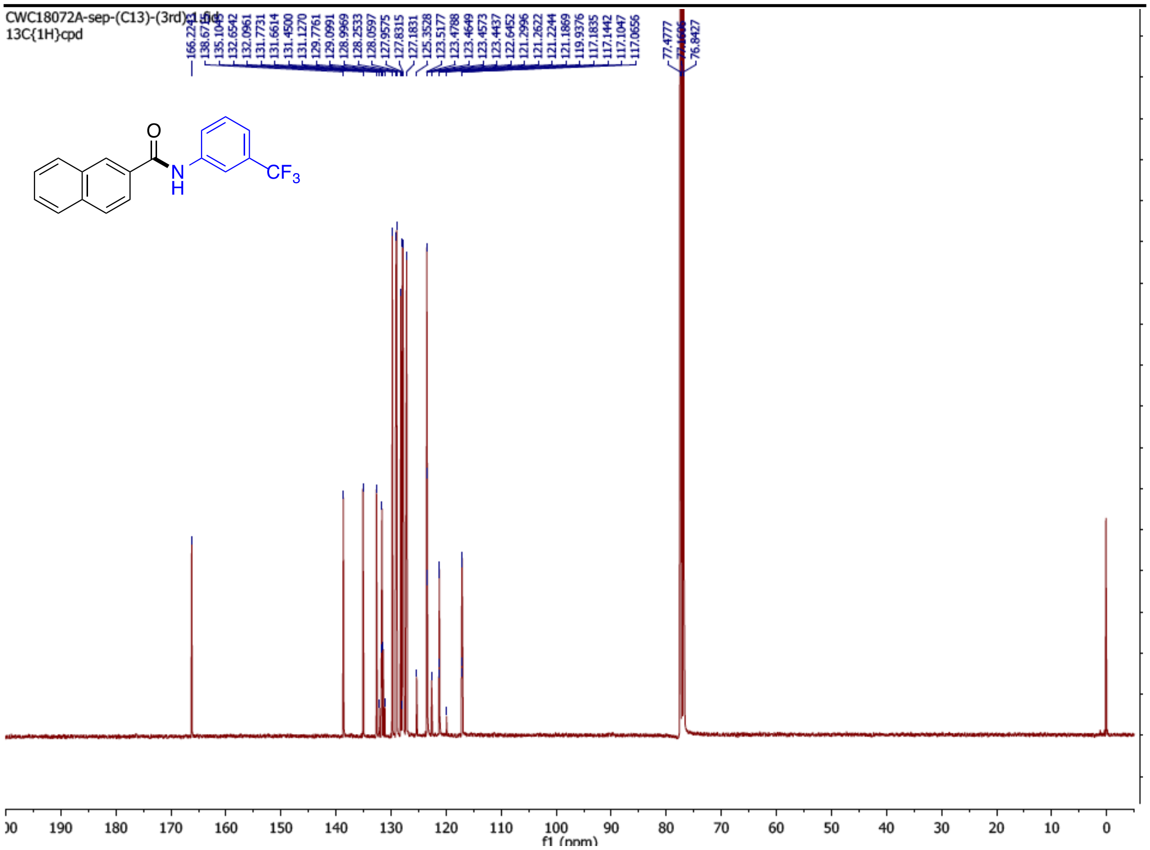

**$^{1}$H and $^{13}$C NMR of *N*-(4-(*tert*-butyl)phenyl)quinoline-3-carboxamide (4j).**

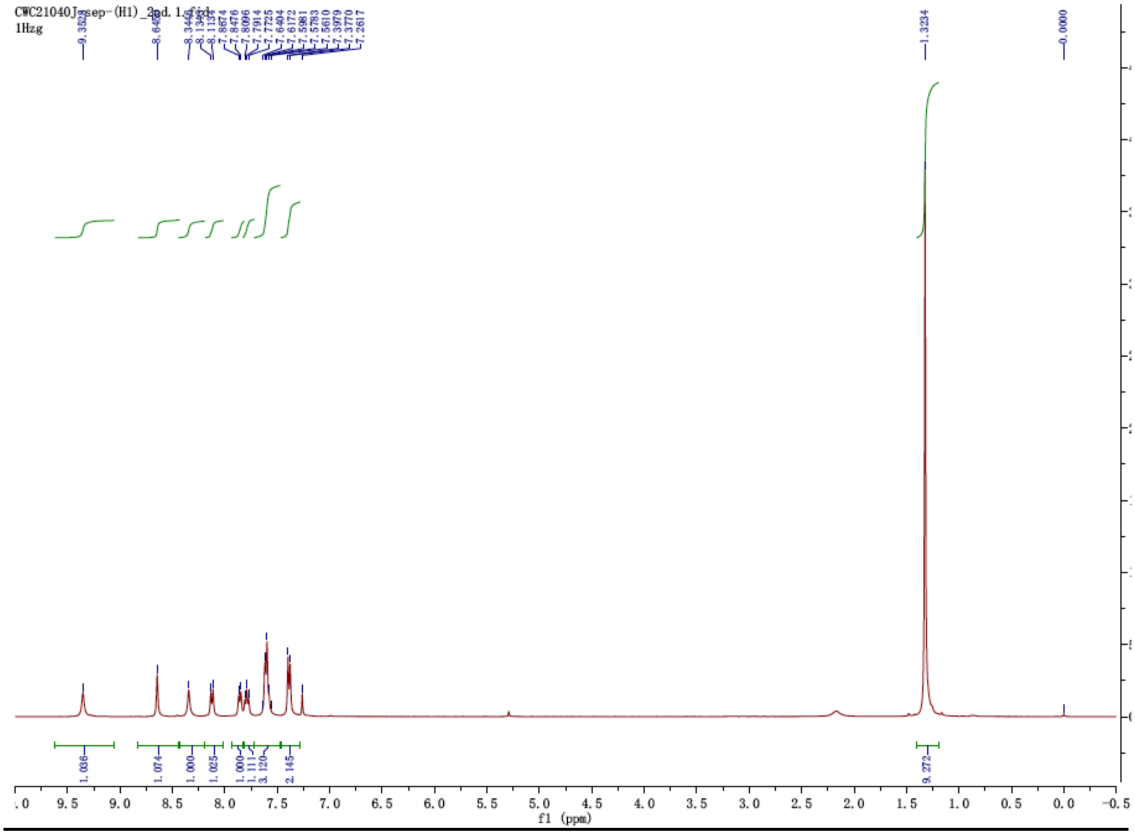

**$^{1}H$ and $^{13}C$ NMR of 1-benzyl-*N*-(4-(*tert*-butyl)phenyl)-1*H*-indole-5-carboxamide (4k).**

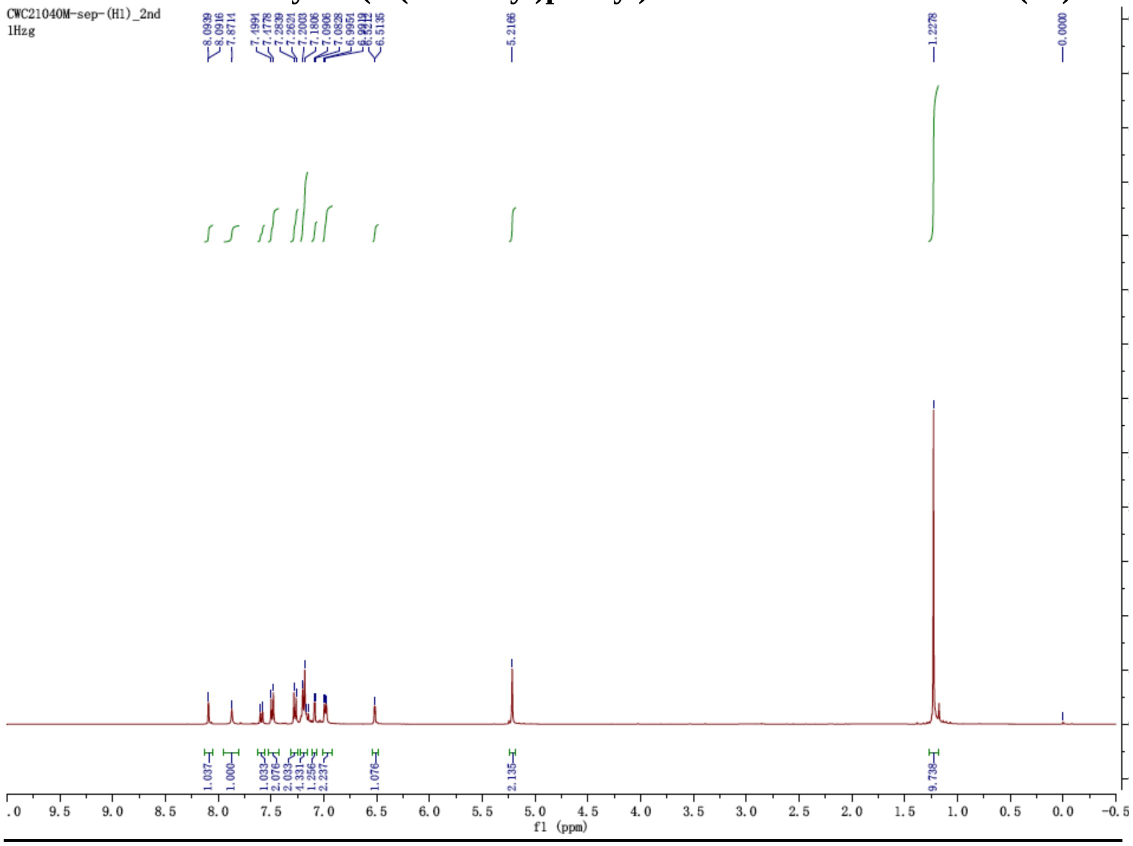

$^{1}$H and $^{13}$C NMR of *N*-(4-methoxyphenyl)-2-methyl-2*H*-indazole-5-carboxamide (4l).

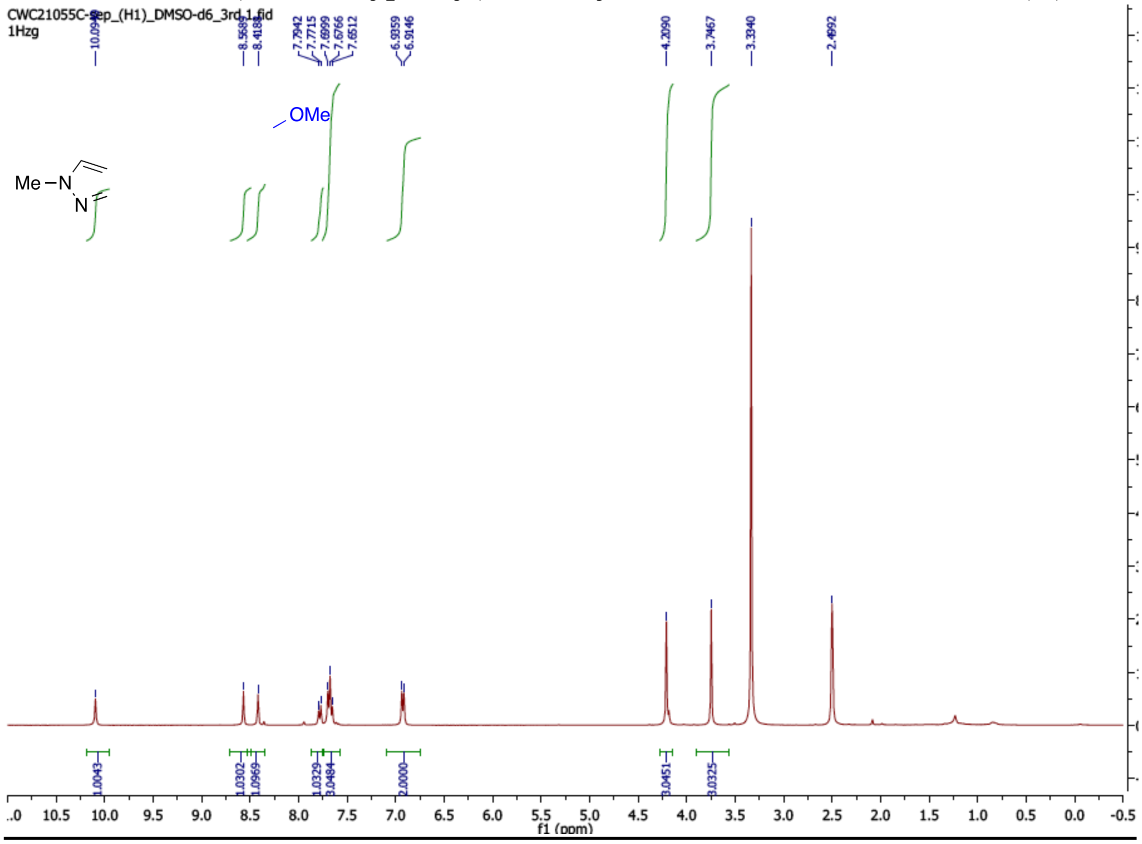

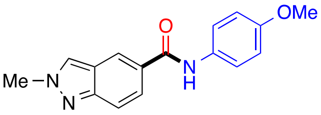

**$^{1}H$ and $^{13}C$ NMR of *N*-(4-(*p*-tolyloxy)phenyl)benzofuran-5-carboxamide (4m).**

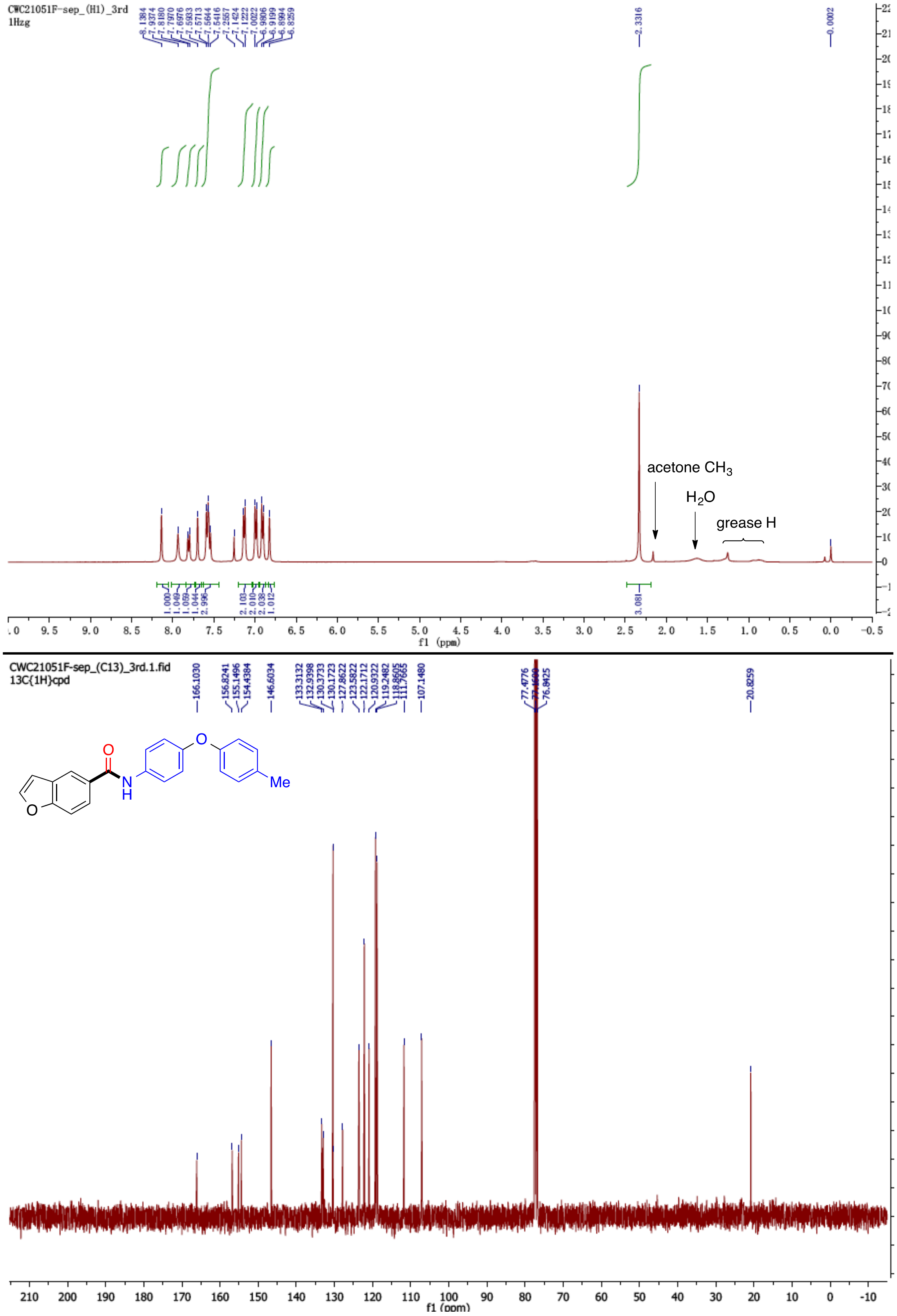

# $^{1}$H and $^{13}$C NMR of *N*-(4-(*tert*-butyl)phenyl)-9-ethyl-9*H*-carbazole-3-carboxamide (4n).

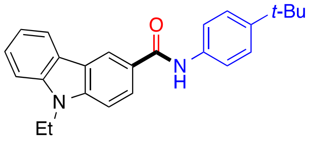

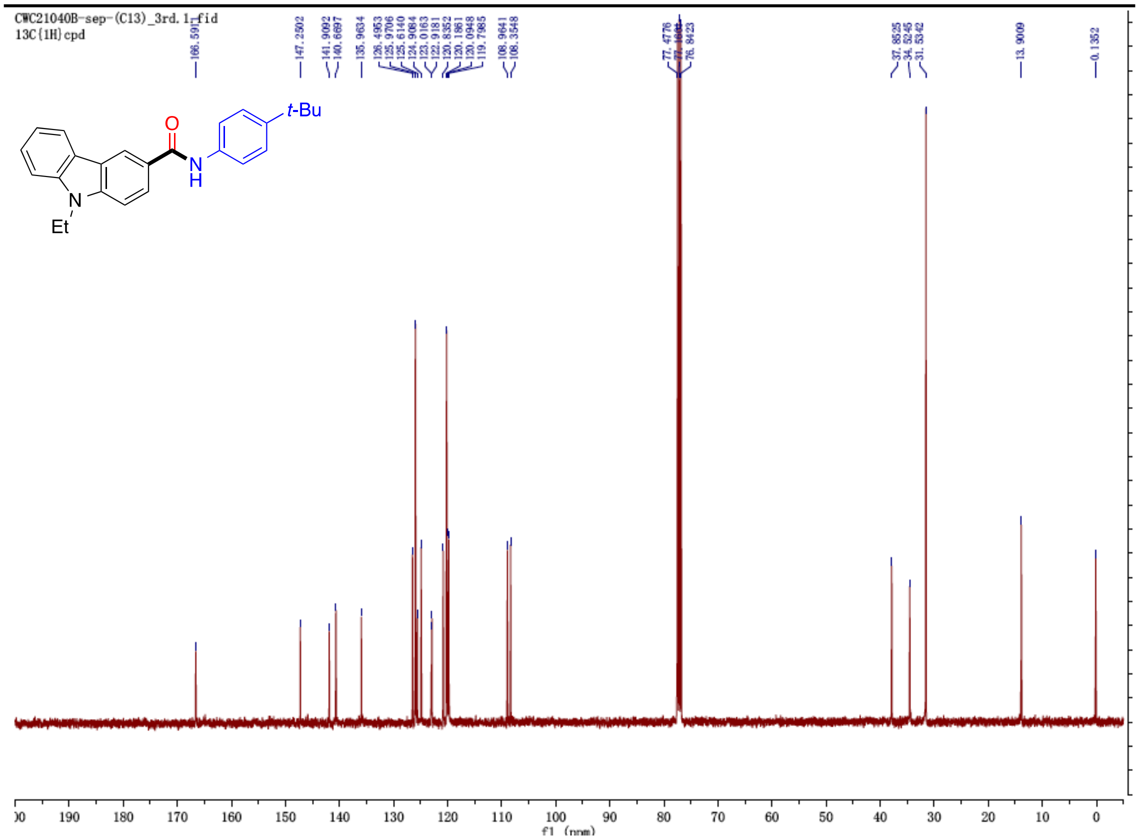

$^{1}$H and $^{13}$C NMR of *N*-([1,1'-biphenyl]-3-yl)dibenzo[*b*,*d*]thiophene-3-carboxamide (4o).

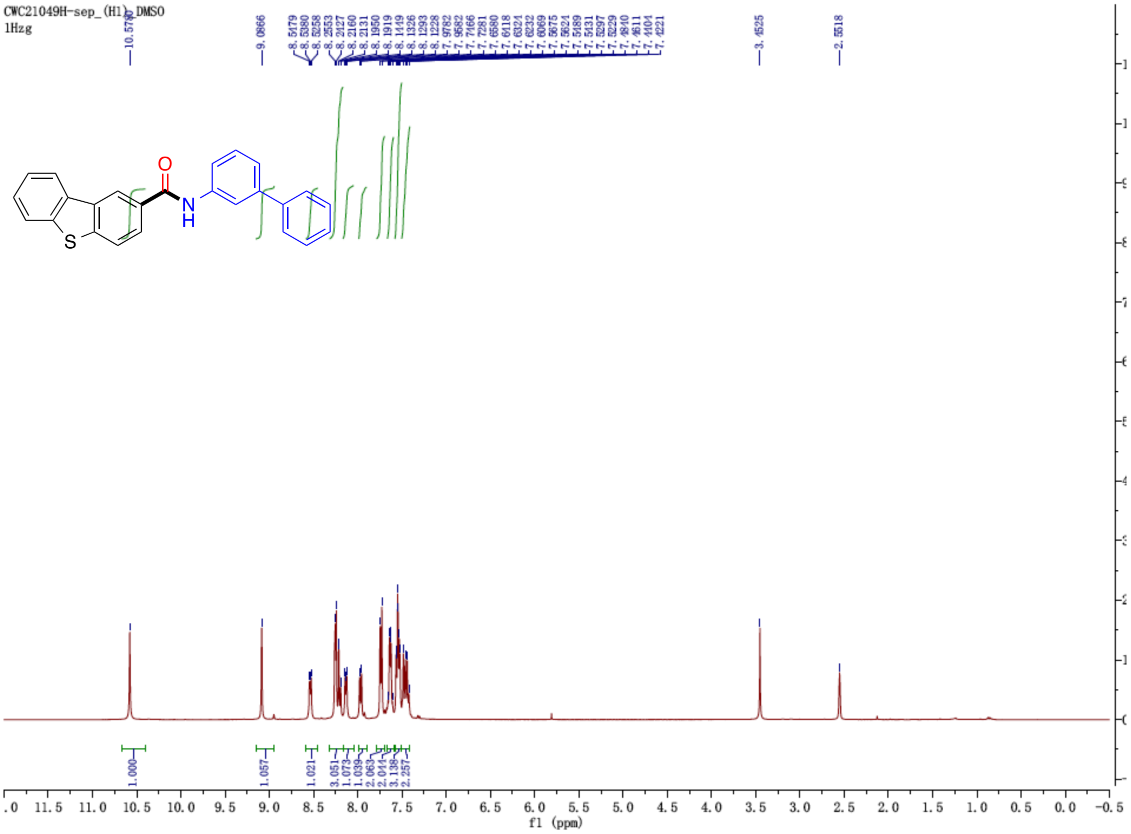

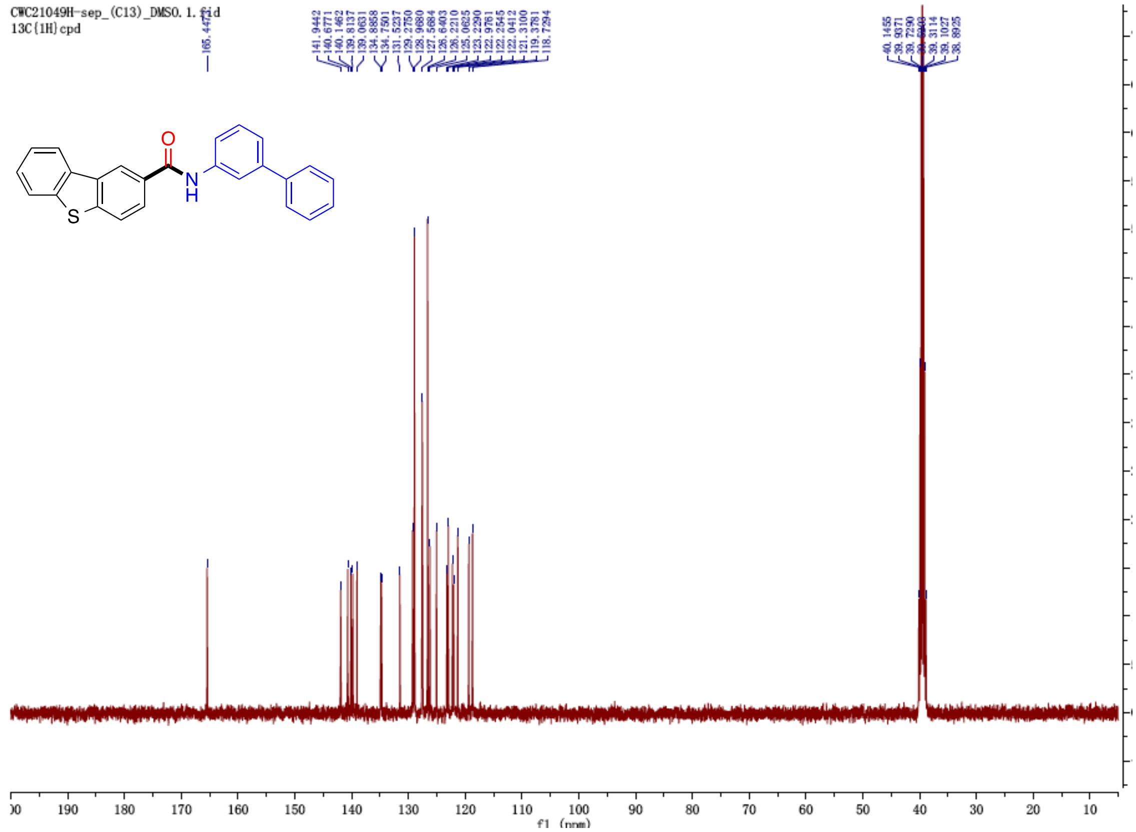

$^{1}H$ and $^{13}C$ NMR of (*E*)-*N*-(2,5-dimethoxyphenyl)-3-(4-fluorophenyl)acrylamide (4p).

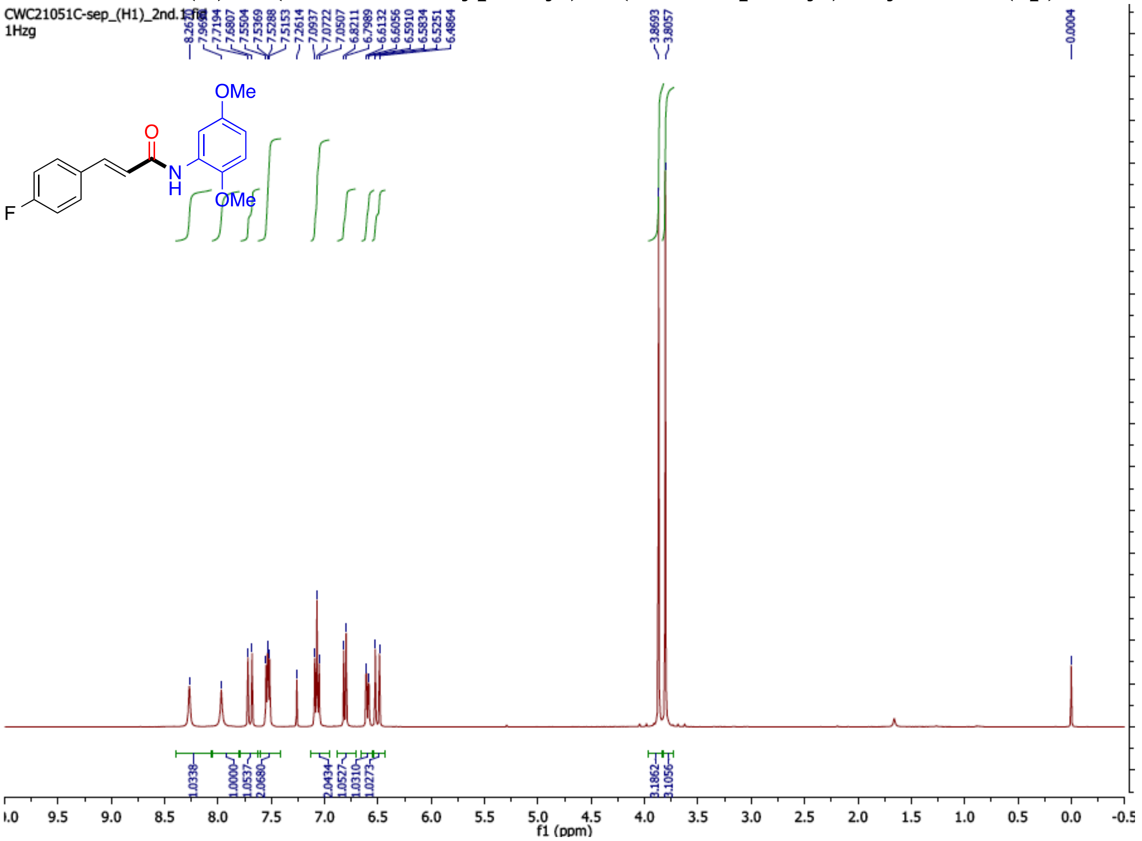

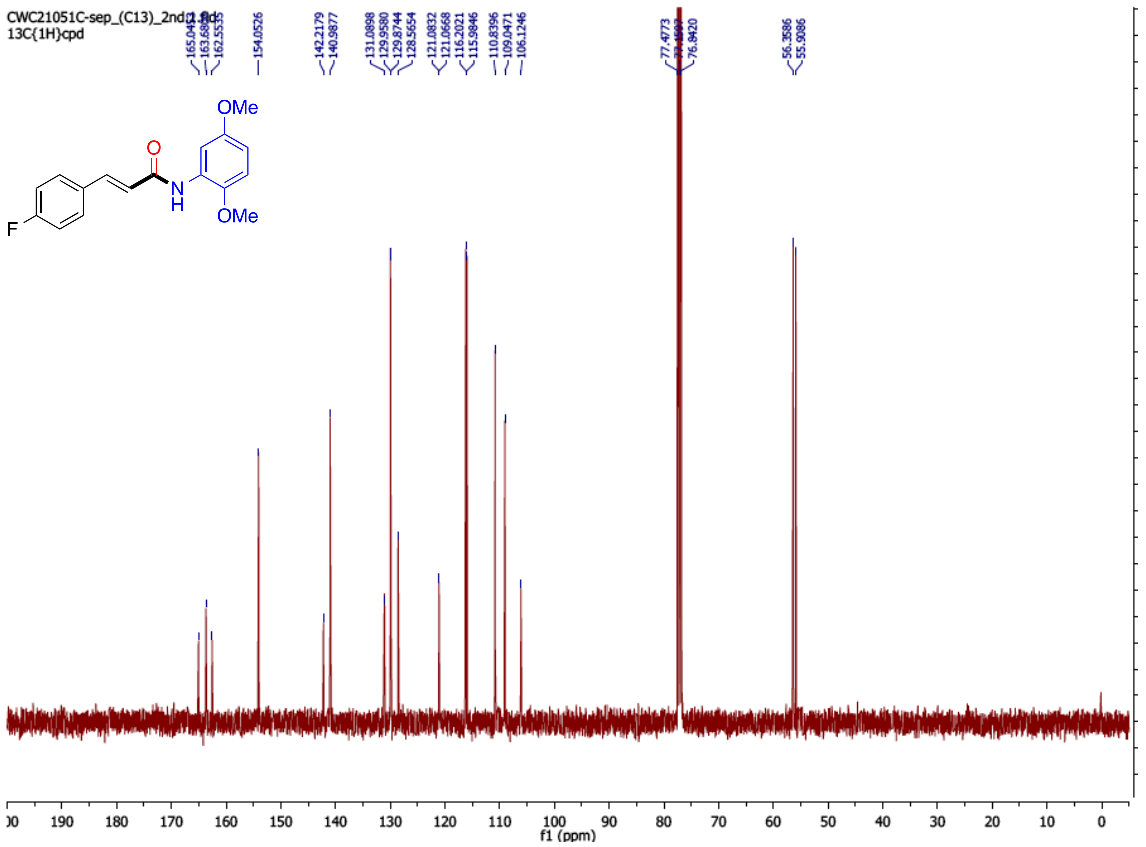

$^{1}$H and $^{13}$C NMR of *N*-(4-chloro-3-methoxyphenyl)picolinamide (5).

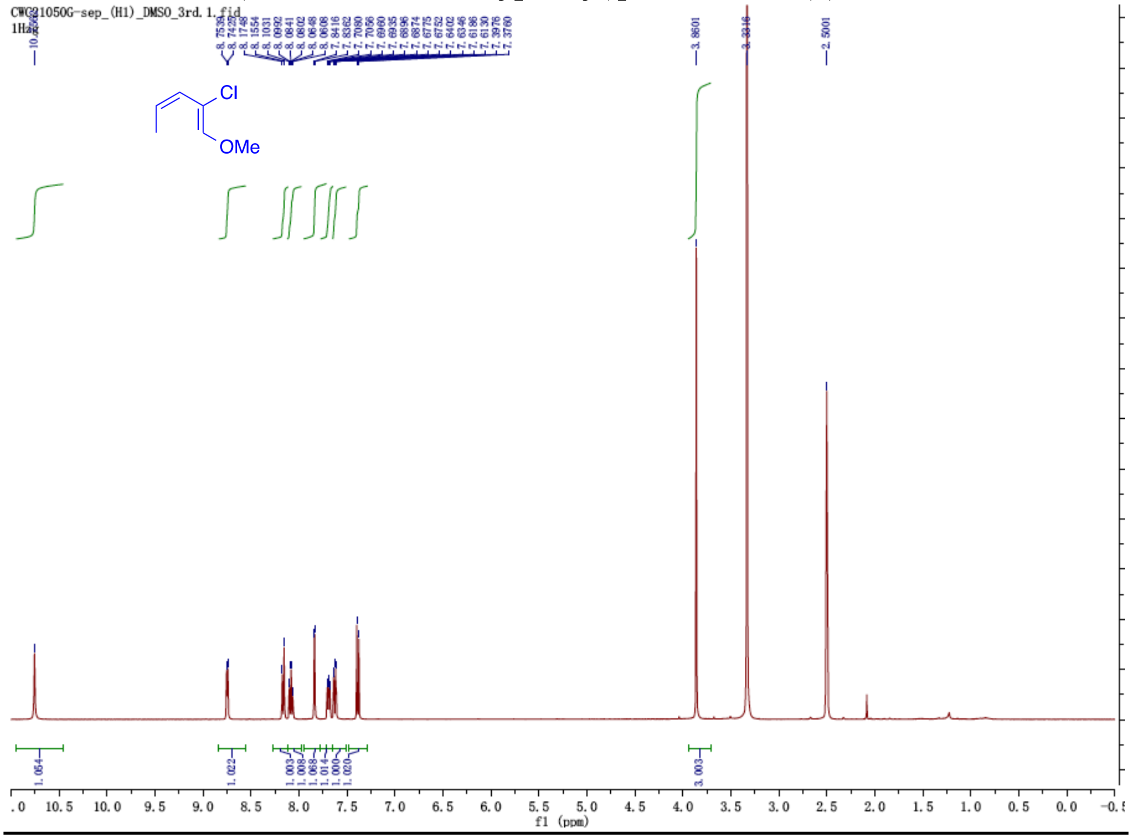

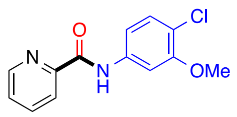

Supplement: Supplementary file 1 [file SC-009-C7SC03950F-s001.pdf]
